# Can diverse population characteristics be leveraged in a machine learning pipeline to predict resource intensive healthcare utilization among hospital service areas?

**DOI:** 10.1186/s12913-022-08154-4

**Published:** 2022-06-30

**Authors:** Iben M. Ricket, Todd A. MacKenzie, Jennifer A. Emond, Kusum L. Ailawadi, Jeremiah R. Brown

**Affiliations:** 1 Department of Epidemiology, Geisel School of Medicine at Dartmouth College, NH Hanover, USA; 2 Department of Biomedical Data Science, Geisel School of Medicine at Dartmouth College, NH Hanover, USA; 3 Norris Cotton Cancer Center, Geisel School of Medicine at Dartmouth College, NH Lebanon, USA; 4 Tuck School of Business at Dartmouth College, NH Hanover, USA

## Abstract

**Background:**

Super-utilizers represent approximately 5% of the population in the United States (U.S.) and yet they are responsible for over 50% of healthcare expenditures. Using characteristics of hospital service areas (HSAs) to predict utilization of resource intensive healthcare (RIHC) may offer a novel and actionable tool for identifying super-utilizer segments in the population. Consumer expenditures may offer additional value in predicting RIHC beyond typical population characteristics alone.

**Methods:**

Cross-sectional data from 2017 was extracted from 5 unique sources. The outcome was RIHC and included emergency room (ER) visits, inpatient days, and hospital expenditures, all expressed as log per capita. Candidate predictors from 4 broad groups were used, including demographics, adults and child health characteristics, community characteristics, and consumer expenditures. Candidate predictors were expressed as per capita or per capita percent and were aggregated from zip-codes to HSAs using weighed means. Machine learning approaches (Random Forrest, LASSO) selected important features from nearly 1,000 available candidate predictors and used them to generate 4 distinct models, including non-regularized and LASSO regression, random forest, and gradient boosting. Candidate predictors from the best performing models, for each outcome, were used as independent variables in multiple linear regression models. Relative contribution of variables from each candidate predictor group to regression model fit were calculated.

**Results:**

The median ER visits per capita was 0.482 [IQR:0.351–0.646], the median inpatient days per capita was 0.395 [IQR:0.214–0.806], and the median hospital expenditures per capita was $2,302 [1$,544.70-$3,469.80]. Using 1,106 variables, the test-set coefficient of determination (R^2^) from the best performing models ranged between 0.184–0.782. The adjusted R^2^ values from multiple linear regression models ranged from 0.311–0.8293. Relative contribution of consumer expenditures to model fit ranged from 23.4–33.6%.

**Discussion:**

Machine learning models predicted RIHC among HSAs using diverse population data, including novel consumer expenditures and provides an innovative tool to predict population-based healthcare utilization and expenditures. Geographic variation in utilization and spending were identified.

**Supplementary Information:**

The online version contains supplementary material available at 10.1186/s12913-022-08154-4.

## Introduction

In 2017, the United States (U.S.) spent $3.5 trillion on healthcare, 33% of which was dedicated to hospital services [1]. Importantly, healthcare utilization and associated spending is not consumed equally [2, 3]. Instead about 5% of the U.S. population, often called “super-utilizers,” are responsible for over 50% of healthcare expenditures [3, 5, 4]. Variation in patterns of healthcare utilization and spending are also seen at the population-level across geographic regions [6–8]. For these reasons, there is tremendous interest in interventions that can curtail healthcare use and associated spending among individual and population-level super-utilizers [2, 4]. A key component of these interventions are models capable of predicting utilization of resource intensive healthcare (RIHC). While several prediction models exist, the unit of analysis for these models is the individual [5, 9, 10]. Extending prior modelling work from the individual to the population by using a geographic unit of analysis offers a novel approach that may complement previous research.

Geographic units are defined by political boundaries (e.g., state, county), administrative areas (e.g., towns), or census units (e.g., census tracts) [11]. While political, administrative, and census units are used in healthcare research, studies suggest hospital service areas (HSAs) offers methodological advantages because HSAs define local hospitalization patterns, and better capture healthcare markets, especially when compared to other geopolitical boundaries [11, 12]. Given this distinction, HSAs are used to study variation in utilization, spending, outcomes, and quality of care in the U.S. and are considered ideal for studies seeking to inform health policy [6, 12–14]. The Dartmouth Atlas defined HSAs and their methods are widely accepted and previously described [6, 12, 15]. Briefly, each HSA is defined by assigning zone improvement plan (ZIP) codes to hospital areas where the greatest proportion of their Medicare residents were hospitalized.

Population risk factors (e.g., age, socioeconomic status, housing instability etc.) are documented drivers of healthcare utilization and when aggregated to the HSA-level may help predict RIHC [3, 16–19]. The power of machine learning allows the opportunity to explore known risk factors for RIHC while also investigating unknown or potentially novel risk factors. To that end, consumer expenditures are data on the purchases of goods and services made by individuals or households [20]. The decision to purchase a good or service is influenced by many variables, and is said to reflect the intersection of income, education, environment, behavior, and preference [21]. Since many of these variables also influence healthcare utilization, it stands to reason that purchased goods and services may offer information on risk for RIHC [16, 17, 19, 22, 23]. In other words, consumer expenditures may represent proxies for more traditional risk factors for RIHC (e.g., income, education, environment). Alternatively, consumer expenditures may also serve as proxies for unobservable or difficult to measure variables or they may reflect goods or behaviors associated with health. For example, expenditures on biking equipment may be a proxy for exercise, access to safe biking infrastructure, or it may reflect an area with higher disposable income. Exercise or lifestyle practices, characteristics of the environment, and income are all associated with healthcare utilization and RIHC [16, 17, 19]. Since consumer expenditures are said to reflect the intersection of many traditional risk factors for RIHC, it’s possible they may provide new information not contained in the traditional risk factors and this new information may be helpful in measuring risk for RIHC.

The objective of this study was to utilize machine learning with diverse population-level data to predict RIHC among HSAs. This approach allows for the examination of disparities in utilization and spending of RIHC within U.S. healthcare markets and provides a novel predictive tool. Since HSAs reflect local healthcare markets and their level of aggregation can capture local healthcare delivery system practices, this predictive tool may direct health policy interventions or inform resource allocation efforts [6, 13]. A second objective was to investigate the predictive and explanatory utility of consumer expenditure data in modeling RIHC among HSAs. Consumer expenditures may serve as an additional determinant of variation in RIHC, offering another data source for researchers, public health practitioners, and policy planners.

## Methods

This cross-sectional, ecological study created an ensemble of models for predicting 3 measures of RIHC utilization among Hospital Service Areas (HSA) in 2017. HSAs were eligible if they had a hospital contributing data to the American Hospital Association (AHA) annual survey in 2017 (*N* ~ 3,100). Data on each eligible HSA came from 5 sources: (1) AHA annual survey, (2) the U.S. Census Bureau (USCB), (3) Centers for Disease Control and Prevention (CDC) (4) the American Community Survey, and (5) Bureau of Labor Statistics (BLS). AHA data were obtained from a licensing agreement with the Dartmouth Analytical Core, while the remaining data were accessed from Data Planet©, a tool from SAGE Publishing, licensed to Dartmouth College [24]. Data Planet© aggregates data across multiple sources, including public domain and licensed data. This study adhered to STROBE reporting guidelines and was exempt from the Dartmouth College institutional review board. All analytical work was performed in R version 3.6.0 (R Foundation).

### Outcomes

Three outcome variables represented utilization of RIHC. All 3 outcomes were extracted from the AHA data and included: (1) total number of inpatient days, defined as the number of adult and pediatric days of care occurring at any hospital type, excluding newborn days or cases, (2) total number of emergency room (ER) visits, defined as the number of emergency department visits at short term general, short-term non-general or long-term hospitals, and (3) hospital expenditures, defined as total hospital expenditures from short-term general, short-term non-general, long-term and Veterans Affairs (VA) hospitals [25]. Data were aggregated to the HSA and all 3 outcomes were expressed as per capita values using population data from 2017, and were log transformed for analysis [26]. Heat maps for each outcome were generated using Tableau and publicly available geographic boundary files from The Dartmouth Atlas [27].

### Candidate Predictors

Four candidate predictor groups were considered: (1) demographics from USCB, (2) adult and child health characteristics from the CDC, (3) community characteristics from the ACS, and (4) consumer expenditures from the BLS. Demographics are based on 2010 Census of Population and Housing, projected to 2017 [28]. Data from the 2010 Census were collected from the entire U.S. population and provide information on age, sex, race, ethnicity, along with basic information on housing characteristics and land area estimates [29]. Adult and child health characteristics are based on the CDC’s national health interview survey for adults and children [30, 31]. Each year, the National Center for Health Statistics (branch of CDC) samples a set of households nationally to provide information on their physical and mental health along with access to routine healthcare services and general health behaviors [32]. Community factors are based on the 2017 ACS, which is administered by the USCB to capture information about local communities characteristics [33, 34]. Consumer expenditure data are based on the nationwide Consumer Expenditure Survey (CEX), which is administered every year to collect information on household expenditures for foods, home goods, and miscellaneous items [30, 35, 36]. The survey covers a broad range of goods and services, including recurring expenses (e.g., rent, loan payments, insurance etc.) along with smaller more frequent purchases (e.g. food, household supplies, clothing etc.), including educational items (e.g. school supplies, uniforms), and healthcare expenses (medical equipment, health insurance) [37].

All candidate predictors were available at the zip-code for 2017 and were aggregated to the HSA using a zip-code-to-HSA crosswalk, publicly available from The Dartmouth Atlas [38]. All candidate predictors were aggregated using weighted average based on the zip-code population to HSA-population. To account for population size, all candidate predictors were expressed as per capita (expenditures, population density) or per capita percent. Data from all candidate predictor groups were merged to each outcome using the HSA number. Prior to model development, all candidate predictors were normalized.

### Predictive Model Development & Implementation

**Fig. 1:**
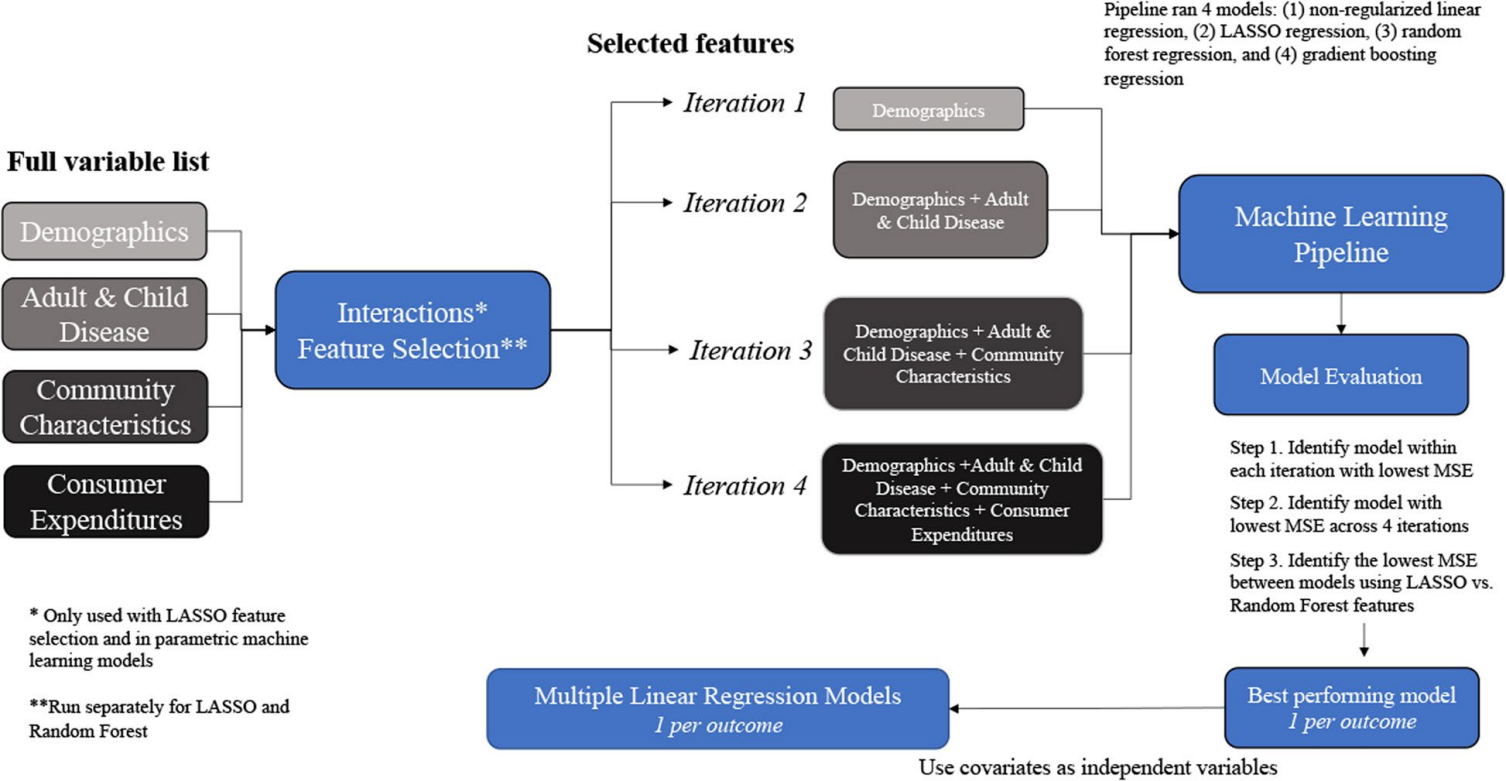
Systematic model development schematic. This approach allowed for evaluation of 4 data inputs, 2 features selection techniques, and 4 machine learning models. Ultimately, it generated 32 models per outcome, for a total of 96 models for the entire study

A systematic model development approach was employed, which allowed for evaluation of data inputs (e.g., candidate predictor groups, including second-order terms), feature selection techniques, and machine learning models (Fig. 1) [39]. To begin, the full data was split into train and test-sets using a 0.80 to 0.20 ratio. The train-set was used to identify second-order terms, perform feature selection, and train machine learning models. The test-set was held-out and used for model evaluation.

Prior to feature selection, second-order terms were generated for use in parametric modeling. Second-order terms were generated using the iml package in R, which leverages random forest and the H-statistics to identify pair-wise interactions explaining variation in each outcome [40]. Model specification for random forest models used to generate second-order terms is found in additional file 1. The feature with the greatest interaction strength was used to generate all pairwise interactions and the pairwise interactions with the greatest interaction strength (top 10%) were retained for parametric feature selection and modeling.

After relevant second-order terms were identified, feature selection was implemented. Least absolute shrinkage and selection operator (LASSO) and random forest were used to perform feature selection, which was applied to each set of candidate predictor variables separately. Inherent to the LASSO algorithm is the ability to perform feature selection [41, 42]. For random forest, features in the top 10% of feature importance were selected for future modeling. Specification for LASSO and random forest feature selection models are available in additional file 1. Variables selected from feature selection were used in the machine learning pipeline, which developed models using non-regularized and LASSO regression, along with random forest and gradient boosting regression. Models were trained using tenfold cross-validation with 5 repeats on the train-set previously derived. Due to sl3 package limitations and computational burden, default hyperparameters were used. Models were evaluated on the full holdout test-set. Additional information on model specification is available in additional file 1. Within the machine learning pipeline, there were 4 iterations, which applied the same models and methods previously described but altered the data inputs across iterations (Fig. 1). Data inputs to machine learning models were the features selected during feature selection. Iteration 1–4 were run twice, first using features selected using LASSO (main effects and second-order terms) and then again using features selected using random forest (main effects only). Iteration 1 included variables from the demographic candidate predictor group and each subsequent iteration (2–4) added variables from a new candidate predictor group. This occurred such that iteration 2 contained variables from 2 candidate predictor groups (i.e. demographics and adult and child health characteristics), iteration 3 contained variables from 3 candidate predictor groups (i.e. demographics, adult and child health characteristics, and community characteristics) and iteration 4 contained variables from all 4 candidate predictor groups (i.e. demographics, adult and child health characteristics, community characteristics, and consumer expenditures). Ultimately, this created a nested approach where each iteration added a new set of variables while retaining those from the previous iteration (Fig. 1).

The best performing model, for each outcome, was identified in 3 main steps using cross-validated mean squared error (MSE), calculated on the test-set. First, within each iteration for a unique feature selection technique, the lowest MSE was retained. This yielded 4 model MSEs (1 per iteration) per feature selection technique, for a total of 8 model MSEs. Second, across the 4 iterations for a unique feature selection technique, the model with the lowest MSE was retained. This yielded 2 model MSEs, 1 per features selection technique. Third, the final 2 model MSEs were compared and the model with the lowest MSE was the best performing model. This approach was employed for each of the 3 outcomes. Observed vs. expected plots were generated for best performing prediction models. In addition, the best performing prediction models were used to identify the top 5% of predicted HSAs for each outcome. Features from the best performing prediction model were used as independent variables in multiple linear regression models (1 for each outcome). Relative contribution of variables from each candidate predictor group to the full multiple linear regression model fit was assessed by measuring the difference in R^2^ from the full model minus the R^2^ from the reduced model containing only variables from 1 of the 4 candidate predictor groups, a method adapted from χ-pie calculations [43].

## Results

**Fig. 2:**
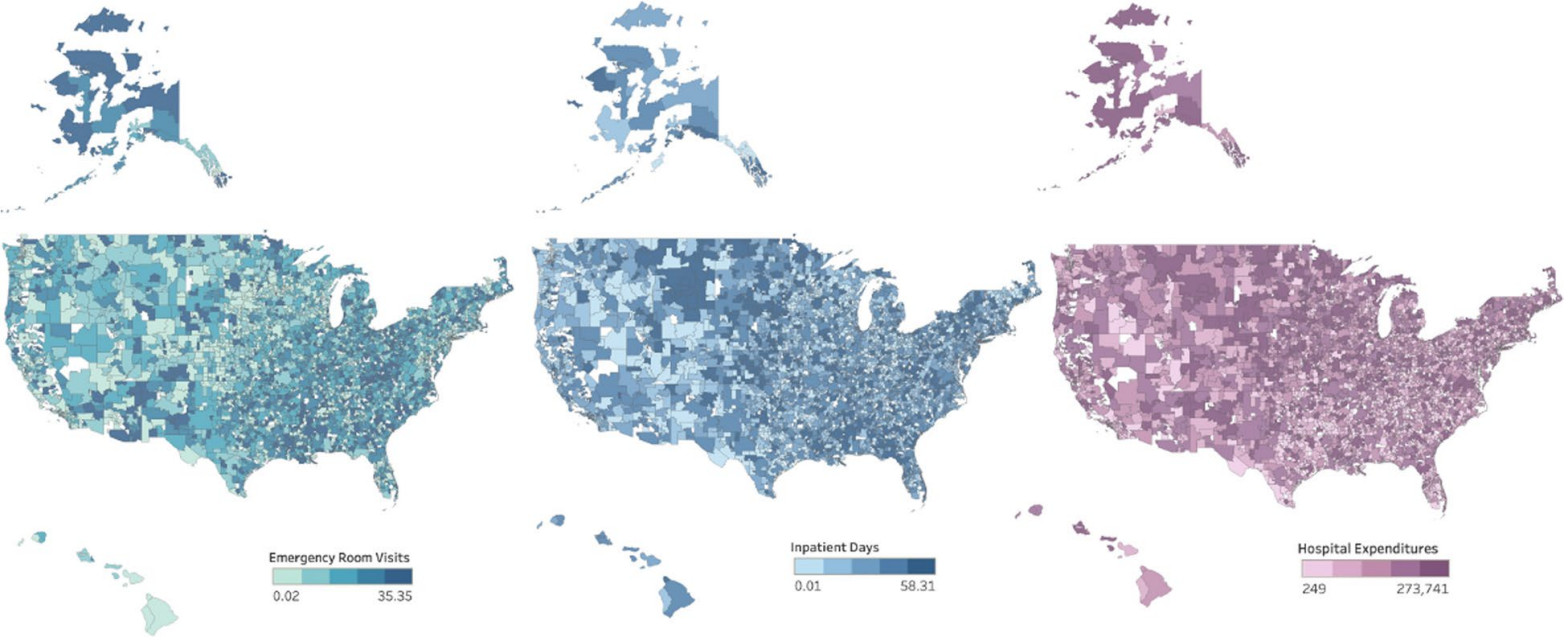
Per capita values for resource intensive healthcare outcomes among Hospital Service Areas. Heat map of annual per capita emergency room visits, inpatient days, and hospital expenditures from hospital service areas in 2017, broken into quintiles. White areas reflect ineligible Hospital Service Areas

This study included 3,153 HSAs with ER services and 3,174 HSAs with inpatient hospital services, representing 91.8% and 92.4% of all HSAs in the U.S., respectively. Median per capita values were as follows: 0.482 ER visits (IQR: 0.351–0.646), 0.395 inpatient days (IQR:0.214–0.806), and $2,302.0 hospital expenditures (IQR: $1554.70-$3469.80). Variation in all 3 outcomes were observed across eligible HSAs (Fig. 2). A total of 1,106 candidate predictors from 4 groups were used in the final machine learning models, including 1,007 main effects and 99 s order terms. Given the number of total candidate predictors, univariate statistics are presented in the online supplemental (additional files 2,3,4,5,6,7,8,9). Univariate statistics for inpatient days and hospital expenditures are the same (N of 3,174 for both), however, estimates of comparable variables for the ER visits outcome are slightly different as the eligible HSA population for this outcome was 3,153 (compared to 3,174). Approximate estimates for select characteristics are provided. Briefly, among all eligible HSAs, the median age per HSA was approximately 43 years, the median family size among HSAs was about 3 people, and 73% of HSA residents were non-Hispanic white. In addition, about 9.7% of the adult population per HSA had heart disease, and nearly 10% of children per HSA had attention deficit hyperactivity disorder. Among employed adults, about 20% per HSA had a commute time to work that was less than 15 min and almost 8% per HSA were employed among healthcare or social assistance fields. On average for each HSA, per capita annual expenditures on food away from the home, laundry equipment, and gardening and lawncare services were $1,252.33, $9.01, and $54.26, respectively.

**Fig. 3:**
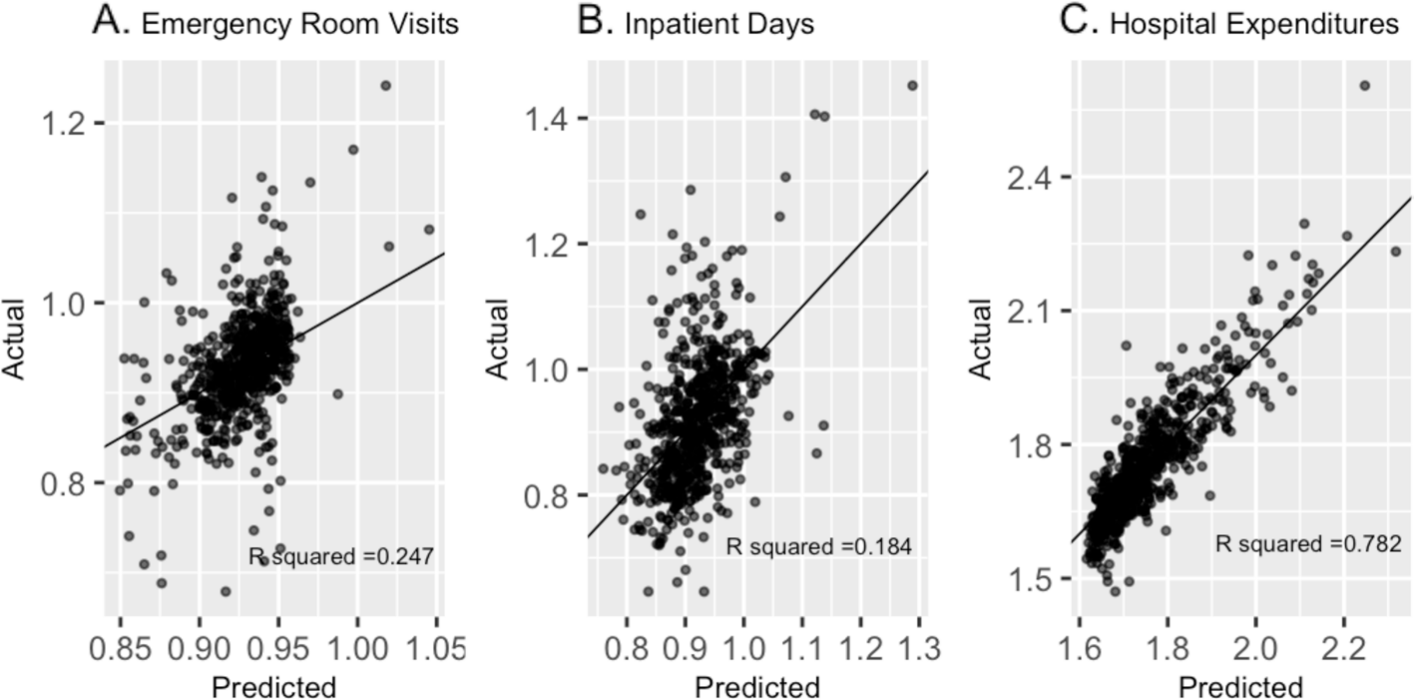
Observed v. Expected plots from best performing prediction models for resource intensive healthcare outcomes. **A** Log Emergency Room Visits per capita | test-set R2 0.247 | test-set MSE: 0.003. **B** Log Inpatient Days per capita | test-set R2 0.184 | test-set MSE:0.011. **C** Log Hospital Expenditures per capita | test-set R2 0.782 | test-set MSE:0.004

**Fig. 4:**
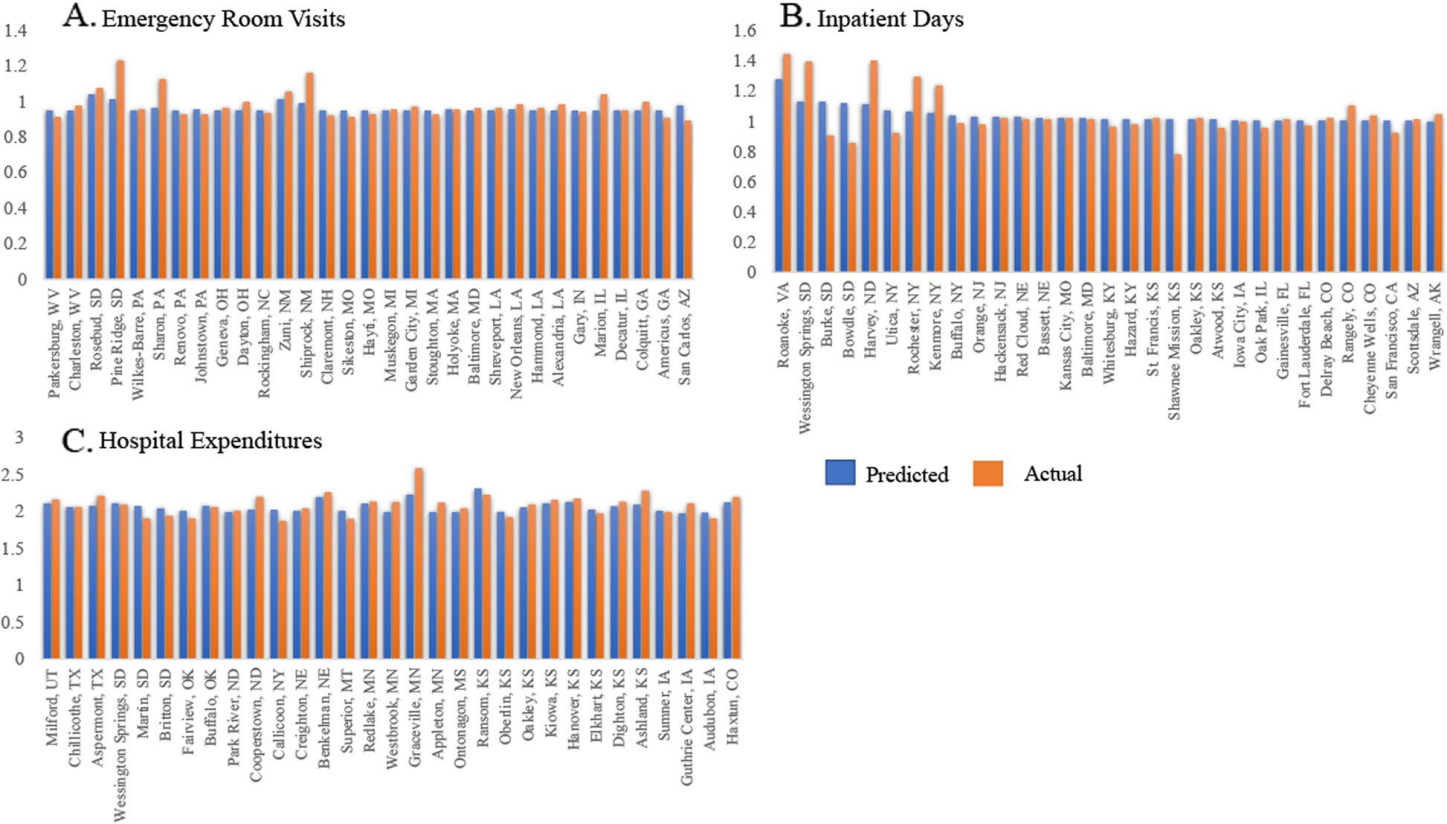
Top 5% of Predicted HSAs from Best Performing Prediction Models for resource intensive healthcare outcomes. **A** 2017 Log Emergency Room Visits per capita. **B** 2017 Log Inpatient Days per capita. **C** 2017 Log Hospital Expenditures per capita

The best performing prediction models across all 3 outcomes used LASSO for feature selection and included variables from all 4 candidate predictor groups (Table 1). Log ER visits per capita (referred to as ‘ER visits’), log inpatient days per capita (referred to as ‘inpatient days’), and log hospital expenditures per capita (referred to as ‘hospital expenditures’) experienced modest-to-good fit across the range of predicted values (Fig. 3). The mean absolute percentage errors for non-log transformed outcomes were as follows: ER visits 3.56%, IP days 76.73%, and hospital expenditures 4.87%. Top 5% of predicted HSAs for inpatient days and hospital expenditures were concentrated among Midwestern and Plains states of the U.S. (Fig. 4B & C). Whereas top 5% of predicted HSAs for ER visits experienced more regional heterogeneity, with representation from HSAs in Southwest and East Coast regions (Fig. 4A). The predicted and actual values at the upper extremes of ER visits and hospital expenditure models, as shown in Fig. 4A & C respectively, highlight the utility of using these models to identify super-utilization. To better visualize model fit at the extremes, Q-Q plots for each outcome are provided in additional files 10 and 11 and confirm fit issues at the upper and lower extremes for ER visit and inpatient day models. Coefficients (or equivalents) from each best performing prediction model are available in additional files 12 (ER visits), 13 (inpatient days), and 14 (hospital expenditures).

**Table 1:** Best Performing Models for resource intensive healthcare outcomes in 2017 among Hospital Service Areas

Outcome (log per capita)	ER Visits (*N* = 3,153)	Inpatient Days (*N* = 3,174)	Hospital Expenditures (*N* = 3,174)
Candidate predictor groups included ^a^	4	4	4
Feature Selection	LASSO^d^	LASSO^d^	LASSO^d^
Model Type	Random Forest	LASSO^d^	Gradient Boosting Machines
MSE^b^	0.003	0.011	0.004
R^2 c^	0.247	0.184	0.782

^a^Candidate predictor groups: 1. Demographics, 2. Adult & Child Health Characteristics, 3. Community Characteristics, and 4. Consumer Expenditure Variables

^b^MSE = mean squared error, calculated on test-set

^c^Coefficient of determination, calculated on test-set

^d^Least Absolute Shrinkage and Selection Operator

Multiple linear regression models (referred to as ‘regression models’) were run using candidate predictors from the best performing prediction model as independent variables. This process was repeated for all 3 outcomes. Due to size, full model output for each outcome is available in additional files 15 (ER visits), 16 (inpatient days), and 17 (hospital expenditures). Table 2 provides abridged model output, generated using the top 5 variables based on the absolute value of the T-statistic. These variables reflect important associations for each outcome. While all 3 outcome variables were log transformed, their coefficients can be multiplied by 100 and roughly be interpreted as a percent increase or decrease in the non-log transformed outcome. For example, a one-unit change in the per capita percent of children within the HSA without a usual place of healthcare corresponds to a 14% decrease in ER visits per capita in the same HSA.

**Table 2:** Abridged ^a^ Model Output from Multiple Linear Regression Models for resource intensive healthcare outcomes

Variable	Coefficient^c^	Standard Error	Z Statistic	*P* Value	Percent Change^c^
***Emergency Room Visits***^***b***^					
% Employees whose commute time to work is between 30–59 min	-0.001	0.000	-4.340	0.000	-0.10%
% Children without a usual place of health care	-0.151	0.038	-3.918	0.000	-14.0%
% Employees whose commute method to work is walking	-0.004	0.001	-3.736	0.000	-0.40%
% of school aged enrolled in private grades 1–4	-0.004	0.001	-3.732	0.000	-0.40%
% Adults never visited doctor	0.659	0.192	3.441	0.001	93.3%
***Inpatient Days***^***b***^					
% Employed within health care or social assistance jobs	0.009	0.002	5.639	0.000	0.90%
% Children with food allergies	-0.320	0.063	-5.083	0.000	-27.4%
% Children whose last dentist visit was more than 5 years ago	0.103	0.027	3.819	0.000	10.8%
% Children whose last health care professional visit was 6 months ago or less	-0.204	0.054	-3.792	0.000	-18.5%
% of population not paying cash for rent	-0.006	0.002	-3.621	0.000	-0.60%
***Hospital Expenditures***^***b***^					
% Employees whose commute time to work is less than 15 min	0.003	0.000	8.630	0.000	0.30%
% Employed within health care or social assistance jobs	0.007	0.001	6.537	0.000	0.70%
Expenditures on men’s nightwear ($/capita)	-0.611	0.101	-6.021	0.000	-45.7%
% Male population 15 + who never married	0.000	0.000	-5.204	0.000	0.0%
% Employed within agriculture, forestry, fishing, or hunting jobs	0.004	0.001	5.091	0.000	0.40%

^a^Model output provided for top 5 variables based on absolute value of T statistic

^b^Expressed as annual 2017 log per capita values

^c^Percent change in non-log transformed outcome, the sign of associated coefficient indicates direction of change

The ER visits regression model included 205 variables, 42 were statistically significant, and the adjusted R^2^ was 0.312 (additional file 15). Consumer expenditure variables offered the greatest relative contribution (33.60%) to the full model fit (Table 3). The most important variables in the ER visits regression model were from adult & child health characteristics and community characteristics candidate predictor groups (Table 2). The per capita percent of employed adults walking to work (*p* < 0.001) was inversely associated with ER visits (Table 2, additional file 15) while the percent of adults who never visited the doctor (*p* = 0.001) was positively associated with ER visits (Table 2, additional file 14).

**Table 3:** Relative Contribution^a^ of Candidate Predictor Groups to Regression Model Fit for resource intensive healthcare outcomes

**Candidate Predictor Domain**	*Emergency Room Visits*^*b*^* (%) *^*c*^	*Inpatient Days*^*b*^* (%) *^*c*^	*Hospital Expenditures*^*b*^* (%) *^*c*^
Demographics	22.70	24.40	18.38
Adult & Child Health Characteristics	23.99	32.13	43.23
Community	19.71	16.46	15.02
Consumer Expenditures	33.60	27.01	23.37

^a^The relative contributions of variables from each candidate predictor group are assessed by measuring the difference in R^2^ from the full model minus the R^2^ from the reduced model containing variables from 1 of the 4 candidate predictor groups

^b^All outcomes expressed as annual log per capita values from 2017

^c^The percentage from each group represents the percent contribution to the full model, for each outcome

The inpatient days regression model included 287 variables, 69 of which were statistically significant, and the adjusted R^2^ was 0.329 (additional file 16). Consumer expenditure variables offered 27.01% to total model fit, however, adult and child health characteristics offered the greatest relative contribution at 32.13% (Table 3). The most important variables in the inpatient days regression model were from adult & child health characteristics and community characteristics candidate predictor groups (Table 2). The per capita percent of children with food allergies (*p* < 0.001) and per capita percent of children with a healthcare visits in the prior 6 months (*p *< 0.001) were inversely associated with inpatient days while per capita percent of adults employed in healthcare or social service fields (*p* < 0.001) was positively associated with inpatient days (Table 2, additional file 16).

Finally, the hospital expenditures regression model included 304 variables, 87 of which were statistically significant, and the adjusted R^2^ was 0.829 (additional file 16). Variables from the adult and child health characteristic group accounted for almost half of the total model fit while consumer expenditures offered 23.37% relative contribution (Table 3). All candidate predictor groups except adult & child health characteristics were represented among the important variables for the hospital expenditures regression model (Table 2). Annual per capita expenditures on men’s nightwear (*p* < 0.001) were inversely associated with hospital expenditures while per capita percent of employed adults working in healthcare or social services fields (*p* < 0.001) was positively associated with hospital expenditures (Table 2, additional file 17).

## Discussion

Using diverse population-level data, this study implemented a machine learning pipeline to predict 3 measures of RIHC. Ultimately, the pipeline predicted RIHC among HSAs with modest performance for ER visits and inpatient day and good performance for hospital expenditures. This suggests some utility in predicting RIHC among healthcare markets and provides an innovative predictive tool to predict population-based healthcare utilization and expenditures. In addition, further analytical work identified important associations between population characteristics, including consumer expenditures, and HSA-level utilization of RIHC. This offers some preliminary evidence for the value of consumer expenditures in studying utilization patterns of RIHC at a population-level.

To our knowledge, this is the first study generating a prediction model for RIHC among HSAs. However, comparable models in the literature are available at the individual level. In terms of model performance, our results were consistent with existing models for hospital expenditures. Caballer, Olmeda, and Consuelo developed models for predicting total healthcare expenditures for a district in Spain and achieved an adjusted R^2^ between 0.46–0.49 [44]. In addition, predictions of healthcare costs using four validated case-mix systems and comorbidity indices were compared using administrative data from British Columbia, achieving R^2^ values between 0.08–0.20 [45]. In this same study, acute care costs were predicted separately with a range of R^2^ values between 0.02–0.06 [45].

All 3 models identified geographic variation in utilization and spending consistent with a large and growing body of literature [6, 7, 14]. The top 5% of predicted HSAs for inpatient days and hospital expenditures were predominately located in the Plains States, a region previously characterized as having above average healthcare spending per capita [8]. Pennsylvania and Louisiana were the most common states among the top 5% of predicted HSAs for ER visits per capita, a finding consistent with both states above average ER visits per capita in 2017, 2018 and 2019, along with their above average healthcare expenditures per capita [8, 46–48]. However, our models did not identify any Alaskan HSAs in the top 5% of predicted HSAs for the ER visits and hospital expenditure models, despite the states long record of high spending and utilization [8, 46–48]. When compared to other states in the U.S., Alaska has unique population characteristics, including its geography, population density, and demographics [49]. In addition, the healthcare market in Alaska also experiences some distinctions that affect provider supply, costs, and access to healthcare [49, 50]. It is possible that the unique aspects of Alaska’s population, especially its geographic isolation and its unique healthcare market are not well characterized by the data used in this project.

Variables from all 4 candidate predictor groups contributed to regression model fit, underscoring the importance of diverse population characteristics in explaining variation in RIHC. Specifically, abridged model outputs identified health status and employment characteristics as important variables explaining variation in all 3 outcomes. Health or disease status is consistently cited as an important risk factor for healthcare utilization, including RIHC [3, 51]. Characteristics of employment is also unsurprising, as health insurance is predominantly employer based in the U.S. and insurance status is associated with healthcare utilization, including RIHC [52, 53]. Together, these results add to a growing body of literature documenting important associations between population characteristics and healthcare utilization. For example, Zhang et al. 2021 found social determinants of health were associated with geographic variation in Medicare spending among U.S. counties and Fitzpatrick et al. found improvement in predicting healthcare utilization with the inclusion of socioeconomic and behavioral health data among a Canadian cohort [7, 17]. Moreover, Wodchis et al. found associations between food insecurity, personal income, and non-homeownership and high utilization of RIHC [17]. Importantly, population characteristics are often modifiable and can serve as targets for interventions. To that end, interventions focused on expanding access to affordable health and dental care coverage for adults and children is one modifiable risk factor to target as an effort to curtail RIHC.

Our study contributes to the literature by (1) using HSAs as the unit of analysis, and (2) using consumer expenditure data. Since HSAs are reflections of local healthcare markets, they are often used to study geographic variation in healthcare utilization and spending [7, 14]. HSAs are often a target of policy interventions aimed at reducing high utilization and spending because their aggregation captures system level factors driving excess use and expenditures [6]. Despite this research, no study to date has used HSAs in models predicting RIHC. Moreover, higher levels of geographic aggregation can mask heterogeneity. For example, when aggregated to the state level, Kansas had approximately 0.80 inpatient days per capita in 2017, however, 5 of the top 5% of predicted HSAs for inpatient days per capita were in Kansas. Since HSAs offer targets for policy interventions and provide granular estimates of geographic variation in healthcare utilization and spending, results from this study suggest this predictive tool can aid policymakers and health system analysts to better plan for resource needs within respected communities.

This study offers one of the first to use consumer expenditures in predicting and explaining variation in RIHC. Across all 3 outcomes, consumer expenditures were included in best performing prediction models and contributed to regression model fit. While these findings are preliminary, they lend some support for including consumer expenditures when studying RIHC. While the use of consumer expenditure data in the context of healthcare research is relatively novel, results from one prior study conducted by SAS® (Cary, NC) determined consumer expenditures improved models predicting healthcare utilization and associated costs, generally aligning with results from our study [54], (Ricket et al. : Novel integration of governmental data sources using machine learning to identify super-utilization among U.S. counties, submitted). Importantly, results from this study represent preliminary findings and should be interpreted with caution as this study cannot address causality between consumer expenditures and RIHC. Results provide early evidence to support continued research on the utility of consumer expenditures to study healthcare utilization, however, future studies are needed to confirm these findings and explore possible mechanisms.

Results from this study have several implications. First, machine learning models can be leveraged as a tool to predict geographic variation in healthcare utilization and spending. Such a tool can help policy planners identify healthcare markets in need of policy initiatives or community-based interventions. Second, population characteristics associated with RIHC can serve as modifiable targets for future interventions. Third, results from this study suggest some value in using consumer expenditures to study RIHC. Since these variables are routinely collected, they represent a potential new data source for health service researchers to explore for future research.

## Limitations

While this work offers novel insights into the power of leveraging vast data resources to predict RIHC, the work is not without limitations. First, this is an ecological study and as such, findings cannot address individual-level factors associated with RIHC. Moreover, this study cannot comment on longitudinally or temporal trends as it utilizes cross-sectional data. Moreover, cross-sectional findings from this study may not be robust overtime. Future research seeks to integrate more years of data. Despite these limitations, this study uses high-quality data from reputable governmental and non-governmental sources. In addition, this study only included HSAs participating in the 2017 AHA annual survey, however, over 90% of all HSAs were included. Separately, this study did not include physician supply, which could affect the 3 outcomes in this study. The documented association between physician supply and healthcare utilization are mixed, however, several recent and noteworthy studies identified no significant relationship [55]. In addition, the stability of variables selected from LASSO presents another limitation, as the primary objective of LASSO is to select variables with highest prediction [56]. Similarly, the use of adaptative LASSO is generally considered an improvement upon standard LASSO, however, results from a small sensitivity analysis found no difference in model performance when using adaptive LASSO for feature selection [56]. Despite this, future research endeavors should consider the advantages conveyed by using adaptive LASSO [56]. Lastly, aggregation to HSA may limit generalizability to countries outside of the U.S, especially areas where healthcare systems and the management of hospital care differs vastly from the U.S.

## Conclusion

Data from 5 unique sources were leveraged in a machine learning pipeline to predict 3 metrics of RIHC, including ER visits, inpatient days, and hospital expenditures. The novel machine learning prediction tool provides an innovative approach to predicting population-based healthcare utilization and associated spending. Disease status and employment characteristics were important variables explaining variation in RIHC and serve as modifiable targets for future interventions.

## Supplementary Information

**Additional file 1.** 

**Additional file 2.** 

**Additional file 3.** 

**Additional file 4.** 

**Additional file 5.** 

**Additional file 6.** 

**Additional file 7.**

**Additional file 8.** 

**Additional file 9.** 

**Additional file 10.** 

**Additional file 11.** 

**Additional file 12.** 

**Additional file 13.** 

**Additional file 14.** 

**Additional file 15.**

**Additional file 16.** 

**Additional file 17.** 

## Data Availability

The data that support the findings of this study are available from Data Planet© from SAGE publishing and the American Hospital Association but restrictions apply to the availability of these data, which were used under license for the current study, and so are not publicly available. Data are however available from the authors upon reasonable request and with permission of Data Planet© from SAGE publishing and the American Hospital Association.
