# Supplementary material for: Can diverse population characteristics be leveraged in a machine learning pipeline to predict resource intensive healthcare utilization among hospital service areas?
Source: BMC Health Serv Res. 2022 Jun 30;22:847. doi: 10.1186/s12913-022-08154-4 (PMC9248096; doi:10.1186/s12913-022-08154-4)
Supplement: Supplementary file 1 — Additional file 1. [file 12913_2022_8154_MOESM1_ESM.pdf]

Additional File 1. Model Specification for LASSO & Random Forest Models

- Additional File 1
  - File format: PDF
  - File title: Model Specification for LASSO and Random Forest Models
  - File description: Details on methods

Random Forest Machine Learning Model & Feature Selection Information	
Number of drawn candidate variables in each split	14.31 (ER), 16.94 (IP), 17.43 (HosExp)
Sample size of observations	3,153 (ER), 3,174 (IP & HosExp)
Whether observations were drawn with replacement	TRUE
Node size	5
Number of trees	500
Splitting rule	Minimize SSE
R packaged utilized	Random Forest & sl3
LASSO Feature Selection Information	
Alpha	1
Lambda	Lambda minimum
R package utilized	glmnet
Regularized Machine Learning Model Information	
Alpha	1 (Tuned)
Lambda	Lambda minimum
R packaged utilized	Glment, Sl3

ER= emergency room visits
IP= inpatient days
HosExp = hospital expenditures

Default hyperparameters were utilized
