# Supplementary material for: Can diverse population characteristics be leveraged in a machine learning pipeline to predict resource intensive healthcare utilization among hospital service areas?
Source: BMC Health Serv Res. 2022 Jun 30;22:847. doi: 10.1186/s12913-022-08154-4 (PMC9248096; doi:10.1186/s12913-022-08154-4)
Supplement: Supplementary file 2 — Additional file 2. [file 12913_2022_8154_MOESM2_ESM.pdf]

# Additional File 2. Descriptive Statistics for Demographic Candidate Predictors (Main Effects)

- Additional File 2
  - File format: PDF
  - File title: Descriptive Statistics for Demographic Candidate Predictors (Main Effects)
  - File description: Long table with univariate results for main effects

| | ER Visits | Inpatient Days & Hospital Expenditures |
|---|---|---|
| Total Hospital Service Areas (N) | 3153 | 3174 |
| census demographics 2017 household income average | 71383.24 (80267.55) | 71659.17 (80609.51) |
| census demographics 2017 population speaks english pop 5 persons | 87.0 (14.0) | 87.0 (14.0) |
| census demographics 2017 population speaks spanish pop 5 persons | 8.0 (12.0) | 8.0 (12.0) |
| census demographics 2017 population speaks other language pop 5 persons | 1.0 (4.0) | 1.0 (4.0) |
| census demographics 2017 population citizenship native persons | 93.0 (8.0) | 93.0 (8.0) |
| census demographics 2017 population citizenship foreign born naturalized persons | 3.0 (4.0) | 3.0 (4.0) |
| census demographics 2017 population citizenship foreign born not a citizen persons | 4.02 (4.75) | 4.04 (4.76) |
| census demographics 2017 veterans total persons | 9.77 (3.07) | 9.75 (3.08) |
| census demographics 2017 population total group quarters persons | 3.46 (4.19) | 3.46 (4.18) |
| census demographics 2017 population institutional group quarters persons | 5511.06 (16307.85) | 5.56 (1.64) |
| census demographics 2017 population non institutional group quarters persons | 0.00 (0.01) | 0.00 (0.01) |
| census demographics 2017 householder aged under 25 years households | 1.86 (1.36) | 1.86 (1.36) |
| census demographics 2017 householder aged 25 to 34 years households | 5.40 (1.24) | 5.40 (1.25) |
| census demographics 2017 householder aged 35 to 44 years households | 6.14 (0.85) | 6.14 (0.85) |
| census demographics 2017 householder aged 45 to 54 years households | 7.88 (0.97) | 7.88 (0.97) |
| census demographics 2017 householder aged 55 to 64 years households | 8.24 (1.42) | 8.24 (1.42) |
| census demographics 2017 householder aged 65 to 74 years households | 5.82 (1.57) | 5.82 (1.57) |
| census demographics 2017 householder aged 75 to 84 years households | 3.61 (1.23) | 3.61 (1.23) |
| census demographics 2017 householder aged 85 years and over households | 1.67 (0.76) | 1.67 (0.76) |
| census demographics 2017 households with no vehicles households | 3.02 (1.75) | 3.02 (1.75) |
| census demographics 2017 households with 1 vehicle households | 13.38 (2.84) | 13.38 (2.84) |
| census demographics 2017 households with 2 vehicles households | 15.07 (2.26) | 15.06 (2.26) |

| | | |
|---|---|---|
| census demographics 2017 households with 3 vehicles households | 6.25 (1.67) | 6.24 (1.66) |
| census demographics 2017 households with 4 vehicles households | 2.91 (1.32) | 2.90 (1.32) |
| census demographics 2017 population density persons per sq mile | 13782.65 (65567.45) | 14040.06 (65613.88) |
| census demographics 2017 education enrolled public or private school pop 3 persons | 80.52 (7.46) | 80.53 (7.45) |
| census demographics 2017 education enrolled public preprimary pop 3 persons | 3.59 (1.83) | 3.59 (1.82) |
| census demographics 2017 education enrolled private preprimary pop 3 persons | 1.75 (1.28) | 1.75 (1.28) |
| census demographics 2017 education enrolled public kindergarten pop 3 persons | 3.94 (1.48) | 3.94 (1.48) |
| census demographics 2017 education enrolled private kindergarten pop 3 persons | 0.44 (0.44) | 0.44 (0.44) |
| census demographics 2017 education enrolled public grades 1 4 pop 3 persons | 15.03 (3.45) | 15.02 (3.45) |
| census demographics 2017 education enrolled private grades 1 4 pop 3 persons | 1.52 (1.26) | 1.52 (1.26) |
| census demographics 2017 education enrolled public grades 5 8 pop 3 persons | 15.45 (3.71) | 15.45 (3.71) |
| census demographics 2017 education enrolled private grades 5 8 pop 3 persons | 1.51 (1.25) | 1.51 (1.24) |
| census demographics 2017 education enrolled public grades 9 12 pop 3 persons | 15.88 (3.85) | 15.88 (3.85) |
| census demographics 2017 education enrolled private grades 9 12 pop 3 persons | 1.41 (1.17) | 1.41 (1.17) |
| census demographics 2017 education enrolled public undergraduate college pop 3 persons | 13.46 (8.59) | 13.46 (8.57) |
| census demographics 2017 education enrolled private undergraduate college pop 3 persons | 3.45 (4.21) | 3.46 (4.23) |
| census demographics 2017 education enrolled public graduate or professional school pop 3 persons | 1.99 (1.70) | 1.99 (1.70) |
| census demographics 2017 education enrolled private graduate or professional school pop 3 persons | 1.10 (1.29) | 1.10 (1.30) |
| census demographics 2017 education not enrolled in school pop 3 persons | 235.72 (53.24) | 2.3564 (0.05) |
| census demographics 2017 education attainment high school pop 25 persons | 14.00 (6.72) | 14.00 (6.72) |
| census demographics 2017 education attainment high school pop 25 persons 2 | 32.06 (7.62) | 32.04 (7.63) |
| census demographics 2017 education attainment some college pop 25 persons | 22.37 (4.37) | 22.36 (4.37) |
| census demographics 2017 population in poverty total persons | 16.58 (6.76) | 16.57 (6.76) |
| census demographics 2017 education attainment associate s degree pop 25 persons | 8.45 (2.50) | 8.45 (2.50) |
| census demographics 2017 education attainment bachelor s degree pop 25 persons | 14.83 (6.25) | 14.85 (6.27) |
| census demographics 2017 education attainment master s degree pop 25 persons | 5.96 (3.47) | 5.97 (3.48) |

| | | |
|---|---|---|
| census demographics 2017 education attainment professional degree pop 25 persons | 1.39 (1.12) | 1.40 (1.12) |
| census demographics 2017 education attainment doctorate degree pop 25 persons | 0.00 (0.00) | 0.00 (0.00) |
| census demographics 2017 households with income less than 15000 households | 4.78 (1.95) | 4.78 (1.95) |
| census demographics 2017 households with income 15000 to 24999 households | 3.78 (1.19) | 3.78 (1.19) |
| census demographics 2017 households with income 25000 to 34999 households | 3.91 (1.05) | 3.90 (1.05) |
| census demographics 2017 households with income 35000 to 49999 households | 5.21 (1.15) | 5.20 (1.15) |
| census demographics 2017 households with income 50000 to 74999 households | 6.91 (1.24) | 6.91 (1.24) |
| census demographics 2017 households with income 75000 to 99999 households | 5.37 (0.96) | 5.36 (0.96) |
| census demographics 2017 households with income 100000 to 124999 households | 3.79 (0.83) | 3.79 (0.83) |
| census demographics 2017 households with income 125000 to 149999 households | 2.46 (0.77) | 2.46 (0.77) |
| census demographics 2017 households with income 150000 to 199999 households | 2.12 (1.05) | 2.12 (1.05) |
| census demographics 2017 households with income 200000 and over households | 2.30 (2.00) | 2.31 (2.01) |
| census demographics 2017 households 1 person households | 11.76 (3.00) | 11.75 (3.00) |
| census demographics 2017 households 2 person households | 13.95 (2.77) | 13.94 (2.77) |
| census demographics 2017 households 3 person households | 6.08 (0.85) | 6.08 (0.85) |
| census demographics 2017 households 4 person households | 4.81 (0.74) | 4.82 (0.74) |
| census demographics 2017 households 5 person households | 2.39 (0.49) | 2.39 (0.49) |
| census demographics 2017 households 6 person households | 0.97 (0.34) | 0.97 (0.34) |
| census demographics 2017 households 7 or more person households | 0.65 (0.44) | 0.65 (0.44) |
| census demographics 2017 families married with children under 18 families | 29.07 (5.63) | 29.08 (5.63) |
| census demographics 2017 household average size persons | 52.07 (56.59) | 5.228 (5.673) |
| census demographics 2017 head of household male households | 24.63 (3.36) | 24.61 (3.36) |
| census demographics 2017 head of household female households | 15.99 (2.52) | 15.99 (2.52) |
| census demographics 2017 family head of household male households | 67.46 (7.35) | 67.44 (7.34) |
| census demographics 2017 family head of household female households | 32.54 (7.35) | 32.56 (7.34) |
| census demographics 2017 families married families | 73.16 (7.63) | 73.13 (7.63) |
| census demographics 2017 other families male householder no wife present other families | 30.15 (5.64) | 30.13 (5.63) |
| census demographics 2017 other families female householder no husband present other families | 69.85 (5.64) | 69.87 (5.63) |
| census demographics 2017 families married with no children under 18 families | 44.09 (7.66) | 44.05 (7.66) |

| | | |
|---|---|---|
| census demographics 2017 other families male householder no wife present with children under 18 other families | 18.34 (4.74) | 18.32 (4.74) |
| census demographics 2017 other families male householder no wife present with no children under 18 other families | 11.80 (2.65) | 11.81 (2.65) |
| census demographics 2017 other families female householder no husband present with children under 18 other families | 45.55 (5.88) | 45.54 (5.90) |
| census demographics 2017 other families female householder no husband present with no children under 18 other families | 24.30 (5.14) | 24.33 (5.16) |
| census demographics 2017 non family head of household male households | 47.54 (4.43) | 47.53 (4.42) |
| census demographics 2017 non family head of household female households | 52.46 (4.43) | 52.47 (4.42) |
| census demographics 2017 non family households male householder with people under 18 households | 0.89 (0.44) | 0.89 (0.43) |
| census demographics 2017 non family households male householder with no people under 18 households | 46.62 (4.53) | 46.61 (4.53) |
| census demographics 2017 non family households female householder with people under 18 households | 0.24 (0.16) | 0.24 (0.16) |
| census demographics 2017 non family households female householder with no people under 18 households | 52.24 (4.60) | 52.26 (4.60) |
| census demographics 2017 population urban persons | 60.98 (31.71) | 61.11 (31.72) |
| census demographics 2017 population rural persons | 39.02 (31.71) | 38.89 (31.72) |
| census demographics 2017 population male persons | 49.57 (1.98) | 49.57 (1.97) |
| census demographics 2017 population female persons | 50.43 (1.98) | 50.43 (1.97) |
| census demographics 2017 white population alone persons | 79.21 (18.00) | 79.12 (18.04) |
| census demographics 2017 black population alone persons | 8.95 (13.86) | 9.00 (13.91) |
| census demographics 2017 asian population alone persons | 2.63 (5.22) | 2.65 (5.28) |
| census demographics 2017 american indian and alaska native population alone persons | 2.15 (8.35) | 2.14 (8.32) |
| census demographics 2017 other race population alone persons | 0.00 (0.01) | 0.00 (0.01) |
| census demographics 2017 two or more races population persons | 9.79 (2.55) | 9.88 (2.54) |
| census demographics 2017 hispanic population persons | 11.12 (15.00) | 11.14 (15.00) |
| census demographics 2017 white non hispanic population persons | 73.17 (22.18) | 73.07 (22.21) |
| census demographics 2017 white households households | 34.02 (8.65) | 33.98 (8.66) |
| census demographics 2017 black households households | 3.24 (5.27) | 3.26 (5.29) |
| census demographics 2017 asian households households | 0.82 (1.69) | 0.83 (1.71) |
| census demographics 2017 american indian and alaska native head of households households | 0.70 (2.48) | 0.69 (2.47) |
| census demographics 2017 other race head of households persons | 1.14 (1.59) | 1.14 (1.58) |
| census demographics 2017 families 1 person families | 0.00 (0.00) | 0.00 (0.00) |
| census demographics 2017 two or more races head of households households | 0.71 (0.56) | 0.71 (0.56) |
| census demographics 2017 families 2 person families | 44.96 (7.71) | 44.92 (7.71) |

| | | |
|---|---|---|
| census demographics 2017 families 3 person families | 21.97 (3.00) | 21.99 (3.00) |
| census demographics 2017 families 4 person families | 17.85 (2.87) | 17.86 (2.87) |
| census demographics 2017 families 5 person families | 9.01 (2.19) | 9.01 (2.19) |
| census demographics 2017 families 6 person families | 3.70 (1.50) | 3.70 (1.50) |
| census demographics 2017 families 7 or more person families | 2.50 (2.00) | 2.51 (2.00) |
| census demographics 2017 non families 1 person households | 82.46 (6.10) | 82.45 (6.09) |
| census demographics 2017 non families 2 person households | 14.19 (3.96) | 14.19 (3.95) |
| census demographics 2017 non families 3 person households | 2.03 (1.46) | 2.03 (1.45) |
| census demographics 2017 hispanic households persons | 3.00 (4.28) | 3.00 (4.27) |
| census demographics 2017 non families 4 person households | 0.87 (1.04) | 0.87 (1.04) |
| census demographics 2017 non families 5 person households | 0.27 (0.34) | 0.28 (0.34) |
| census demographics 2017 non families 6 person households | 0.11 (0.56) | 0.11 (0.56) |
| census demographics 2017 non families 7 or more person households | 0.07 (0.15) | 0.07 (0.15) |
| census demographics 2017 families aged under 25 years families | 3.29 (1.53) | 3.29 (1.54) |
| census demographics 2017 families aged 25 to 34 years families | 14.55 (3.36) | 14.54 (3.36) |
| census demographics 2017 families aged 35 to 44 years families | 18.52 (3.03) | 18.52 (3.02) |
| census demographics 2017 families aged 45 to 54 years families | 21.17 (2.93) | 21.18 (2.94) |
| census demographics 2017 families aged 55 to 64 years families | 20.22 (2.58) | 20.22 (2.58) |
| census demographics 2017 white non hispanic households households | 32.32 (9.88) | 32.27 (9.89) |
| census demographics 2017 families aged 65 to 74 years families | 13.43 (3.13) | 13.42 (3.12) |
| census demographics 2017 families aged 75 years and over families | 8.84 (2.63) | 8.83 (2.62) |
| census demographics 2017 non families aged under 25 years households | 6.55 (5.82) | 6.54 (5.81) |
| census demographics 2017 non families aged 25 to 34 years households | 11.12 (4.28) | 11.14 (4.29) |
| census demographics 2017 non families aged 35 to 44 years households | 9.15 (2.31) | 9.16 (2.31) |
| census demographics 2017 non families aged 45 to 54 years households | 16.36 (2.86) | 16.36 (2.85) |
| census demographics 2017 non families aged 55 to 64 years households | 20.44 (3.25) | 20.44 (3.25) |
| census demographics 2017 non families aged 65 to 74 years households | 15.94 (3.44) | 15.94 (3.44) |
| census demographics 2017 non families aged 75 years and over households | 20.43 (5.84) | 20.42 (5.83) |
| census demographics 2017 population median age years | 42.80 (4.86) | 42.78 (4.86) |
| census demographics 2017 householder median age years | 54.13 (3.26) | 54.12 (3.27) |
| census demographics 2017 families median age years | 52.16 (3.06) | 52.15 (3.07) |
| census demographics 2017 family median size number persons | 3.18 (0.31) | 3.18 (0.31) |
| census demographics 2017 households median size number persons | 2.87 (0.29) | 2.88 (0.29) |
| census demographics 2017 families median age years | 52.16 (3.06) | 52.14 (3.07) |

HH=Household
Fam=Family
Pop=Population
Non Fam=Non family
OT=Other
ER=Emergency room

RV=recreational vehicle
Equip=equipment
Misc.=miscellaneous
BCBS=Blue Cross Blue Shield
OOT=Out of town
RIHC=resource intensive healthcare
