# Supplementary material for: Can diverse population characteristics be leveraged in a machine learning pipeline to predict resource intensive healthcare utilization among hospital service areas?
Source: BMC Health Serv Res. 2022 Jun 30;22:847. doi: 10.1186/s12913-022-08154-4 (PMC9248096; doi:10.1186/s12913-022-08154-4)
Supplement: Supplementary file 3 — Additional file 3. [file 12913_2022_8154_MOESM3_ESM.pdf]

Additional File 3. Descriptive Statistics for Demographic Candidate Predictors (second order terms)

- Additional File 3
  - File format: PDF
  - File title: Descriptive Statistics for Demographic Candidate Predictors (second order terms)
  - File description: Long table with univariate results for second order terms

ER Visits (mean (sd))	
census demographics 2017 household income average census demographics 2017 household average size persons	13961.83 (35283.96)
census demographics 2017 population non institutional group quarters persons census demographics 2017 household average size persons	0.0003 (0.0013)
census demographics 2017 householder aged under 25 years households census demographics 2017 household average size persons	0.86 (1.08)
census demographics 2017 population density persons per sq mile census demographics 2017 household average size persons	13711.10(121312.51)
census demographics 2017 education enrolled private undergraduate college pop 3 persons census demographics 2017 household average size persons	1.54 (3.93)
census demographics 2017 education attainment doctorate degree pop 25 persons census demographics 2017 household average size persons	0.0003 (0.0005)
census demographics 2017 non family head of household female households census demographics 2017 household average size persons	27.05 (29.24)
census demographics 2017 population urban persons census demographics 2017 household average size persons	27.35 (37.51)
census demographics 2017 population rural persons census demographics 2017 household average size persons	24.71 (40.67)
census demographics 2017 black population alone persons census demographics 2017 household average size persons	4.52 (12.20)
census demographics 2017 non families aged 25 to 34 years households census demographics 2017 household average size persons	5.27 (6.00)
census demographics 2017 non families aged 65 to 74 years households census demographics 2017 household average size persons	8.62 (10.01)
Inpatient Days (mean (sd))	
census demographics 2017 household income average census demographics 2017 household average size persons	1421719.54 (3507123.29)
census demographics 2017 population non institutional group quarters persons census demographics 2017 household average size persons	0.03 (0.13)

census demographics 2017 population density persons per sq mile census demographics 2017 household average size persons	1088937.99 (9932172.78)
census demographics 2017 education attainment professional degree pop 25 persons census demographics 2017 household average size persons	62.88 (128.54)
census demographics 2017 education attainment doctorate degree pop 25 persons census demographics 2017 household average size persons	0.03 (0.05)
census demographics 2017 head of household male households census demographics 2017 household average size persons	1321.07 (1431.79)
census demographics 2017 family head of household female households census demographics 2017 household average size persons	1684.66 (1923.53)
census demographics 2017 other families male householder no wife present with children under 18 other families census demographics 2017 household average size persons	1015.74 (1193.56)
census demographics 2017 population urban persons census demographics 2017 household average size persons	2735.55 (3710.78)
census demographics 2017 black population alone persons census demographics 2017 household average size persons	474.09 (1291.30)
census demographics 2017 other race population alone persons census demographics 2017 household average size persons	0.12 (0.32)
census demographics 2017 families aged under 25 years families census demographics 2017 household average size persons	179.34 (231.32)
Hospital Expenditures (mean (sd))	
census demographics 2017 household income average census demographics 2017 household average size persons	1421719.54 (3507123.29)
census demographics 2017 population non institutional group quarters persons census demographics 2017 household average size persons	0.03 (0.13)
census demographics 2017 population density persons per sq mile census demographics 2017 household average size persons	1088937.99 (9932172.78)
census demographics 2017 education attainment professional degree pop 25 persons census demographics 2017 household average size persons	62.88 (128.54)
census demographics 2017 education attainment doctorate degree pop 25 persons census demographics 2017 household average size persons	0.03 (0.05)
census demographics 2017 head of household male households census demographics 2017 household average size persons	1321.07 (1431.79)
census demographics 2017 family head of household female households census demographics 2017 household average size persons	1684.66 (1923.53)
census demographics 2017 other families male householder no wife present with children under 18 other families census demographics 2017 household average size persons	1015.74 (1193.56)

census demographics 2017 population urban persons census demographics 2017 household average size persons	2735.55 (3710.78)
census demographics 2017 black population alone persons census demographics 2017 household average size persons	474.09 (1291.30)
census demographics 2017 other race population alone persons census demographics 2017 household average size persons	0.12 (0.32)
census demographics 2017 families aged under 25 years families census demographics 2017 household average size persons	179.34 (231.32)

HH=Household
Fam=Family
Pop=Population
Non Fam=Non family
OT=Other
ER=Emergency room
RV=recreational vehicle
Equip=equipment
Misc.=miscellaneous
BCBS=Blue Cross Blue Shield
OOT=Out of town
RIHC=resource intensive healthcare
