# Supplementary material for: Can diverse population characteristics be leveraged in a machine learning pipeline to predict resource intensive healthcare utilization among hospital service areas?
Source: BMC Health Serv Res. 2022 Jun 30;22:847. doi: 10.1186/s12913-022-08154-4 (PMC9248096; doi:10.1186/s12913-022-08154-4)
Supplement: Supplementary file 4 — Additional file 4. [file 12913_2022_8154_MOESM4_ESM.pdf]

Additional File 4. Descriptive Statistics for Adult & Child Health Characteristics (Main effects)

- Additional File 4
  - File format: PDF
  - File title: Descriptive Statistics for Adult & Child Health Characteristics (Main effects)
  - File description: Long table with univariate results for main effects

	ER	Inpatient Days & Hospital Expenditures
Hospital Service Area (N)	3153	3174
hsanum	26296.63 (14813.77)	26281.36 (14809.53)
health adults 2017 aerobic guidelines only inactive count of households persons	23.62 (1.59)	23.62 (1.59)
health adults 2017 aerobic guidelines only insufficiently active count of households persons	15.36 (0.70)	15.36 (0.70)
health adults 2017 aerobic guidelines only sufficiently active met guidelines count of households persons	37.63 (1.87)	37.63 (1.87)
health adults 2017 full guidelines strengthening and aerobic combined met neither count of households persons	36.32 (2.00)	36.32 (1.99)
health adults 2017 full guidelines strengthening and aerobic combined met muscle strengthening only count of households persons	2.68 (0.15)	2.68 (0.15)
health adults 2017 full guidelines strengthening and aerobic combined met aerobic only count of households persons	21.55 (1.14)	21.55 (1.14)
health adults 2017 full guidelines strengthening and aerobic combined met both muscle strengthening and aerobic guidelines count of households persons	16.03 (0.91)	16.03 (0.92)
health adults 2017 serious psychological distress count of households persons	2.70 (0.29)	2.70 (0.29)
health adults 2017 type hospital emergency room count of households persons	0.69 (0.09)	0.69 (0.09)
health adults 2017 type hospital outpatient department count of households persons	0.78 (0.08)	0.78 (0.08)
health adults 2017 has unmet dental need due to cost count of households persons	8.47 (0.85)	8.47 (0.85)
health adults 2017 does not have unmet dental need due to cost count of households persons	68.58 (3.14)	68.58 (3.14)

health adults 2017 all types heart disease count of households persons	9.66 (1.08)	9.66 (1.08)
health adults 2017 last dental visit 6 months or less count of households persons	36.33 (2.37)	36.34 (2.38)
health adults 2017 last dental visit more than six months but not more than 1 year ago count of households persons	12.43 (0.55)	12.44 (0.55)
health adults 2017 last dental visit more than 1 year but not more than 2 years ago count of households persons	9.45 (0.48)	9.45 (0.48)
health adults 2017 last dental visit more than 2 years ago but not more than 5 years ago count of households persons	8.49 (0.60)	8.49 (0.60)
health adults 2017 last dental visit more than 5 years ago excluding never count of households persons	9.31 (1.01)	9.31 (1.01)
health adults 2017 last dental visit never count of households persons	0.71 (0.05)	0.71 (0.05)
health adults 2017 hiv ever tested count of households persons	27.80 (1.64)	27.80 (1.64)
health adults 2017 hiv never tested count of households persons	45.95 (2.80)	45.95 (2.80)
health adults 2017 any cancer count of households persons	7.38 (1.08)	7.38 (1.08)
health adults 2017 breast cancer count of households persons	1.34 (0.15)	1.34 (0.15)
health adults 2017 cervical cancer count of households persons	0.34 (0.02)	0.34 (0.02)
health adults 2017 prostate cancer count of households persons	0.91 (0.14)	0.91 (0.14)
health adults 2017 diabetes count of households persons	7.81 (0.88)	7.81 (0.88)
health adults 2017 ulcers count of households persons	4.87 (0.36)	4.87 (0.36)
health adults 2017 kidney disease count of households persons	1.63 (0.20)	1.63 (0.20)
health adults 2017 liver disease count of households persons	1.29 (0.09)	1.29 (0.09)
health adults 2017 arthritis diagnosis count of households persons	18.70 (1.97)	18.69 (1.96)
health adults 2017 coronary count of households persons	5.12 (0.71)	5.12 (0.70)
health adults 2017 chronic joint symptoms count of households persons	24.00 (1.81)	23.99 (1.81)
health adults 2017 migraines or severe headaches count of households persons	11.62 (0.48)	11.62 (0.48)
health adults 2017 pain in neck count of households persons	12.66 (0.65)	12.66 (0.65)
health adults 2017 pain in lower back count of households persons	23.51 (1.27)	23.51 (1.27)
health adults 2017 pain in face or jaw count of households persons	3.27 (0.16)	3.27 (0.16)
health adults 2017 hearing trouble count of households persons	12.58 (1.32)	12.58 (1.31)

health adults 2017 vision trouble count of households persons	7.48 (0.50)	7.48 (0.50)
health adults 2017 absence of all natural teeth count of households persons	5.95 (0.73)	5.95 (0.73)
health adults 2017 sadness all or most of the time count of households persons	2.23 (0.23)	2.23 (0.23)
health adults 2017 sadness some of the time count of households persons	6.43 (0.44)	6.43 (0.44)
health adults 2017 hypertension count of households persons	22.03 (2.27)	22.03 (2.27)
health adults 2017 hopelessness all or most of the time count of households persons	1.59 (0.16)	1.59 (0.16)
health adults 2017 hopelessness some of the time count of households persons	3.79 (0.30)	3.79 (0.30)
health adults 2017 worthlessness all or most of the time count of households persons	1.35 (0.16)	1.35 (0.16)
health adults 2017 worthlessness some of the time count of households persons	2.90 (0.24)	2.90 (0.24)
health adults 2017 everything is an effort all or most of the time count of households persons	5.04 (0.41)	5.04 (0.41)
health adults 2017 everything is an effort some of the time count of households persons	7.70 (0.39)	7.70 (0.39)
health adults 2017 nervousness all or most of the time count of households persons	3.89 (0.25)	3.89 (0.25)
health adults 2017 nervousness some of the time count of households persons	9.91 (0.43)	9.91 (0.43)
health adults 2017 restlessness all of most of the time count of households persons	4.98 (0.34)	4.98 (0.34)
health adults 2017 restlessness some of the time count of households persons	9.82 (0.48)	9.82 (0.48)
health adults 2017 stroke count of households persons	2.20 (0.27)	2.20 (0.27)
health adults 2017 difficult of cannot be done any physical activity count of households persons	13.17 (1.29)	13.16 (1.29)
health adults 2017 difficult of cannot be done walk a quarter of a mile count of households persons	6.01 (0.66)	6.01 (0.66)
health adults 2017 difficult of cannot be done climb up 10 steps without resting count of households persons	4.38 (0.50)	4.38 (0.50)
health adults 2017 difficult of cannot be done stand for 2 hours count of households persons	8.21 (0.87)	8.21 (0.87)
health adults 2017 difficult of cannot be done sit for 2 hours count of households persons	2.73 (0.26)	2.73 (0.26)
health adults 2017 difficult of cannot be done stoop bend or kneel count of households persons	7.68 (0.80)	7.67 (0.79)
health adults 2017 difficult of cannot be done reach overhead count of households persons	2.05 (0.22)	2.05 (0.22)
health adults 2017 difficult of cannot be done grasp or handle small objects count of households persons	1.56 (0.17)	1.56 (0.17)
health adults 2017 difficult of cannot be done lift or carry 10 pounds count of households persons	3.61 (0.38)	3.61 (0.38)

health adults 2017 difficult of cannot be done push or pull large objects count of households persons	5.22 (0.53)	5.22 (0.53)
health adults 2017 emphysema count of households persons	1.21 (0.16)	1.21 (0.16)
health adults 2017 health status excellent or very good count of households persons	46.63 (2.34)	46.63 (2.34)
health adults 2017 health status good count of households persons	21.01 (1.19)	21.01 (1.19)
health adults 2017 health status fair or poor count of households persons	10.19 (0.95)	10.19 (0.95)
health adults 2017 ever had asthma count of households persons	9.88 (0.48)	9.88 (0.48)
health adults 2017 all current smokers count of households persons	11.89 (0.84)	11.89 (0.84)
health adults 2017 every day smokers count of households persons	9.06 (0.71)	9.06 (0.71)
health adults 2017 some day smokers count of households persons	2.82 (0.14)	2.82 (0.14)
health adults 2017 former smokers count of households persons	17.57 (1.42)	17.57 (1.42)
health adults 2017 non smokers count of households persons	48.48 (2.11)	48.49 (2.11)
health adults 2017 alcohol lifetime abstainer count of households persons	15.51 (0.88)	15.52 (0.88)
health adults 2017 alcohol former infrequent count of households persons	6.76 (0.53)	6.75 (0.53)
health adults 2017 alcohol former regular count of households persons	4.45 (0.36)	4.45 (0.36)
health adults 2017 still has asthma count of households persons	5.97 (0.30)	5.97 (0.30)
health adults 2017 alcohol current infrequent count of households persons	10.18 (0.46)	10.18 (0.46)
health adults 2017 alcohol current regular count of households persons	39.84 (2.05)	39.84 (2.05)
health adults 2017 body mass index underweight count of households persons	1.41 (0.06)	1.41 (0.06)
health adults 2017 hay fever count of households persons	6.49 (0.35)	6.49 (0.35)
health adults 2017 body mass index healthy weight count of households persons	25.77 (1.27)	25.77 (1.27)
health adults 2017 body mass index overweight count of households persons	25.45 (1.20)	25.45 (1.20)
health adults 2017 body mass index obese count of households persons	22.63 (1.44)	22.63 (1.44)
health adults 2017 all persons without a usual place of health care count of households persons	10.43 (0.56)	10.43 (0.56)
health adults 2017 all persons with a usual place of health care count of households persons	66.76 (3.15)	66.76 (3.15)
health adults 2017 type doctor s office or hmo count of households persons	48.81 (2.49)	48.81 (2.49)

health adults 2017 type clinic or health center count of households persons	15.26 (0.87)	15.26 (0.87)
health adults 2017 type some other place count of households persons	0.71 (0.04)	0.71 (0.04)
health adults 2017 office visits in the past 12 months none count of households persons	13.49 (0.63)	13.49 (0.63)
health adults 2017 sinusitis count of households persons	9.55 (0.53)	9.55 (0.53)
health adults 2017 office visits in the past 12 months 1 count of households persons	13.23 (0.57)	13.23 (0.57)
health adults 2017 office visits in the past 12 months 2 3 count of households persons	20.45 (0.97)	20.45 (0.97)
health adults 2017 office visits in the past 12 months 4 9 count of households persons	18.51 (1.12)	18.51 (1.12)
health adults 2017 office visits in the past 12 months 10 or more count of households persons	10.87 (0.66)	10.87 (0.66)
health adults 2017 last doctor visit 6 months or less count of households persons	53.62 (2.77)	53.62 (2.76)
health adults 2017 last doctor visit more than six months but less than 1 year count of households persons	10.63 (0.46)	10.63 (0.46)
health adults 2017 last doctor visit more than 1 year but not more than 2 years ago count of households persons	5.86 (0.30)	5.86 (0.30)
health adults 2017 last doctor visit more than 2 years but less than 5 years ago count of households persons	3.49 (0.16)	3.49 (0.16)
health adults 2017 last doctor visit more than 5 years excluding never count of households persons	2.14 (0.11)	2.13 (0.11)
health adults 2017 last doctor visit never count of households persons	0.79 (0.06)	0.79 (0.06)
health children 2017 number school days missed in past 12 months due to illness or injury aged 5 17 none count of households persons	26.59 (1.50)	26.60 (1.51)
health children 2017 number school days missed in past 12 months due to illness or injury aged 5 17 1 2 days count of households persons	29.22 (0.30)	29.22 (0.30)
health children 2017 number school days missed in past 12 months due to illness or injury aged 5 17 3 5 days count of households persons	24.24 (0.10)	24.24 (0.10)
health children 2017 number school days missed in past 12 months due to illness or injury aged 5 17 6 10 days count of households persons	9.63 (0.12)	9.63 (0.12)
health children 2017 number school days missed in past 12 months due to illness or injury aged 5 17 11 or more days count of households persons	4.09 (0.11)	4.09 (0.11)
health children 2017 number school days missed in past 12 months due to illness or injury aged 5 17 did not go to school count of households persons	0.38 (0.53)	0.38 (0.53)
health children 2017 emergency room visits in past 12 months for children under 18 none count of households persons	82.62 (0.51)	82.62 (0.51)
health children 2017 emergency room visits in past 12 months for children under 18 one count of households persons	11.50 (0.27)	11.50 (0.27)
health children 2017 emergency room visits in past 12 months for children under 18 two or more count of households persons	5.36 (0.31)	5.36 (0.31)
health children 2017 children receiving special education or early intervention services count of households persons	7.85 (0.08)	7.85 (0.08)
health children 2017 ever told had asthma count of households persons	12.95 (0.28)	12.96 (0.28)

health children 2017 still have asthma count of households persons	8.34 (0.36)	8.34 (0.36)
health children 2017 hay fever count of households persons	8.40 (0.15)	8.40 (0.15)
health children 2017 respiratory allergies count of households persons	10.11 (0.14)	10.11 (0.14)
health children 2017 food allergies count of households persons	5.77 (0.10)	5.77 (0.10)
health children 2017 skin allergies count of households persons	11.91 (0.39)	11.91 (0.39)
health children 2017 children 3 to 17 learning disability count of households persons	7.26 (0.15)	7.26 (0.15)
health children 2017 children 3 to 17 attention deficit hyperactivity disorder count of households persons	9.50 (0.19)	9.50 (0.19)
health children 2017 prescription medication taken regularly for at least 3 months count of households persons	13.19 (0.22)	13.19 (0.22)
health children 2017 excellent health status respondent assessed count of households persons	58.07 (1.25)	58.07 (1.25)
health children 2017 very good health status respondent assessed count of households persons	25.23 (0.20)	25.23 (0.20)
health children 2017 good health status respondent assessed count of households persons	13.36 (1.02)	13.35 (1.02)
health children 2017 fair of poor health status respondent assessed count of households persons	1.73 (0.16)	1.73 (0.16)
health children 2017 all persons without a usual place of health care count of households persons	3.95 (0.20)	3.95 (0.20)
health children 2017 all persons with a usual place of health care count of households persons	95.94 (0.20)	95.94 (0.20)
health children 2017 all persons with a usual place of health care clinic count of households persons	23.95 (1.96)	23.94 (1.96)
health children 2017 all persons with a usual place of health care doctor s office count of households persons	70.27 (2.42)	70.28 (2.43)
health children 2017 all persons with a usual place of health care emergency room count of households persons	0.43 (0.07)	0.43 (0.07)
health children 2017 all persons with a usual place of health care hospital outpatient count of households persons	0.72 (0.09)	0.72 (0.09)
health children 2017 all persons with a usual place of health care some other place count of households persons	0.29 (0.08)	0.29 (0.08)
health children 2017 last health care professional visit 6 months or less count of households persons	76.36 (0.20)	76.36 (0.20)
health children 2017 last health care professional visit more than six months but less than 1 year count of households persons	15.76 (0.12)	15.76 (0.12)
health children 2017 last health care professional visit more than 1 year but not more than 2 years ago count of households persons	4.34 (0.10)	4.34 (0.10)
health children 2017 last health care professional visit more than 2 years but less than 5 years ago count of households persons	0.94 (0.04)	0.94 (0.04)
health children 2017 last health care professional visit more than 5 years count of households persons	1.69 (0.11)	1.69 (0.11)
health children 2017 uninsured for health care count of households persons	29.46 (49.34)	29.71 (49.40)

health children 2017 unmet medical need count of households persons	1.39 (0.10)	1.39 (0.10)
health children 2017 delayed care due to cost count of households persons	2.36 (0.12)	2.36 (0.12)
health children 2017 children 2 17 years yes unmet dental need count of households persons	4.12 (0.21)	4.12 (0.21)
health children 2017 children 2 17 years no unmet dental need count of households persons	94.95 (0.15)	94.95 (0.15)
health children 2017 children 2 17 years less than 6 months since last dental visit count of households persons	66.87 (0.45)	66.87 (0.45)
health children 2017 children 2 17 years more than 6 months but less than 1 year since last dental visit count of households persons	16.59 (0.32)	16.59 (0.32)
health children 2017 children 2 17 years more than 1 year but not more than 2 years since last dental visit count of households persons	5.34 (0.19)	5.34 (0.19)
health children 2017 children 2 17 years more than 2 years but not more than 5 years ago since last dental visit count of households persons	1.95 (0.08)	1.95 (0.08)
health children 2017 children 2 17 years more than 5 years since last dental visit count of households persons	7.67 (0.40)	7.67 (0.41)

HH=Household
Fam=Family
Pop=Population
Non Fam=Non family
OT=Other
ER=Emergency room
RV=recreational vehicle
Equip=equipment
Misc.=miscellaneous
BCBS=Blue Cross Blue Shield
OOT=Out of town
RIHC=resource intensive healthcare
