# Supplementary material for: Can diverse population characteristics be leveraged in a machine learning pipeline to predict resource intensive healthcare utilization among hospital service areas?
Source: BMC Health Serv Res. 2022 Jun 30;22:847. doi: 10.1186/s12913-022-08154-4 (PMC9248096; doi:10.1186/s12913-022-08154-4)
Supplement: Supplementary file 5 — Additional file 5. [file 12913_2022_8154_MOESM5_ESM.pdf]

Additional File 5. Descriptive Statistics for Adult & Child Disease/Health Candidate Predictors (second order terms)

- Additional File 5
  - File format: PDF
  - File title: Descriptive Statistics for Adult & Child Disease/Health Candidate Predictors (second order terms)
  - File description: Long table with univariate results for second order terms

ER Visits (mean(SD))	
health adults 2017 prostate cancer count of households persons health children 2017 uninsured for health care count of households persons	24.99 (38.10)
health adults 2017 kidney disease count of households persons health children 2017 uninsured for health care count of households persons	45.38 (70.05)
health adults 2017 coronary count of households persons health children 2017 uninsured for health care count of households persons	141.69 (217.86)
health adults 2017 emphysema count of households persons health children 2017 uninsured for health care count of households persons	33.59 (51.59)
health adults 2017 last doctor visit more than six months but less than 1 year count of households persons health children 2017 uninsured for health care count of households persons	311.00 (514.07)
health adults 2017 last doctor visit more than 1 year but not more than 2 years ago count of households persons health children 2017 uninsured for health care count of households persons	172.52 (288.08)
health children 2017 number school days missed in past 12 months due to illness or injury aged 5 17 none count of households persons health children 2017 uninsured for health care count of households persons	787.07 (1320.29)
health children 2017 number school days missed in past 12 months due to illness or injury aged 5 17 6 10 days count of households persons health children 2017 uninsured for health care count of households persons	283.41 (473.53)
health children 2017 number school days missed in past 12 months due to illness or injury aged 5 17 11 or more days count of households persons health children 2017 uninsured for health care count of households persons	119.82 (198.80)
health children 2017 number school days missed in past 12 months due to illness or injury aged 5 17 did not go to school count of households persons health children 2017 uninsured for health care count of households persons	3.13 (0.36)
health children 2017 still have asthma count of households persons health children 2017 uninsured for health care count of households persons	246.11 (410.35)
health children 2017 skin allergies count of households persons health children 2017 uninsured for health care count of households persons	351.76 (588.98)
health children 2017 last health care professional visit more than 2 years but less than 5 years ago count of households persons health children 2017 uninsured for health care count of households persons	27.63 (45.76)
health children 2017 delayed care due to cost count of households persons health children 2017 uninsured for health care count of households persons	68.70 (112.69)
Inpatient Days (mean(SD))	
health adults 2017 prostate cancer count of households persons health children 2017 uninsured for health care count of households persons	25.21 (38.20)

health adults 2017 kidney disease count of households persons health children 2017 uninsured for health care count of households persons	45.76 (70.19)
health adults 2017 coronary count of households persons health children 2017 uninsured for health care count of households persons	142.91 (218.34)
health adults 2017 emphysema count of households persons health children 2017 uninsured for health care count of households persons	33.87 (51.69)
health adults 2017 last doctor visit more than six months but less than 1 year count of households persons health children 2017 uninsured for health care count of households persons	313.77 (514.91)
health adults 2017 last doctor visit more than 1 year but not more than 2 years ago count of households persons health children 2017 uninsured for health care count of households persons	174.06 (288.52)
health children 2017 number school days missed in past 12 months due to illness or injury aged 5 17 none count of households persons health children 2017 uninsured for health care count of households persons	794.12 (1322.01)
health children 2017 number school days missed in past 12 months due to illness or injury aged 5 17 1 2 days count of households persons health children 2017 uninsured for health care count of households persons	870.44 (1453.82)
health children 2017 number school days missed in past 12 months due to illness or injury aged 5 17 6 10 days count of households persons health children 2017 uninsured for health care count of households persons	285.82 (474.06)
health children 2017 number school days missed in past 12 months due to illness or injury aged 5 17 11 or more days count of households persons health children 2017 uninsured for health care count of households persons	120.83 (199.02)
health children 2017 number school days missed in past 12 months due to illness or injury aged 5 17 did not go to school count of households persons health children 2017 uninsured for health care count of households persons	3.13 (0.36)
health children 2017 still have asthma count of households persons health children 2017 uninsured for health care count of households persons	248.29 (410.88)
health children 2017 skin allergies count of households persons health children 2017 uninsured for health care count of households persons	354.85 (589.71)
health children 2017 last health care professional visit more than 2 years but less than 5 years ago count of households persons health children 2017 uninsured for health care count of households persons	27.86 (45.81)
health children 2017 delayed care due to cost count of households persons health children 2017 uninsured for health care count of households persons	69.26 (112.81)
Hospital Expenditures (mean(SD))	
health adults 2017 prostate cancer count of households persons health children 2017 uninsured for health care count of households persons	25.21 (38.20)
health adults 2017 kidney disease count of households persons health children 2017 uninsured for health care count of households persons	45.76 (70.19)
health adults 2017 coronary count of households persons health children 2017 uninsured for health care count of households persons	142.91 (218.34)
health adults 2017 emphysema count of households persons health children 2017 uninsured for health care count of households persons	33.87 (51.69)
health adults 2017 last doctor visit more than six months but less than 1 year count of households persons health children 2017 uninsured for health care count of households persons	313.77 (514.91)
health adults 2017 last doctor visit more than 1 year but not more than 2 years ago count of households persons health children 2017 uninsured for health care count of households persons	174.06 (288.52)
health children 2017 number school days missed in past 12 months due to illness or injury aged 5 17 none count of households persons health children 2017 uninsured for health care count of households persons	794.12 (1322.01)

health children 2017 number school days missed in past 12 months due to illness or injury aged 5 17 1 2 days count of households persons health children 2017 uninsured for health care count of households persons	870.44 (1453.82)
health children 2017 number school days missed in past 12 months due to illness or injury aged 5 17 6 10 days count of households persons health children 2017 uninsured for health care count of households persons	285.82 (474.06)
health children 2017 number school days missed in past 12 months due to illness or injury aged 5 17 11 or more days count of households persons health children 2017 uninsured for health care count of households persons	120.83 (199.02)
health children 2017 number school days missed in past 12 months due to illness or injury aged 5 17 did not go to school count of households persons health children 2017 uninsured for health care count of households persons	3.13 (0.36)
health children 2017 still have asthma count of households persons health children 2017 uninsured for health care count of households persons	248.29 (410.88)
health children 2017 skin allergies count of households persons health children 2017 uninsured for health care count of households persons	354.85 (589.71)
health children 2017 last health care professional visit more than 2 years but less than 5 years ago count of households persons health children 2017 uninsured for health care count of households persons	27.86 (45.81)
health children 2017 delayed care due to cost count of households persons health children 2017 uninsured for health care count of households persons	69.26 (112.81)

HH=Household
Fam=Family
Pop=Population
Non Fam=Non family
OT=Other
ER=Emergency room
RV=recreational vehicle
Equip=equipment
Misc.=miscellaneous
BCBS=Blue Cross Blue Shield
OOT=Out of town
RIHC=resource intensive healthcare
