# Supplementary material for: Can diverse population characteristics be leveraged in a machine learning pipeline to predict resource intensive healthcare utilization among hospital service areas?
Source: BMC Health Serv Res. 2022 Jun 30;22:847. doi: 10.1186/s12913-022-08154-4 (PMC9248096; doi:10.1186/s12913-022-08154-4)
Supplement: Supplementary file 6 — Additional file 6. [file 12913_2022_8154_MOESM6_ESM.pdf]

Additional File 6. Descriptive Statistics for Community Characteristics (Main Effects)

- Additional File 6
  - File format: PDF
  - File title: Descriptive Statistics for Community Characteristics (Main Effects)
  - File description: Long table with univariate results for main effects

	ER Visist	Inpatient Days & Hospital Expenditures
n	3153	3174
hsanum	26296.63 (14813.77)	26281.36 (14809.53)
census employment 2017 employment car truck van to work empl 16 persons	46.10 (7.08)	46.13 (7.08)
census employment 2017 employment car truck van to work alone empl 16 persons	40.56 (6.74)	40.59 (6.74)
census employment 2017 employment car truck van to work carpool empl 16 persons	5.54 (1.74)	5.54 (1.74)
census employment 2017 employment public transportation to work empl 16 persons	0.94 (2.40)	0.94 (2.39)
census employment 2017 employment bus or trolley bus to work empl 16 persons	0.60 (1.22)	0.60 (1.22)
census employment 2017 employment streetcar or trolley car to work empl 16 persons	0.01 (0.06)	0.01 (0.06)
census employment 2017 employment subway or elevated to work empl 16 persons	0.16 (1.24)	0.16 (1.24)
census employment 2017 employment railroad to work empl 16 persons	0.16 (0.72)	0.16 (0.73)
census employment 2017 employment ferry to work empl 16 persons	0.01 (0.09)	0.01 (0.09)
census employment 2017 employment taxi to work empl 16 persons	0.05 (0.16)	0.05 (0.16)
census employment 2017 employment motorcycle to work empl 16 persons	0.12 (0.16)	0.12 (0.16)
census employment 2017 employment bicycle to work empl 16 persons	0.27 (0.50)	0.27 (0.50)
census employment 2017 employment walked to work empl 16 persons	1.81 (1.69)	1.80 (1.69)

census employment 2017 employment other transportation to work empl 16 persons	0.50 (0.60)	0.50 (0.60)
census employment 2017 employment work at home empl 16 persons	2.41 (1.46)	2.41 (1.45)
census employment 2017 employment travel time less than 15 min empl 16 persons	20.31 (8.57)	20.28 (8.55)
census employment 2017 employment travel time 15 29 min empl 16 persons	14.71 (5.07)	14.73 (5.08)
census employment 2017 employment travel time 30 59 min empl 16 persons	11.00 (5.29)	11.02 (5.29)
census employment 2017 employment travel time 60 89 min empl 16 persons	2.48 (1.83)	2.48 (1.83)
census employment 2017 employment travel time 90 min empl 16 persons	1.30 (0.92)	1.30 (0.92)
census employment 2017 employment potential pop 16 persons	98.38 (0.33)	98.38 (0.33)
census employment 2017 employment civilian males pop 16 persons	27.46 (4.50)	27.47 (4.50)
census employment 2017 employment civilian females pop 16 persons	24.74 (3.70)	24.75 (3.70)
census employment 2017 employment armed forces male pop 16 persons	0.27 (1.20)	0.27 (1.20)
census employment 2017 employment armed forces female pop 16 persons	0.04 (0.17)	0.04 (0.17)
census employment 2017 employment unemployed males pop 16 persons	1.38 (0.38)	1.38 (0.38)
census employment 2017 employment unemployed female pop 16 persons	1.24 (0.34)	1.24 (0.34)
census employment 2017 employment not in the labor force male pop 16 persons	19.24 (5.10)	19.23 (5.09)
census employment 2017 employment not in the labor force female pop 16 persons	24.00 (3.81)	24.00 (3.81)
census employment 2017 employment civilian total pop 16 persons	52.20 (7.59)	52.22 (7.58)
census employment 2017 employment agriculture forestry fishing and hunting pop 16 persons	1.82 (2.54)	1.81 (2.54)
census employment 2017 employment mining quarrying and oil and gas extraction pop 16 persons	0.70 (1.76)	0.69 (1.76)
census employment 2017 employment construction pop 16 persons	3.45 (1.29)	3.45 (1.29)
census employment 2017 employment manufacturing pop 16 persons	6.16 (3.70)	6.16 (3.70)
census employment 2017 employment wholesale trade pop 16 persons	1.31 (0.68)	1.31 (0.68)
census employment 2017 employment retail trade pop 16 persons	6.12 (1.48)	6.12 (1.48)
census employment 2017 employment transportation and warehousing pop 16 persons	2.06 (0.99)	2.07 (0.99)
census employment 2017 employment utilities pop 16 persons	0.62 (0.57)	0.62 (0.56)

census employment 2017 employment information pop 16 persons	0.89 (0.56)	0.89 (0.57)
census employment 2017 employment finance and insurance pop 16 persons	1.96 (1.13)	1.96 (1.13)
census employment 2017 employment real estate and rental and leasing pop 16 persons	0.76 (0.49)	0.76 (0.49)
census employment 2017 employment professional scientific and technical services pop 16 persons	2.27 (1.90)	2.28 (1.91)
census employment 2017 employment management of companies and enterprises pop 16 persons	0.03 (0.06)	0.03 (0.06)
census employment 2017 employment administrative and support and waste mgt services pop 16 persons	1.76 (0.84)	1.77 (0.84)
census employment 2017 employment educational services pop 16 persons	4.97 (2.13)	4.97 (2.13)
census employment 2017 employment health care and social assistance pop 16 persons	7.45 (1.95)	7.45 (1.95)
census employment 2017 employment arts entertainment and recreation pop 16 persons	1.00 (0.85)	0.99 (0.85)
census employment 2017 employment accommodation and food services pop 16 persons	3.58 (1.56)	3.58 (1.55)
census employment 2017 employment other services pop 16 persons	2.54 (0.77)	2.54 (0.77)
census employment 2017 employment public administration pop 16 persons	2.75 (1.52)	2.75 (1.52)
census employment 2017 occupation management business and financial operations pop 16 persons	6.77 (2.81)	6.78 (2.82)
census employment 2017 occupation professional and related pop 16 persons	10.18 (3.39)	10.20 (3.40)
census employment 2017 occupation sales and office pop 16 persons	11.80 (2.49)	11.81 (2.48)
census employment 2017 occupation service pop 16 persons	9.88 (2.15)	9.88 (2.15)
census employment 2017 occupation farming fishing and forestry pop 16 persons	0.82 (1.27)	0.82 (1.27)
census employment 2017 occupation construction extraction and maintenance pop 16 persons	5.07 (1.88)	5.07 (1.88)
census employment 2017 occupation production transportation and material moving pop 16 persons	7.68 (3.14)	7.68 (3.14)
census employment 2017 employment white collar pop 16 persons	28.75 (7.20)	28.78 (7.22)
census employment 2017 employment blue collar pop 16 persons	12.75 (3.94)	12.74 (3.93)
census employment 2017 employment private for profit wage and salary workers employee pop 16 persons	33.27 (6.89)	33.30 (6.89)
census employment 2017 employment private for profit wage and salary workers self pop 16 persons	1.85 (0.99)	1.86 (0.99)
census employment 2017 employment private not for profit wage and salary workers pop 16 persons	4.33 (1.95)	4.33 (1.95)
census employment 2017 employment local government workers pop 16 persons	4.41 (1.77)	4.41 (1.76)

census employment 2017 employment state government workers pop 16 persons	3.01 (1.95)	3.01 (1.95)
census employment 2017 employment federal government workers pop 16 persons	1.34 (1.43)	1.34 (1.43)
census employment 2017 employment self employed workers in own not incorporated business pop 16 persons	3.87 (1.96)	3.86 (1.95)
census employment 2017 employment unpaid family workers pop 16 persons	0.12 (0.19)	0.12 (0.19)
census housing units 2017 housing median year built count year	423.46 (447.00)	424.81 (447.74)
census housing units 2017 housing median year moved in count year	429.81 (454.16)	431.17 (454.91)
census housing units 2017 housing median rent count	117.66 (136.53)	118.43 (137.03)
census housing units 2017 housing median value owner households count	31401.50 (47291.45)	31612.80 (47387.75)
census housing units 2017 home heating fuel utility gas count housing units	37.62 (22.88)	37.70 (22.88)
census housing units 2017 home heating fuel bottled tank or lp gas count housing units	7.52 (6.94)	7.49 (6.93)
census housing units 2017 home heating fuel electricity count housing units	30.68 (20.34)	30.66 (20.32)
census housing units 2017 home heating fuel fuel oil kerosene etc count housing units	5.07 (11.00)	5.09 (11.03)
census housing units 2017 home heating fuel coal or coke count housing units	0.16 (0.70)	0.16 (0.70)
census housing units 2017 home heating fuel wood count housing units	3.51 (5.01)	3.49 (5.00)
census housing units 2017 home heating fuel solar energy count housing units	0.04 (0.13)	0.04 (0.13)
census housing units 2017 home heating fuel other fuel count housing units	0.56 (0.74)	0.56 (0.74)
census housing units 2017 home heating fuel no fuel used count housing units	0.61 (3.55)	0.61 (3.54)
census housing units 2017 housing occupied units count housing units	85.76 (9.22)	85.80 (9.21)
census housing units 2017 housing vacant units count housing units	14.24 (9.22)	14.20 (9.21)
census housing units 2017 housing vacant units for rent count housing units	2.90 (1.25)	2.90 (1.25)
census housing units 2017 housing vacant units rented not occupied count housing units	0.17 (0.11)	0.17 (0.11)
census housing units 2017 housing vacant units for sale count housing units	1.43 (0.57)	1.43 (0.57)

census housing units 2017 housing vacant units sold not occupied count housing units	0.42 (0.32)	0.42 (0.32)
census housing units 2017 housing vacant units seasonal recreational or occasional use count housing units	5.14 (8.36)	5.12 (8.34)
census housing units 2017 housing vacant units for migrant workers count housing units	0.04 (0.13)	0.04 (0.13)
census housing units 2017 housing vacant units vacant other count housing units	4.14 (2.89)	4.13 (2.89)
census housing units 2017 housing structure with 1 unit detached count housing units	58.67 (10.59)	58.67 (10.60)
census housing units 2017 housing structure with 1 unit attached count housing units	3.16 (4.04)	3.18 (4.05)
census housing units 2017 housing structure with 2 units count housing units	3.01 (2.76)	3.02 (2.76)
census housing units 2017 housing structure with 3 4 units count housing units	3.37 (2.31)	3.38 (2.33)
census housing units 2017 housing structure with 5 9 units count housing units	3.20 (2.19)	3.21 (2.19)
census housing units 2017 housing structure with 10 19 units count housing units	2.46 (2.35)	2.47 (2.36)
census housing units 2017 housing structure with 20 49 units count housing units	1.91 (2.03)	1.91 (2.04)
census housing units 2017 housing structure with 50 units count housing units	1.86 (2.94)	1.87 (2.93)
census housing units 2017 housing structure mobile home count housing units	8.02 (6.65)	8.01 (6.66)
census housing units 2017 housing structure boat rv van other count housing units	0.08 (0.22)	0.08 (0.22)
census housing units 2017 housing rent less than 250 count housing units	2.90 (2.37)	2.90 (2.37)
census housing units 2017 housing rent 250 499 count housing units	7.74 (4.94)	7.72 (4.93)
census housing units 2017 housing rent 500 749 count housing units	7.46 (4.80)	7.47 (4.81)
census housing units 2017 housing rent 750 999 count housing units	3.44 (3.63)	3.46 (3.64)
census housing units 2017 housing rent 1000 1249 count housing units	1.89 (2.80)	1.90 (2.81)
census housing units 2017 housing rent 1250 1499 count housing units	1.14 (2.11)	1.15 (2.12)
census housing units 2017 housing rent 1500 1999 count housing units	0.90 (1.96)	0.91 (1.98)
census housing units 2017 housing rent 2000 count housing units	0.58 (1.52)	0.58 (1.53)
census housing units 2017 housing no cash rent count housing units	2.61 (1.67)	2.60 (1.67)
census housing units 2017 housing owner households valued less than 10000 count housing units	1.28 (1.09)	1.28 (1.10)
census housing units 2017 housing owner households valued 10000 14999 count housing units	0.92 (0.82)	0.92 (0.82)

census housing units 2017 housing owner households valued 15000 19999 count housing units	0.82 (0.78)	0.82 (0.78)
census housing units 2017 housing owner households valued 20000 24999 count housing units	0.90 (0.91)	0.90 (0.91)
census housing units 2017 housing owner households valued 25000 29999 count housing units	0.83 (0.87)	0.82 (0.87)
census housing units 2017 housing owner households valued 30000 34999 count housing units	1.01 (1.04)	1.01 (1.04)
census housing units 2017 housing owner households valued 35000 39999 count housing units	0.74 (0.80)	0.74 (0.80)
census housing units 2017 housing owner households valued 40000 49999 count housing units	1.90 (1.73)	1.89 (1.73)
census housing units 2017 housing owner households valued 50000 59999 count housing units	2.21 (1.82)	2.21 (1.83)
census housing units 2017 housing owner households valued 60000 69999 count housing units	2.46 (1.92)	2.45 (1.92)
census housing units 2017 housing owner households valued 70000 79999 count housing units	2.70 (1.94)	2.70 (1.94)
census housing units 2017 housing owner households valued 80000 89999 count housing units	2.98 (1.95)	2.97 (1.95)
census housing units 2017 housing owner households valued 90000 99999 count housing units	2.39 (1.56)	2.38 (1.56)
census housing units 2017 housing owner households valued 100000 124999 count housing units	5.47 (2.78)	5.46 (2.78)
census housing units 2017 housing owner households valued 125000 149999 count housing units	4.14 (2.33)	4.14 (2.33)
census housing units 2017 housing owner households valued 150000 174999 count housing units	5.09 (2.50)	5.09 (2.50)
census housing units 2017 housing owner households valued 175000 199999 count housing units	3.10 (1.91)	3.10 (1.92)
census housing units 2017 housing owner households valued 200000 249999 count housing units	4.87 (2.95)	4.87 (2.96)
census housing units 2017 housing owner households valued 250000 299999 count housing units	3.72 (2.84)	3.74 (2.86)
census housing units 2017 housing owner households valued 300000 399999 count housing units	4.05 (4.23)	4.07 (4.25)
census housing units 2017 housing owner households valued 400000 499999 count housing units	1.95 (2.82)	1.97 (2.85)
census housing units 2017 housing owner households valued 500000 749999 count housing units	2.08 (3.80)	2.10 (3.83)
census housing units 2017 housing owner households valued 750000 999999 count housing units	0.72 (1.77)	0.73 (1.78)
census housing units 2017 housing owner households valued more than 1000000 count housing units	0.75 (2.06)	0.75 (2.06)
census housing units 2017 housing built 2010 or later count housing units	9.12 (3.60)	9.11 (3.59)
census housing units 2017 housing built 2000 to 2009 count housing units	10.88 (6.58)	10.88 (6.58)
census housing units 2017 housing built 1990 to 1999 count housing units	12.38 (5.53)	12.37 (5.53)

census housing units 2017 housing built 1980 to 1989 count housing units	12.27 (5.07)	12.27 (5.07)
census housing units 2017 housing built 1970 to 1979 count housing units	15.37 (4.40)	15.37 (4.40)
census housing units 2017 housing built 1960 to 1969 count housing units	10.19 (3.77)	10.20 (3.78)
census housing units 2017 housing built 1950 to 1959 count housing units	10.03 (5.24)	10.05 (5.27)
census housing units 2017 housing built 1940 to 1949 count housing units	5.19 (2.91)	5.19 (2.92)
census housing units 2017 housing built 1939 or earlier count housing units	14.58 (11.32)	14.56 (11.33)
census housing units 2017 housing year moved in 2010 or later count housing units	37.94 (7.44)	37.96 (7.45)
census housing units 2017 housing year moved in 2000 to 2009 count housing units	22.06 (3.93)	22.06 (3.92)
census housing units 2017 housing year moved in 1990 to 1999 count housing units	11.78 (2.17)	11.79 (2.17)
census housing units 2017 housing year moved in 1980 to 1989 count housing units	6.33 (1.70)	6.33 (1.70)
census housing units 2017 housing year moved in 1970 to 1979 count housing units	4.77 (1.80)	4.77 (1.80)
census housing units 2017 housing year moved in 1969 or earlier count housing units	2.88 (1.47)	2.88 (1.48)
census housing units 2017 housing owner occupied count housing units	57.10 (8.75)	57.12 (8.76)
census housing units 2017 housing renter occupied count housing units	28.66 (8.69)	28.68 (8.71)
census housing units 2017 housing owner households with mortgage any count households	33.97 (9.21)	34.00 (9.22)
census housing units 2017 housing owner households with no mortgage count households	23.13 (7.11)	23.11 (7.12)

HH=Household
Fam=Family
Pop=Population
Non Fam=Non family
OT=Other
ER=Emergency room
RV=recreational vehicle
Equip=equipment
Misc.=miscellaneous
BCBS=Blue Cross Blue Shield
OOT=Out of town
RIHC=resource intensive healthcare
