# Supplementary material for: Can diverse population characteristics be leveraged in a machine learning pipeline to predict resource intensive healthcare utilization among hospital service areas?
Source: BMC Health Serv Res. 2022 Jun 30;22:847. doi: 10.1186/s12913-022-08154-4 (PMC9248096; doi:10.1186/s12913-022-08154-4)
Supplement: Supplementary file 7 — Additional file 7. [file 12913_2022_8154_MOESM7_ESM.pdf]

Additional File 7. Descriptive Statistics for Community Characteristics Candidate Predictors (second order terms)

- Additional File 7
  - File format: PDF
  - File title: Descriptive Statistics for Community Characteristics Candidate Predictors (second order terms)
  - File description: Long table with univariate results for second order terms

ER visits (mean(SD))	
census employment 2017 employment subway or elevated to work empl 16 persons census housing units 2017 housing median year built count year	42.18 (334.89)
census employment 2017 employment taxi to work empl 16 persons census housing units 2017 housing median year built count year	15.08 (83.51)
census employment 2017 employment travel time less than 15 min empl 16 persons census housing units 2017 housing median year built count year	9075.14 (11924.06)
census employment 2017 employment travel time 15 29 min empl 16 persons census housing units 2017 housing median year built count year	5525.01 (5886.52)
census housing units 2017 housing median year moved in count year census housing units 2017 housing median year built count year	384947.03 (892775.48)
census housing units 2017 housing median rent count census housing units 2017 housing median year built count year	99150.16 (251620.19)
census housing units 2017 housing median value owner households count census housing units 2017 housing median year built count year	26324942.93 (82379823.39)
census housing units 2017 home heating fuel utility gas count housing units census housing units 2017 housing median year built count year	15114.46 (20573.02)
census housing units 2017 home heating fuel bottled tank or lp gas count housing units census housing units 2017 housing median year built count year	3886.15 (6984.29)
census housing units 2017 home heating fuel coal or coke count housing units census housing units 2017 housing median year built count year	47.61 (243.16)
census housing units 2017 housing structure with 1 unit attached count housing units census housing units 2017 housing median year built count year	1029.20 (1893.05)
census housing units 2017 housing rent 250 499 count housing units census housing units 2017 housing median year built count year	3525.11 (4931.95)
census housing units 2017 housing year moved in 2010 or later count housing units census housing units 2017 housing median year built count year	15293.80 (16033.74)
census housing units 2017 housing year moved in 2000 to 2009 count housing units census housing units 2017 housing median year built count year	9135.93 (9758.09)
census housing units 2017 housing renter occupied count housing units census housing units 2017 housing median year built count year	11353.02 (12468.35)
Inpatient Days (mean(SD))	
census employment 2017 employment subway or elevated to work empl 16 persons census housing units 2017 housing median year built count year	42.16 (333.86)
census employment 2017 employment taxi to work empl 16 persons census housing units 2017 housing median year built count year	15.26 (83.62)

census employment 2017 employment walked to work empl 16 persons census housing units 2017 housing median year built count year	823.67 (1776.68)
census employment 2017 employment travel time less than 15 min empl 16 persons census housing units 2017 housing median year built count year	9077.06 (11898.16)
census employment 2017 employment travel time 15 29 min empl 16 persons census housing units 2017 housing median year built count year	5569.28 (5974.90)
census employment 2017 employment agriculture forestry fishing and hunting pop 16 persons census housing units 2017 housing median year built count year	1099.21 (2722.27)
census employment 2017 employment health care and social assistance pop 16 persons census housing units 2017 housing median year built count year	3116.20 (3640.09)
census employment 2017 occupation management business and financial operations pop 16 persons census housing units 2017 housing median year built count year	2837.98 (3568.27)
census employment 2017 employment self employed workers in own not incorporated business pop 16 persons census housing units 2017 housing median year built count year	1834.13 (2747.16)
census housing units 2017 housing median value owner households count census housing units 2017 housing median year built count year	26518156.05 (82499483.95)
census housing units 2017 housing median year moved in count year census housing units 2017 housing median year built count year	386775.46 (894670.02)
census housing units 2017 housing median rent count census housing units 2017 housing median year built count year	99910.52 (252381.00)
census housing units 2017 home heating fuel fuel oil kerosene etc count housing units census housing units 2017 housing median year built count year	1647.19 (5123.77)
census housing units 2017 housing vacant units vacant other count housing units census housing units 2017 housing median year built count year	2137.99 (3694.65)
census housing units 2017 housing year moved in 2000 to 2009 count housing units census housing units 2017 housing median year built count year	9168.37 (9777.28)
Hospital Expenditures (mean(SD))	
census employment 2017 employment subway or elevated to work empl 16 persons census housing units 2017 housing median year built count year	42.16 (333.86)
census employment 2017 employment taxi to work empl 16 persons census housing units 2017 housing median year built count year	15.26 (83.62)
census employment 2017 employment walked to work empl 16 persons census housing units 2017 housing median year built count year	823.67 (1776.68)
census employment 2017 employment travel time less than 15 min empl 16 persons census housing units 2017 housing median year built count year	9077.06 (11898.16)
census employment 2017 employment travel time 15 29 min empl 16 persons census housing units 2017 housing median year built count year	5569.28 (5974.90)
census employment 2017 employment agriculture forestry fishing and hunting pop 16 persons census housing units 2017 housing median year built count year	1099.21 (2722.27)
census employment 2017 employment health care and social assistance pop 16 persons census housing units 2017 housing median year built count year	3116.20 (3640.09)
census employment 2017 occupation management business and financial operations pop 16 persons census housing units 2017 housing median year built count year	2837.98 (3568.27)
census employment 2017 employment self employed workers in own not incorporated business pop 16 persons census housing units 2017 housing median year built count year	1834.13 (2747.16)

census housing units 2017 housing median value owner households count census housing units 2017 housing median year built count year	26518156.05 (82499483.95)
census housing units 2017 housing median year moved in count year census housing units 2017 housing median year built count year	386775.46 (894670.02)
census housing units 2017 housing median rent count census housing units 2017 housing median year built count year	99910.52 (252381.00)
census housing units 2017 home heating fuel fuel oil kerosene etc count housing units census housing units 2017 housing median year built count year	1647.19 (5123.77)
census housing units 2017 housing vacant units vacant other count housing units census housing units 2017 housing median year built count year	2137.99 (3694.65)
census housing units 2017 housing year moved in 2000 to 2009 count housing units census housing units 2017 housing median year built count year	9168.37 (9777.28)

HH=Household
Fam=Family
Pop=Population
Non Fam=Non family
OT=Other
ER=Emergency room
RV=recreational vehicle
Equip=equipment
Misc.=miscellaneous
BCBS=Blue Cross Blue Shield
OOT=Out of town
RIHC=resource intensive healthcare
