# Supplementary material for: Can diverse population characteristics be leveraged in a machine learning pipeline to predict resource intensive healthcare utilization among hospital service areas?
Source: BMC Health Serv Res. 2022 Jun 30;22:847. doi: 10.1186/s12913-022-08154-4 (PMC9248096; doi:10.1186/s12913-022-08154-4)
Supplement: Supplementary file 8 — Additional file 8. [file 12913_2022_8154_MOESM8_ESM.pdf]

Additional File 8. Descriptive Statistics for Consumer Expenditures (Main Effects)

- Additional File 8
  - File format: PDF
  - File title: Descriptive Statistics for Consumer Expenditures (Main Effects)
  - File description: Long table with univariate results for main effects

	ER Visits	Inpatient Days & Hospital Expenditures
n	3153	3174
expenditures food 2017 cola	29.12 (3.82)	29.10 (3.82)
expenditures food 2017 other carbonated drinks	28.20 (3.46)	28.19 (3.46)
expenditures food 2017 tea	12.69 (1.53)	12.69 (1.53)
expenditures food 2017 coffee	36.28 (5.55)	36.26 (5.55)
expenditures food 2017 noncarbonated fruit flavored drinks including non frozen lemonade	9.10 (1.01)	9.09 (1.01)
expenditures food 2017 bottled water	23.74 (2.15)	23.74 (2.15)
expenditures food 2017 sports drinks	6.84 (0.78)	6.84 (0.78)
expenditures food 2017 nonalcoholic beer	0.08 (0.02)	0.08 (0.02)
expenditures food 2017 food prepared by consumer unit on out of town trips	22.27 (4.09)	22.27 (4.09)
expenditures food 2017 food away from home	1252.36 (188.61)	1252.33 (188.94)
expenditures food 2017 meals at restaurants carry outs and other	1064.55 (152.36)	1064.49 (152.59)
expenditures food 2017 lunch	333.26 (45.28)	333.25 (45.35)
expenditures food 2017 lunch at fast food take out delivery concession stands buffet and cafeteria	158.62 (18.06)	158.61 (18.08)
expenditures food 2017 lunch at full service restaurants	141.57 (22.71)	141.56 (22.76)
expenditures food 2017 lunch at vending machines and mobile vendors	5.44 (0.66)	5.44 (0.66)
expenditures food 2017 lunch at employer and school cafeterias	27.52 (3.47)	27.52 (3.48)

expenditures food 2017 dinner	515.22 (78.77)	515.19 (78.90)
expenditures food 2017 dinner at fast food take out delivery concession stands buffet and cafeteria	180.83 (20.98)	180.79 (20.98)
expenditures food 2017 dinner at full service restaurants	348.38 (61.18)	348.26 (61.29)
expenditures food 2017 dinner at vending machines and mobile vendors	1.70 (0.21)	1.70 (0.21)
expenditures food 2017 dinner at employer and school cafeterias	2.43 (0.39)	2.43 (0.40)
expenditures food 2017 snacks and nonalcoholic beverages	95.26 (12.77)	95.23 (12.78)
expenditures food 2017 snacks and nonalcoholic beverages at fast food take out delivery concession stands buffet and cafeteria	60.98 (8.65)	60.96 (8.66)
expenditures food 2017 snacks and nonalcoholic beverages at full service restaurants	20.98 (2.77)	20.97 (2.77)
expenditures food 2017 snacks and nonalcoholic beverages at vending machines and mobile vendors	9.10 (1.13)	9.10 (1.13)
expenditures food 2017 snacks and nonalcoholic beverages at employer and school cafeterias	4.30 (0.53)	4.30 (0.53)
expenditures food 2017 breakfast and brunch	126.28 (16.66)	126.23 (16.67)
expenditures food 2017 breakfast and brunch at fast food take out delivery concession stands buffet and cafeteria	68.02 (8.49)	68.00 (8.49)
expenditures food 2017 breakfast and brunch at full service restaurants	49.52 (6.96)	49.50 (6.97)
expenditures food 2017 breakfast and brunch at vending machines and mobile vendors	3.09 (0.48)	3.09 (0.48)
expenditures food 2017 breakfast and brunch at employer and school cafeterias	5.43 (0.70)	5.43 (0.70)
expenditures food 2017 food or board at school	13.18 (1.94)	13.17 (1.95)
expenditures food 2017 catered affairs	15.78 (3.61)	15.77 (3.62)
expenditures food 2017 education	608.32 (106.05)	608.42 (106.22)
expenditures food 2017 college tuition	368.90 (70.13)	368.97 (70.22)
expenditures food 2017 elementary and high school tuition	87.30 (19.02)	87.34 (19.06)
expenditures food 2017 vocational and technical school tuition	2.87 (0.64)	2.87 (0.64)
expenditures food 2017 test preparation tutoring services	7.81 (2.33)	7.82 (2.33)
expenditures food 2017 other schools tuition	3.80 (0.86)	3.80 (0.86)
expenditures food 2017 other school expenses including rentals	19.80 (3.19)	19.80 (3.19)
expenditures food 2017 school books supplies equipment for college	19.07 (3.49)	19.06 (3.49)

expenditures food 2017 school books supplies equipment for elementary high school	7.10 (1.13)	7.09 (1.13)
expenditures food 2017 school books supplies equipment for vocational and technical schools	0.22 (0.05)	0.22 (0.05)
expenditures food 2017 school books supplies equipment for day care nursery	0.15 (0.03)	0.15 (0.03)
expenditures food 2017 eggs	25.91 (2.98)	25.90 (2.98)
expenditures food 2017 dairy products	171.52 (23.66)	171.42 (23.69)
expenditures food 2017 fresh milk and cream	58.33 (7.44)	58.30 (7.44)
expenditures food 2017 fresh milk all types	49.05 (6.08)	49.02 (6.09)
expenditures food 2017 cream	9.40 (1.45)	9.40 (1.45)
expenditures food 2017 other dairy products	113.37 (16.36)	113.30 (16.39)
expenditures food 2017 butter	10.62 (1.45)	10.62 (1.45)
expenditures food 2017 cheese	56.04 (8.68)	56.00 (8.69)
expenditures food 2017 ice cream and related products	22.02 (2.99)	22.01 (3.00)
expenditures food 2017 miscellaneous dairy products	24.62 (3.40)	24.61 (3.40)
expenditures food 2017 fruits and vegetables	321.57 (41.38)	321.45 (41.41)
expenditures food 2017 fresh fruits	119.42 (16.22)	119.37 (16.24)
expenditures food 2017 apples	17.55 (2.46)	17.54 (2.46)
expenditures food 2017 bananas	17.86 (2.24)	17.85 (2.24)
expenditures food 2017 oranges	12.61 (1.50)	12.61 (1.50)
expenditures food 2017 citrus fruits excluding oranges	18.54 (2.60)	18.54 (2.60)
expenditures food 2017 food on out of town trips	130.81 (22.17)	130.81 (22.19)
expenditures food 2017 school lunches	26.39 (4.47)	26.39 (4.49)
expenditures food 2017 meals as pay	11.30 (2.59)	11.30 (2.60)
expenditures food 2017 alcoholic beverages	218.21 (35.21)	218.19 (35.28)
expenditures food 2017 alcoholic beverages at home	121.31 (18.92)	121.30 (18.97)
expenditures food 2017 beer and ale	49.88 (8.38)	49.88 (8.41)

expenditures food 2017 whiskey	4.97 (0.80)	4.97 (0.81)
expenditures food 2017 wine	56.93 (9.95)	56.93 (9.96)
expenditures food 2017 other alcoholic beverages	9.93 (1.61)	9.92 (1.61)
expenditures food 2017 alcoholic beverages away from home	97.00 (16.76)	96.99 (16.78)
expenditures food 2017 beer and ale away	31.67 (5.39)	31.67 (5.40)
expenditures food 2017 wine away	18.96 (3.66)	18.96 (3.66)
expenditures food 2017 other alcoholic beverages away	20.28 (3.56)	20.28 (3.56)
expenditures food 2017 alcoholic beverages purchased on trips	25.76 (4.78)	25.76 (4.79)
expenditures food 2017 other beef	8.74 (1.27)	8.73 (1.27)
expenditures food 2017 pork	67.18 (8.19)	67.16 (8.19)
expenditures food 2017 bacon	15.53 (1.94)	15.52 (1.94)
expenditures food 2017 pork chops	10.08 (1.55)	10.08 (1.55)
expenditures food 2017 ham	12.73 (1.55)	12.72 (1.55)
expenditures food 2017 sausage	14.08 (1.71)	14.07 (1.71)
expenditures food 2017 other pork	14.74 (1.84)	14.74 (1.84)
expenditures food 2017 other meats	51.67 (6.36)	51.65 (6.36)
expenditures food 2017 frankfurters	9.37 (1.19)	9.37 (1.20)
expenditures food 2017 lunch meats cold cuts	35.97 (4.76)	35.95 (4.76)
expenditures food 2017 lamb organ meats and others	6.28 (0.93)	6.28 (0.93)
expenditures food 2017 poultry	71.17 (8.73)	71.15 (8.73)
expenditures food 2017 fresh and frozen chickens	57.45 (6.97)	57.43 (6.97)
expenditures food 2017 other poultry	13.62 (1.83)	13.62 (1.84)
expenditures food 2017 fish and seafood	52.83 (6.86)	52.83 (6.86)
expenditures food 2017 canned fish and seafood	7.90 (0.97)	7.89 (0.97)
expenditures food 2017 fresh fish and shellfish	26.68 (3.75)	26.68 (3.75)

expenditures food 2017 frozen fish and shellfish	18.30 (2.33)	18.29 (2.33)
expenditures food 2017 other fresh fruits	52.84 (6.95)	52.82 (6.96)
expenditures food 2017 fresh vegetables	103.99 (13.30)	103.95 (13.31)
expenditures food 2017 potatoes	16.04 (2.10)	16.04 (2.10)
expenditures food 2017 lettuce	13.72 (1.76)	13.72 (1.76)
expenditures food 2017 tomatoes	17.89 (2.33)	17.88 (2.33)
expenditures food 2017 other fresh vegetables	56.35 (7.61)	56.33 (7.61)
expenditures food 2017 processed fruits	44.53 (5.63)	44.52 (5.63)
expenditures food 2017 frozen fruits and fruit juices	5.39 (0.74)	5.39 (0.74)
expenditures food 2017 canned fruits	8.09 (1.07)	8.08 (1.07)
expenditures food 2017 dried fruit	3.35 (0.47)	3.35 (0.47)
expenditures food 2017 fresh fruit juice	5.66 (0.71)	5.66 (0.71)
expenditures food 2017 canned and bottled fruit juice	22.22 (3.09)	22.21 (3.09)
expenditures food 2017 processed vegetables	53.63 (6.90)	53.61 (6.90)
expenditures food 2017 frozen vegetables	14.25 (1.86)	14.24 (1.86)
expenditures food 2017 canned and dried vegetables and juices	39.39 (5.04)	39.38 (5.04)
expenditures food 2017 other food at home	585.99 (74.83)	585.75 (74.88)
expenditures food 2017 sugar and other sweets	64.01 (8.85)	63.99 (8.85)
expenditures food 2017 candy and chewing gum	43.17 (6.76)	43.15 (6.77)
expenditures food 2017 rice	11.06 (2.11)	11.07 (2.11)
expenditures food 2017 pasta cornmeal and other cereal products	15.67 (2.00)	15.66 (2.00)
expenditures food 2017 bakery products	142.20 (18.98)	142.13 (18.98)
expenditures food 2017 bread	42.09 (5.52)	42.07 (5.52)
expenditures food 2017 cookies and crackers	35.16 (4.71)	35.15 (4.71)
expenditures food 2017 frozen and refrigerated bakery products	11.35 (1.48)	11.35 (1.48)

expenditures food 2017 cakes and cupcakes	14.96 (1.85)	14.95 (1.85)
expenditures food 2017 bread and cracker products	2.39 (0.34)	2.38 (0.34)
expenditures food 2017 sweetrolls coffee cakes doughnuts	9.18 (1.15)	9.17 (1.15)
expenditures food 2017 pies tarts turnovers	6.43 (0.91)	6.42 (0.92)
expenditures food 2017 meats poultry fish and eggs	369.33 (44.43)	369.21 (44.43)
expenditures food 2017 beef	100.43 (12.86)	100.39 (12.87)
expenditures food 2017 ground beef	43.20 (5.52)	43.17 (5.52)
expenditures food 2017 roast	14.84 (1.98)	14.83 (1.98)
expenditures food 2017 steak	34.38 (4.94)	34.37 (4.94)
expenditures food 2017 food	2952.02 (379.16)	2951.06 (379.41)
expenditures food 2017 food at home	1665.24 (215.51)	1664.64 (215.57)
expenditures food 2017 cereals and bakery products	215.16 (28.02)	215.09 (28.03)
expenditures food 2017 cereals and cereal products	73.19 (9.52)	73.17 (9.53)
expenditures food 2017 flour	3.75 (0.50)	3.75 (0.50)
expenditures food 2017 prepared flour mixes	5.38 (0.79)	5.38 (0.79)
expenditures food 2017 ready to eat and cooked cereals	37.05 (4.95)	37.03 (4.95)
expenditures food 2017 sugar	7.45 (1.21)	7.45 (1.21)
expenditures food 2017 artificial sweeteners	1.74 (0.28)	1.74 (0.28)
expenditures food 2017 jams preserves other sweets	12.21 (1.81)	12.20 (1.82)
expenditures food 2017 fats and oils etc	45.25 (6.04)	45.23 (6.05)
expenditures food 2017 margarine	2.49 (0.36)	2.49 (0.36)
expenditures food 2017 fats and oils	14.32 (1.90)	14.31 (1.90)
expenditures food 2017 salad dressings	11.58 (1.57)	11.58 (1.57)
expenditures food 2017 nondairy cream and imitation milk	8.06 (1.18)	8.06 (1.18)
expenditures food 2017 peanut butter	8.74 (1.29)	8.74 (1.30)

expenditures food 2017 miscellaneous foods	303.99 (41.68)	303.83 (41.78)
expenditures food 2017 frozen prepared foods	53.85 (7.23)	53.83 (7.24)
expenditures food 2017 canned and packaged soups	18.40 (2.49)	18.39 (2.49)
expenditures food 2017 potato chips nuts and other snacks	71.27 (9.96)	71.23 (9.98)
expenditures food 2017 condiments and seasonings	58.44 (8.27)	58.41 (8.29)
expenditures food 2017 other canned and packaged prepared foods	102.13 (14.02)	102.07 (14.05)
expenditures food 2017 prepared salads	16.74 (2.32)	16.73 (2.33)
expenditures food 2017 prepared desserts	5.42 (0.76)	5.42 (0.76)
expenditures food 2017 baby food	13.79 (2.13)	13.78 (2.13)
expenditures food 2017 nonalcoholic beverages	152.98 (20.67)	152.91 (20.71)
expenditures home 2017 housewares	33.15 (5.74)	33.14 (5.75)
expenditures home 2017 flatware	1.93 (0.48)	1.93 (0.48)
expenditures home 2017 nonelectric cookware	9.33 (1.68)	9.32 (1.68)
expenditures home 2017 tableware nonelectric kitchenware	9.90 (1.74)	9.90 (1.74)
expenditures home 2017 small appliances	15.56 (2.51)	15.56 (2.51)
expenditures home 2017 small electric kitchen appliances	11.96 (2.00)	11.96 (2.00)
expenditures home 2017 portable heating and cooling equipment	3.63 (0.61)	3.63 (0.61)
expenditures home 2017 miscellaneous household equipment	328.02 (57.81)	327.99 (57.93)
expenditures home 2017 window coverings	7.45 (2.02)	7.45 (2.02)
expenditures home 2017 infants equipment	11.93 (1.96)	11.93 (1.96)
expenditures home 2017 laundry and cleaning equipment	9.01 (1.44)	9.01 (1.44)
expenditures home 2017 outdoor equipment	8.98 (2.17)	8.97 (2.18)
expenditures home 2017 clocks and decorative items	57.10 (12.00)	57.08 (12.03)
expenditures home 2017 telephones and accessories	22.02 (5.62)	22.04 (5.63)
expenditures home 2017 lawn and garden equipment	22.97 (4.24)	22.96 (4.24)

expenditures home 2017 power tools	13.58 (2.48)	13.57 (2.49)
expenditures home 2017 office furniture for home use	3.06 (1.02)	3.06 (1.02)
expenditures home 2017 hand tools	6.08 (1.03)	6.08 (1.03)
expenditures home 2017 indoor plants fresh flowers	19.82 (3.73)	19.82 (3.73)
expenditures home 2017 closet and storage items	8.21 (1.61)	8.21 (1.61)
expenditures home 2017 rental of furniture	2.05 (0.77)	2.05 (0.77)
expenditures home 2017 luggage	5.97 (0.94)	5.97 (0.94)
expenditures home 2017 computers and computer hardware for non business use	55.54 (9.51)	55.53 (9.52)
expenditures home 2017 portable memory	2.00 (0.33)	2.00 (0.33)
expenditures home 2017 computer software	9.82 (1.84)	9.82 (1.85)
expenditures home 2017 computer accessories	13.81 (2.47)	13.80 (2.47)
expenditures home 2017 internet services away from home	2.54 (0.42)	2.54 (0.42)
expenditures home 2017 business equipment for home use	1.72 (0.29)	1.72 (0.30)
expenditures home 2017 other hardware	8.29 (1.87)	8.29 (1.87)
expenditures home 2017 miscellaneous household equipment and parts	18.25 (3.00)	18.25 (3.01)
expenditures home 2017 apparel and services	773.66 (140.48)	773.72 (140.70)
expenditures home 2017 men and boys	178.55 (29.95)	178.55 (29.98)
expenditures home 2017 men 16 and over	138.61 (24.16)	138.62 (24.19)
expenditures home 2017 mens suits	10.74 (2.97)	10.75 (2.98)
expenditures home 2017 mens sportcoats tailored jackets	3.44 (1.01)	3.44 (1.01)
expenditures home 2017 mens coats and jackets	10.22 (1.66)	10.22 (1.66)
expenditures home 2017 mens underwear	13.42 (2.20)	13.41 (2.20)
expenditures home 2017 mens hosiery	7.09 (1.17)	7.09 (1.17)
expenditures home 2017 mens nightwear	0.85 (0.15)	0.85 (0.15)
expenditures home 2017 mens accessories	13.01 (2.22)	13.01 (2.22)

expenditures home 2017 mens active sportswear	10.97 (1.92)	10.97 (1.92)
expenditures home 2017 mens pants and shorts	32.24 (5.49)	32.24 (5.49)
expenditures home 2017 mens uniforms	1.22 (0.20)	1.22 (0.20)
expenditures home 2017 mens costumes	0.52 (0.10)	0.52 (0.10)
expenditures home 2017 boys 2 to 15	42.53 (7.73)	42.53 (7.73)
expenditures home 2017 boys coats and jackets	2.68 (0.45)	2.68 (0.45)
expenditures home 2017 boys underwear	5.18 (0.80)	5.18 (0.80)
expenditures home 2017 boys nightwear	0.44 (0.07)	0.44 (0.07)
expenditures home 2017 boys hosiery	2.50 (0.45)	2.50 (0.45)
expenditures home 2017 boys accessories	2.82 (0.69)	2.82 (0.69)
expenditures home 2017 boys suits sportcoats vests	0.50 (0.10)	0.50 (0.10)
expenditures home 2017 boys pants and shorts	10.77 (2.35)	10.77 (2.35)
expenditures home 2017 boys costumes	0.45 (0.07)	0.45 (0.07)
expenditures home 2017 women and girls	290.68 (49.24)	290.68 (49.30)
expenditures home 2017 women 16 and over	247.03 (42.13)	247.03 (42.18)
expenditures home 2017 womens coats and jackets	13.35 (2.25)	13.35 (2.25)
expenditures home 2017 womens dresses	30.33 (5.74)	30.33 (5.74)
expenditures home 2017 womens sportcoats tailored jackets	3.74 (1.52)	3.75 (1.53)
expenditures home 2017 womens skirts	4.83 (0.87)	4.83 (0.87)
expenditures home 2017 womens pants and shorts	34.77 (5.84)	34.77 (5.85)
expenditures home 2017 interest paid home equity line of credit	38.75 (7.86)	38.74 (7.87)
expenditures home 2017 property taxes	822.77 (142.28)	822.62 (142.42)
expenditures home 2017 maintenance repairs ins other exp owned	593.42 (97.48)	593.22 (97.55)
expenditures home 2017 homeowners and related insurance	171.58 (28.80)	171.50 (28.82)
expenditures home 2017 ground rent	22.45 (5.91)	22.42 (5.91)

expenditures home 2017 maintenance and repair services owned	300.71 (51.38)	300.66 (51.42)
expenditures home 2017 rent	1299.80 (443.96)	1300.03 (444.29)
expenditures home 2017 rent as pay	32.95 (12.34)	32.95 (12.34)
expenditures home 2017 maintenance insurance and other expenses rented	42.63 (9.06)	42.65 (9.07)
expenditures home 2017 tenants insurance	7.82 (2.39)	7.82 (2.39)
expenditures home 2017 other lodging	318.03 (74.65)	318.15 (74.79)
expenditures home 2017 owned vacation homes	112.43 (27.53)	112.48 (27.59)
expenditures home 2017 utilities fuels and public services	1533.85 (241.94)	1533.45 (241.91)
expenditures home 2017 natural gas	166.26 (27.19)	166.24 (27.18)
expenditures home 2017 electricity	569.60 (92.64)	569.43 (92.63)
expenditures home 2017 fuel oil and other fuels	46.66 (8.25)	46.64 (8.26)
expenditures home 2017 fuel oil	25.22 (4.10)	25.21 (4.11)
expenditures home 2017 coal wood and other fuels	3.38 (0.78)	3.38 (0.78)
expenditures home 2017 bottled gas	18.12 (3.66)	18.11 (3.66)
expenditures home 2017 telephone services	536.93 (84.13)	536.81 (84.13)
expenditures home 2017 cell phone service	412.27 (64.21)	412.19 (64.21)
expenditures home 2017 water and other public services	216.86 (33.13)	216.80 (33.13)
expenditures home 2017 household operations	566.13 (95.01)	566.14 (95.08)
expenditures home 2017 personal services	212.48 (36.92)	212.49 (36.94)
expenditures home 2017 other household expenses	361.34 (61.96)	361.35 (62.00)
expenditures home 2017 housekeeping services	68.28 (21.87)	68.33 (21.93)
expenditures home 2017 gardening lawn care service	54.26 (10.85)	54.26 (10.87)
expenditures home 2017 water softening service	3.02 (0.45)	3.02 (0.45)
expenditures home 2017 household laundry and dry cleaning sent out nonclothing not coin operated	7.67 (1.86)	7.68 (1.86)
expenditures home 2017 coin operated household laundry and dry cleaning nonclothing	3.80 (1.32)	3.80 (1.32)

expenditures home 2017 services for termite pest control	8.16 (1.43)	8.16 (1.43)
expenditures home 2017 home security system service fee	11.93 (2.10)	11.94 (2.10)
expenditures home 2017 termite pest control products	2.04 (0.36)	2.04 (0.36)
expenditures home 2017 moving storage freight	18.56 (3.51)	18.56 (3.51)
expenditures home 2017 appliance repair including service center	6.40 (1.05)	6.40 (1.05)
expenditures home 2017 reupholstering furniture repair	1.96 (0.42)	1.96 (0.42)
expenditures home 2017 repairs rentals of lawn and garden equipment hand or power tools other household equipment	2.81 (0.54)	2.81 (0.54)
expenditures home 2017 computer information services internet	163.98 (26.25)	163.98 (26.27)
expenditures home 2017 computer installation	0.08 (0.02)	0.08 (0.02)
expenditures home 2017 housekeeping supplies	263.15 (42.79)	263.08 (42.80)
expenditures home 2017 laundry and cleaning supplies	62.00 (10.18)	62.01 (10.19)
expenditures home 2017 soaps and detergents	33.54 (5.55)	33.55 (5.56)
expenditures home 2017 other laundry cleaning products	28.45 (4.63)	28.45 (4.64)
expenditures home 2017 cleansing and toilet tissue paper towels and napkins	47.47 (7.77)	47.47 (7.77)
expenditures home 2017 miscellaneous household products	66.27 (11.32)	66.24 (11.33)
expenditures home 2017 lawn and garden supplies	35.86 (7.09)	35.83 (7.10)
expenditures home 2017 postage and stationery	52.61 (8.90)	52.59 (8.91)
expenditures home 2017 stationery stationery supplies giftwrap	31.25 (5.54)	31.23 (5.55)
expenditures home 2017 postage	19.85 (3.44)	19.85 (3.44)
expenditures home 2017 delivery services	1.69 (0.32)	1.69 (0.32)
expenditures home 2017 household furnishings and equipment	746.24 (124.57)	746.25 (124.69)
expenditures home 2017 household textiles	46.34 (7.60)	46.35 (7.62)
expenditures home 2017 bathroom linens	10.24 (1.95)	10.25 (1.96)
expenditures home 2017 bedroom linens	25.10 (4.29)	25.12 (4.30)
expenditures home 2017 curtains and draperies	5.14 (0.89)	5.14 (0.90)

expenditures home 2017 slipcovers decorative pillows	1.78 (0.29)	1.78 (0.29)
expenditures home 2017 furniture	206.98 (36.02)	207.05 (36.09)
expenditures home 2017 mattress and springs	40.55 (7.00)	40.56 (7.01)
expenditures home 2017 other bedroom furniture	35.28 (5.81)	35.29 (5.83)
expenditures home 2017 sofas	58.48 (10.53)	58.51 (10.56)
expenditures home 2017 living room chairs	19.92 (3.40)	19.92 (3.41)
expenditures home 2017 living room tables	4.50 (0.93)	4.50 (0.93)
expenditures home 2017 kitchen dining room furniture	17.73 (3.77)	17.74 (3.79)
expenditures home 2017 infants furniture	3.52 (0.60)	3.52 (0.60)
expenditures home 2017 outdoor furniture	10.82 (2.37)	10.82 (2.37)
expenditures home 2017 wall units cabinets and other occasional furniture	15.92 (2.87)	15.93 (2.88)
expenditures home 2017 floor coverings	7.31 (1.24)	7.31 (1.24)
expenditures home 2017 major appliances	109.53 (17.97)	109.50 (17.98)
expenditures home 2017 electric floor cleaning equipment	7.44 (1.25)	7.44 (1.25)
expenditures home 2017 sewing machines	2.28 (0.60)	2.28 (0.60)
expenditures home 2017 small appliances miscellaneous housewares	48.78 (8.26)	48.77 (8.26)
expenditures home 2017 other apparel products and services	122.77 (35.12)	122.90 (35.30)
expenditures home 2017 audio and visual equipment and services	422.27 (61.99)	422.12 (61.98)
expenditures home 2017 televisions	38.85 (6.06)	38.85 (6.06)
expenditures home 2017 cable and satellite television services	291.45 (43.77)	291.30 (43.75)
expenditures home 2017 satellite radio service	6.11 (1.03)	6.11 (1.03)
expenditures home 2017 sound equipment accessories	3.25 (0.56)	3.24 (0.56)
expenditures home 2017 online gaming services	1.42 (0.26)	1.42 (0.26)
expenditures home 2017 vcr s and video disc players	1.57 (0.23)	1.57 (0.23)
expenditures home 2017 video cassettes tapes and discs	9.61 (1.44)	9.61 (1.44)

expenditures home 2017 video game software	4.82 (0.73)	4.82 (0.73)
expenditures home 2017 video game hardware and accessories	19.76 (3.39)	19.74 (3.39)
expenditures home 2017 streaming downloading video	9.12 (1.47)	9.11 (1.47)
expenditures home 2017 applications games ringtones for handheld devices	1.29 (0.19)	1.29 (0.19)
expenditures home 2017 repair of tv radio and sound equipment	0.55 (0.09)	0.55 (0.09)
expenditures home 2017 rental of televisions	0.22 (0.19)	0.22 (0.19)
expenditures home 2017 personal digital audio players	0.81 (0.17)	0.81 (0.17)
expenditures home 2017 satellite dishes	0.54 (0.22)	0.54 (0.22)
expenditures home 2017 c ds records audio tapes	2.95 (0.51)	2.95 (0.51)
expenditures home 2017 streaming downloading audio	1.98 (0.41)	1.98 (0.41)
expenditures home 2017 musical instruments and accessories	9.45 (2.01)	9.46 (2.03)
expenditures home 2017 rental and repair of musical instruments	0.99 (0.25)	0.99 (0.25)
expenditures home 2017 rental of video cassettes tapes films and discs	9.30 (1.39)	9.30 (1.39)
expenditures home 2017 live entertainment for catered affairs	4.14 (0.98)	4.14 (0.98)
expenditures home 2017 rental of party supplies for catered affairs	5.40 (1.73)	5.40 (1.74)
expenditures home 2017 window air conditioners owner	1.49 (0.23)	1.49 (0.23)
expenditures home 2017 babysitting and child care	52.89 (11.24)	52.89 (11.25)
expenditures home 2017 boys shirts and sweaters	10.52 (1.66)	10.52 (1.66)
expenditures home 2017 boys uniforms and active sportswear	6.48 (1.03)	6.48 (1.03)
expenditures home 2017 kitchen dining room other linens	4.13 (0.78)	4.13 (0.78)
expenditures home 2017 dinnerware glassware serving pieces	12.07 (2.22)	12.06 (2.23)
expenditures home 2017 dishwashers built in garbage disposals range hoods owner	9.65 (1.80)	9.64 (1.80)
expenditures home 2017 flooring installation repair replacement owned	35.99 (6.55)	35.98 (6.56)
expenditures home 2017 girls shirts blouses sweaters and vests	12.30 (1.99)	12.30 (1.99)
expenditures home 2017 lamps lighting fixtures ceiling fans	13.53 (2.37)	13.53 (2.37)

expenditures home 2017 maintenance and repair services rented	15.00 (3.28)	15.01 (3.28)
expenditures home 2017 microwave ovens owner	3.53 (0.57)	3.53 (0.57)
expenditures home 2017 mens shirts sweaters and vests	35.11 (6.17)	35.11 (6.18)
expenditures home 2017 other household appliances owner	5.97 (1.42)	5.97 (1.42)
expenditures home 2017 cooking stoves oven owner	14.86 (2.69)	14.85 (2.69)
expenditures home 2017 refrigerators freezers owner	31.17 (5.73)	31.15 (5.74)
expenditures home 2017 stereos radios speakers and sound components including those in vehicles	6.17 (1.65)	6.17 (1.65)
expenditures home 2017 residential phone service voip and phone cards	126.56 (20.56)	126.53 (20.56)
expenditures home 2017 clothes washer or dryer owner	25.62 (4.31)	25.61 (4.31)
expenditures home 2017 womens sweaters shirts tops vests	68.70 (11.34)	68.69 (11.35)
expenditures home 2017 housing	7469.68 (1192.44)	7469.56 (1193.48)
expenditures home 2017 mortgage interest	1241.22 (241.28)	1241.17 (241.50)
expenditures home 2017 interest paid home equity loan	15.52 (3.03)	15.51 (3.03)
expenditures home 2017 womens sleepwear	9.07 (1.42)	9.07 (1.43)
expenditures home 2017 womens undergarments	14.38 (2.35)	14.38 (2.36)
expenditures home 2017 womens hosiery	9.01 (1.41)	9.01 (1.41)
expenditures home 2017 womens suits	2.31 (0.36)	2.31 (0.36)
expenditures home 2017 womens accessories	36.86 (9.36)	36.88 (9.40)
expenditures home 2017 womens uniforms	2.24 (0.34)	2.24 (0.34)
expenditures home 2017 womens costumes	0.63 (0.15)	0.63 (0.15)
expenditures home 2017 girls 2 to 15	45.88 (6.92)	45.87 (6.92)
expenditures home 2017 girls coats and jackets	2.23 (0.34)	2.23 (0.33)
expenditures home 2017 girls dresses and suits	6.13 (1.21)	6.13 (1.21)
expenditures home 2017 girls skirts pants and shorts	10.09 (1.52)	10.09 (1.52)
expenditures home 2017 girls active sportswear	5.34 (0.97)	5.34 (0.98)

expenditures home 2017 girls underwear and sleepwear	3.94 (0.59)	3.93 (0.59)
expenditures home 2017 girls hosiery	1.77 (0.29)	1.77 (0.29)
expenditures home 2017 girls accessories	2.06 (0.42)	2.06 (0.42)
expenditures home 2017 girls uniforms	1.03 (0.19)	1.03 (0.19)
expenditures home 2017 girls costumes	1.11 (0.21)	1.11 (0.21)
expenditures home 2017 children under 2	36.35 (5.75)	36.35 (5.76)
expenditures home 2017 infant coat jacket snowsuit	0.44 (0.07)	0.44 (0.07)
expenditures home 2017 infant dresses outerwear	10.86 (2.22)	10.86 (2.22)
expenditures home 2017 infant underwear	15.93 (2.34)	15.92 (2.34)
expenditures home 2017 infant nightwear loungewear	2.75 (0.42)	2.74 (0.42)
expenditures home 2017 infant accessories	6.68 (1.61)	6.68 (1.62)
expenditures home 2017 footwear	148.52 (22.91)	148.51 (22.93)
expenditures home 2017 mens footwear	47.40 (7.13)	47.39 (7.13)
expenditures home 2017 boys footwear	21.30 (3.89)	21.30 (3.89)
expenditures home 2017 womens footwear	65.27 (11.77)	65.28 (11.80)
expenditures home 2017 girls footwear	16.46 (2.70)	16.46 (2.70)
expenditures miscellaneous 2017 automobile service clubs and gps services	10.51 (1.36)	10.51 (1.36)
expenditures miscellaneous 2017 cash downpayment car truck lease	9.68 (1.34)	9.69 (1.35)
expenditures miscellaneous 2017 car truck lease payments	106.92 (19.08)	106.96 (19.13)
expenditures miscellaneous 2017 auto truck rental	17.80 (3.21)	17.81 (3.22)
expenditures miscellaneous 2017 auto truck rental out of town trips	9.36 (2.03)	9.37 (2.03)
expenditures miscellaneous 2017 termination fee for car truck lease	0.17 (0.03)	0.17 (0.03)
expenditures miscellaneous 2017 trade in allowance for car truck lease	15.66 (1.99)	15.66 (1.99)
expenditures miscellaneous 2017 finance late interest charges for credit cards	111.50 (12.32)	111.49 (12.33)
expenditures miscellaneous 2017 finance late interest charges for other loans	16.94 (2.48)	16.93 (2.48)

expenditures miscellaneous 2017 finance late interest charges for student loans	61.22 (8.74)	61.24 (8.76)
expenditures miscellaneous 2017 gas tank repair replacement	5.08 (1.22)	5.08 (1.22)
expenditures miscellaneous 2017 fee for service health plan bcbs	219.65 (35.52)	219.57 (35.56)
expenditures miscellaneous 2017 fee for service health plan not bcbs	236.94 (36.95)	236.91 (37.00)
expenditures miscellaneous 2017 health maintenance organization bcbs	166.80 (20.40)	166.78 (20.40)
expenditures miscellaneous 2017 health maintenance organization not bcbs	251.51 (31.16)	251.46 (31.16)
expenditures miscellaneous 2017 magazines	0.01 (0.00)	0.01 (0.00)
expenditures miscellaneous 2017 material and supplies for sewing needlework quilting	8.89 (1.71)	8.88 (1.71)
expenditures miscellaneous 2017 movies parks museums	25.69 (4.37)	25.70 (4.38)
expenditures miscellaneous 2017 newspapers	0.02 (0.00)	0.02 (0.00)
expenditures miscellaneous 2017 play theater opera concert	19.79 (5.32)	19.79 (5.33)
expenditures miscellaneous 2017 value of savings checking money market and c ds	5239.25 (1047.28)	5237.92 (1049.11)
expenditures miscellaneous 2017 value surrender of whole life insurance policy	3322.14 (1031.53)	3324.11 (1034.28)
expenditures miscellaneous 2017 value of other financial assets	1862.47 (573.75)	1862.73 (575.27)
expenditures miscellaneous 2017 value of retirement plan	29820.78 (6626.41)	29818.33 (6640.26)
expenditures miscellaneous 2017 value of stocks bonds mutual funds	6357.62 (1930.08)	6359.04 (1935.39)
expenditures miscellaneous 2017 vehicle or engine repairs	76.69 (10.36)	76.67 (10.36)
expenditures miscellaneous 2017 care for elderly invalids handicapped etc	12.03 (2.75)	12.01 (2.76)
expenditures miscellaneous 2017 day care centers nursery and preschools	146.05 (22.90)	146.09 (23.01)
expenditures miscellaneous 2017 watches	28.04 (11.60)	28.07 (11.63)
expenditures miscellaneous 2017 jewelry	32.47 (8.95)	32.49 (8.98)
expenditures miscellaneous 2017 shoe repair and other shoe service	0.68 (0.14)	0.68 (0.14)
expenditures miscellaneous 2017 coin operated apparel laundry and dry cleaning	14.32 (2.66)	14.33 (2.68)
expenditures miscellaneous 2017 alteration repair and tailoring of apparel and accessories	2.66 (0.51)	2.66 (0.52)
expenditures miscellaneous 2017 clothing rental	0.86 (0.19)	0.86 (0.19)

expenditures miscellaneous 2017 watch and jewelry repair	12.36 (6.41)	12.37 (6.43)
expenditures miscellaneous 2017 apparel laundry and dry cleaning not coin operated	18.66 (5.30)	18.68 (5.31)
expenditures miscellaneous 2017 transportation	3879.86 (497.37)	3879.45 (497.60)
expenditures miscellaneous 2017 vehicle purchases net outlay	1647.71 (215.91)	1647.47 (215.99)
expenditures miscellaneous 2017 cars and trucks new	821.21 (131.57)	821.11 (131.73)
expenditures miscellaneous 2017 new cars	304.88 (40.61)	304.91 (40.64)
expenditures miscellaneous 2017 new trucks	518.09 (92.85)	517.96 (92.99)
expenditures miscellaneous 2017 cars and trucks used	802.29 (82.13)	802.16 (82.14)
expenditures miscellaneous 2017 used cars	347.68 (36.06)	347.72 (36.09)
expenditures miscellaneous 2017 used trucks	453.01 (53.13)	452.84 (53.16)
expenditures miscellaneous 2017 new motorcycles	12.80 (2.68)	12.80 (2.69)
expenditures miscellaneous 2017 used motorcycles	10.87 (1.60)	10.87 (1.60)
expenditures miscellaneous 2017 gasoline and motor oil	842.98 (94.83)	842.77 (94.82)
expenditures miscellaneous 2017 vehicle finance charges	87.53 (10.03)	87.52 (10.03)
expenditures miscellaneous 2017 maintenance and repairs	338.99 (42.87)	338.91 (42.88)
expenditures miscellaneous 2017 coolant brake fluid transmission fluid and other additives	2.88 (0.28)	2.88 (0.28)
expenditures miscellaneous 2017 tires purchased replaced installed	52.15 (7.15)	52.14 (7.15)
expenditures miscellaneous 2017 parts equipment and accessories	20.20 (2.97)	20.18 (2.97)
expenditures miscellaneous 2017 vehicle products and cleaning services	5.60 (0.83)	5.59 (0.83)
expenditures miscellaneous 2017 misc auto repair servicing	40.20 (5.26)	40.20 (5.26)
expenditures miscellaneous 2017 body work and painting	12.71 (1.94)	12.71 (1.94)
expenditures miscellaneous 2017 motor tune up	16.71 (2.30)	16.71 (2.30)
expenditures miscellaneous 2017 lube oil change and oil filters	35.25 (3.91)	35.24 (3.91)
expenditures miscellaneous 2017 front end alignment wheel balance and rotation	10.29 (1.21)	10.29 (1.21)
expenditures miscellaneous 2017 shock absorber replacement	5.10 (0.65)	5.10 (0.65)

expenditures miscellaneous 2017 repair tires and other repair work	42.75 (4.93)	42.76 (4.93)
expenditures miscellaneous 2017 auto repair service policy	13.86 (1.77)	13.85 (1.77)
expenditures miscellaneous 2017 vehicle insurance	433.09 (46.97)	433.00 (46.93)
expenditures miscellaneous 2017 vehicle rental leases licenses and other charges	260.72 (42.06)	260.77 (42.15)
expenditures miscellaneous 2017 rented vehicles	27.60 (5.30)	27.61 (5.31)
expenditures miscellaneous 2017 leased vehicles	132.79 (21.15)	132.82 (21.19)
expenditures miscellaneous 2017 vehicle registration state	38.42 (5.44)	38.40 (5.44)
expenditures miscellaneous 2017 vehicle registration local	3.89 (0.82)	3.89 (0.82)
expenditures miscellaneous 2017 drivers license	3.52 (0.35)	3.52 (0.35)
expenditures miscellaneous 2017 vehicle inspection	5.05 (0.64)	5.05 (0.64)
expenditures miscellaneous 2017 parking fees	20.10 (5.05)	20.12 (5.07)
expenditures miscellaneous 2017 parking fees in home city excluding residence	17.14 (4.37)	17.16 (4.39)
expenditures miscellaneous 2017 parking fees out of town trips	2.94 (0.61)	2.94 (0.61)
expenditures miscellaneous 2017 tolls or electronic toll passes	15.75 (3.01)	15.76 (3.01)
expenditures miscellaneous 2017 tolls on out of town trips	2.21 (0.38)	2.21 (0.38)
expenditures miscellaneous 2017 towing charges	1.64 (0.19)	1.64 (0.19)
expenditures miscellaneous 2017 public and other transportation	284.81 (75.13)	285.04 (75.46)
expenditures miscellaneous 2017 airline fares	181.47 (51.18)	181.62 (51.41)
expenditures miscellaneous 2017 intercity bus fares	4.60 (0.64)	4.60 (0.64)
expenditures miscellaneous 2017 intracity mass transit fares	35.12 (6.64)	35.16 (6.68)
expenditures miscellaneous 2017 local trans on out of town trips	5.61 (1.60)	5.61 (1.61)
expenditures miscellaneous 2017 taxi fares and limousine services on trips	3.29 (0.94)	3.29 (0.94)
expenditures miscellaneous 2017 taxi fares and limousine services	13.74 (2.93)	13.75 (2.94)
expenditures miscellaneous 2017 intercity train fares	7.20 (1.59)	7.20 (1.59)
expenditures miscellaneous 2017 ship fares	30.48 (7.94)	30.48 (7.96)

expenditures miscellaneous 2017 school bus	2.83 (1.48)	2.84 (1.49)
expenditures miscellaneous 2017 healthcare	1763.55 (260.51)	1762.66 (260.57)
expenditures miscellaneous 2017 health insurance	1203.61 (169.20)	1203.08 (169.20)
expenditures miscellaneous 2017 commercial health insurance	236.94 (36.95)	236.91 (37.00)
expenditures miscellaneous 2017 blue cross blue shield	412.38 (58.59)	412.25 (58.62)
expenditures miscellaneous 2017 commercial medicare supplement bcbs	15.29 (4.27)	15.26 (4.27)
expenditures miscellaneous 2017 other health insurance bcbs	12.17 (1.78)	12.17 (1.78)
expenditures miscellaneous 2017 medicare payments	175.41 (38.83)	175.23 (38.81)
expenditures miscellaneous 2017 medicare prescription drug premium	38.35 (9.03)	38.30 (9.02)
expenditures miscellaneous 2017 commercial medicare supplements and other health insurance	77.44 (17.03)	77.35 (17.04)
expenditures miscellaneous 2017 commercial medicare supplement not blue cross	42.19 (11.68)	42.13 (11.68)
expenditures miscellaneous 2017 other health insurance not bcbs	35.59 (6.01)	35.57 (6.01)
expenditures miscellaneous 2017 long term care insurance	33.40 (6.06)	33.38 (6.06)
expenditures miscellaneous 2017 medical services	329.47 (56.13)	329.28 (56.19)
expenditures miscellaneous 2017 physician s services	72.73 (11.57)	72.69 (11.59)
expenditures miscellaneous 2017 dental services	119.03 (20.21)	118.96 (20.23)
expenditures miscellaneous 2017 eyecare services	18.59 (2.77)	18.58 (2.78)
expenditures miscellaneous 2017 service by professionals other than physician	24.96 (4.47)	24.95 (4.48)
expenditures miscellaneous 2017 lab tests x rays	19.47 (3.44)	19.47 (3.45)
expenditures miscellaneous 2017 hospital room and services	55.63 (10.15)	55.59 (10.17)
expenditures miscellaneous 2017 care in convalescent or nursing home	9.39 (2.87)	9.37 (2.88)
expenditures miscellaneous 2017 other medical care services	10.51 (2.29)	10.51 (2.30)
expenditures miscellaneous 2017 drugs	171.61 (28.15)	171.49 (28.16)
expenditures miscellaneous 2017 nonprescription drugs	40.71 (6.31)	40.68 (6.31)
expenditures miscellaneous 2017 nonprescription vitamins	23.61 (3.79)	23.59 (3.79)

expenditures miscellaneous 2017 prescription drugs	107.32 (18.34)	107.23 (18.34)
expenditures miscellaneous 2017 medical supplies	59.92 (9.21)	59.88 (9.21)
expenditures miscellaneous 2017 eyeglasses and contact lenses	29.78 (4.14)	29.77 (4.14)
expenditures miscellaneous 2017 hearing aids	9.27 (2.60)	9.25 (2.61)
expenditures miscellaneous 2017 topicals and dressings	16.69 (2.58)	16.68 (2.58)
expenditures miscellaneous 2017 adult diapers	1.08 (0.13)	1.08 (0.13)
expenditures miscellaneous 2017 medical equipment for general use	1.78 (0.39)	1.78 (0.39)
expenditures miscellaneous 2017 supportive and convalescent medical equipment	1.27 (0.25)	1.27 (0.25)
expenditures miscellaneous 2017 rental of medical equipment	0.23 (0.05)	0.23 (0.05)
expenditures miscellaneous 2017 rental of supportive convalescent medical equipment	0.22 (0.04)	0.22 (0.04)
expenditures miscellaneous 2017 entertainment	1169.88 (187.83)	1169.63 (188.07)
expenditures miscellaneous 2017 fees and admissions	288.89 (67.95)	288.95 (68.16)
expenditures miscellaneous 2017 recreation expenses out of town trips	9.22 (2.17)	9.23 (2.18)
expenditures miscellaneous 2017 social recreation health club membership	71.95 (18.10)	71.96 (18.14)
expenditures miscellaneous 2017 fees for participant sports	41.65 (8.17)	41.62 (8.19)
expenditures miscellaneous 2017 participant sports out of town trips	8.72 (2.50)	8.72 (2.51)
expenditures miscellaneous 2017 movie other admissions out of town trips	25.02 (6.36)	25.03 (6.39)
expenditures miscellaneous 2017 admission to sporting events	19.25 (5.65)	19.25 (5.66)
expenditures miscellaneous 2017 admission to sports events out of town trips	8.33 (2.12)	8.34 (2.13)
expenditures miscellaneous 2017 fees for recreational lessons	51.39 (13.82)	51.43 (13.90)
expenditures miscellaneous 2017 other entertainment services out of town trips	9.22 (2.17)	9.23 (2.18)
expenditures miscellaneous 2017 pets toys hobbies and playground equipment	270.30 (47.33)	270.14 (47.40)
expenditures miscellaneous 2017 pets	217.17 (38.39)	217.03 (38.44)
expenditures miscellaneous 2017 pet food	95.51 (17.15)	95.43 (17.17)
expenditures miscellaneous 2017 pet purchase supplies medicine	47.63 (8.38)	47.60 (8.39)

expenditures miscellaneous 2017 pet services	20.69 (4.59)	20.69 (4.60)
expenditures miscellaneous 2017 vet services	53.95 (9.60)	53.92 (9.62)
expenditures miscellaneous 2017 toys games arts and crafts and tricycles	51.92 (8.54)	51.90 (8.55)
expenditures miscellaneous 2017 stamp and coin collecting	1.61 (0.39)	1.61 (0.39)
expenditures miscellaneous 2017 playground equipment	1.63 (0.26)	1.63 (0.26)
expenditures miscellaneous 2017 other entertainment supplies equipment and services	193.31 (39.80)	193.25 (39.87)
expenditures miscellaneous 2017 unmotored recreational vehicles	33.57 (7.95)	33.53 (7.96)
expenditures miscellaneous 2017 boat without motor and boat trailers	4.35 (1.29)	4.35 (1.30)
expenditures miscellaneous 2017 trailer and other attachable campers	29.42 (7.46)	29.38 (7.47)
expenditures miscellaneous 2017 motorized recreational vehicles	61.36 (15.11)	61.32 (15.15)
expenditures miscellaneous 2017 purchase of motorized camper	24.40 (6.32)	24.38 (6.33)
expenditures miscellaneous 2017 purchase of other vehicle	21.56 (5.69)	21.55 (5.71)
expenditures miscellaneous 2017 purchase of boat with motor	16.02 (3.74)	16.01 (3.74)
expenditures miscellaneous 2017 sports recreation and exercise equipment	56.24 (11.33)	56.25 (11.36)
expenditures miscellaneous 2017 athletic gear game tables and exercise equipment	18.81 (3.65)	18.81 (3.66)
expenditures miscellaneous 2017 bicycles	9.95 (1.86)	9.95 (1.86)
expenditures miscellaneous 2017 camping equipment	4.91 (0.86)	4.91 (0.87)
expenditures miscellaneous 2017 hunting and fishing equipment	13.90 (3.22)	13.89 (3.23)
expenditures miscellaneous 2017 winter sports equipment	2.24 (0.50)	2.24 (0.50)
expenditures miscellaneous 2017 water sports equipment	1.84 (0.49)	1.84 (0.49)
expenditures miscellaneous 2017 other sports equipment	3.30 (0.60)	3.30 (0.60)
expenditures miscellaneous 2017 global positioning system devices	0.43 (0.34)	0.43 (0.34)
expenditures miscellaneous 2017 rental and repair of miscellaneous sports equipment	0.67 (0.15)	0.67 (0.15)
expenditures miscellaneous 2017 photographic equipment supplies and services	18.40 (2.63)	18.40 (2.64)
expenditures miscellaneous 2017 film	0.44 (0.10)	0.44 (0.10)

expenditures miscellaneous 2017 other photographic supplies	0.24 (0.08)	0.24 (0.08)
expenditures miscellaneous 2017 photo processing	2.98 (0.58)	2.98 (0.58)
expenditures miscellaneous 2017 photographic equipment	7.32 (1.33)	7.32 (1.35)
expenditures miscellaneous 2017 photographer fees	7.22 (1.00)	7.21 (1.00)
expenditures miscellaneous 2017 fireworks	2.62 (0.74)	2.62 (0.74)
expenditures miscellaneous 2017 visual goods	0.35 (0.08)	0.35 (0.08)
expenditures miscellaneous 2017 pinball electronic video games	0.81 (0.15)	0.81 (0.15)
expenditures miscellaneous 2017 personal care products and services	276.74 (35.56)	276.73 (35.58)
expenditures miscellaneous 2017 personal care products	153.12 (19.82)	153.10 (19.83)
expenditures miscellaneous 2017 hair care products	30.02 (3.75)	30.01 (3.75)
expenditures miscellaneous 2017 nonelectric articles for the hair	5.68 (0.80)	5.67 (0.80)
expenditures miscellaneous 2017 wigs and hairpieces	0.89 (0.67)	0.89 (0.68)
expenditures miscellaneous 2017 oral hygiene products articles	17.85 (2.23)	17.84 (2.23)
expenditures miscellaneous 2017 shaving needs	9.03 (1.41)	9.03 (1.41)
expenditures miscellaneous 2017 cosmetics perfume bath preparations	68.21 (9.03)	68.20 (9.04)
expenditures miscellaneous 2017 deodorants feminine hygiene miscellaneous personal care	17.39 (2.32)	17.39 (2.32)
expenditures miscellaneous 2017 electric personal care appliances	4.18 (0.65)	4.18 (0.66)
expenditures miscellaneous 2017 personal care services	123.61 (16.35)	123.63 (16.36)
expenditures miscellaneous 2017 reading	46.06 (7.63)	46.04 (7.64)
expenditures miscellaneous 2017 books thru book clubs	1.43 (0.31)	1.43 (0.31)
expenditures miscellaneous 2017 books not thru book clubs	11.18 (2.60)	11.18 (2.60)
expenditures miscellaneous 2017 digital book readers	9.87 (1.68)	9.87 (1.69)
expenditures miscellaneous 2017 school supplies etc unspecified	24.19 (3.25)	24.19 (3.26)
expenditures miscellaneous 2017 tobacco products and smoking supplies	133.26 (16.38)	133.18 (16.40)
expenditures miscellaneous 2017 other tobacco products	13.37 (2.02)	13.36 (2.02)

expenditures miscellaneous 2017 smoking accessories	3.20 (0.47)	3.20 (0.47)
expenditures miscellaneous 2017 miscellaneous	355.64 (53.13)	355.57 (53.19)
expenditures miscellaneous 2017 lotteries and pari mutuel losses	21.08 (3.66)	21.06 (3.66)
expenditures miscellaneous 2017 legal fees	63.67 (13.11)	63.66 (13.14)
expenditures miscellaneous 2017 funeral expenses	26.83 (5.08)	26.80 (5.08)
expenditures miscellaneous 2017 safe deposit box rental	1.64 (0.30)	1.64 (0.30)
expenditures miscellaneous 2017 checking accounts other bank service charges	11.78 (1.16)	11.77 (1.16)
expenditures miscellaneous 2017 cemetery lots vaults maintenance fees	2.31 (0.47)	2.30 (0.47)
expenditures miscellaneous 2017 accounting fees	26.64 (6.28)	26.64 (6.29)
expenditures miscellaneous 2017 miscellaneous personal services	16.52 (2.53)	16.51 (2.53)
expenditures miscellaneous 2017 dating services	0.24 (0.04)	0.24 (0.04)
expenditures miscellaneous 2017 occupational expenses	18.76 (2.91)	18.76 (2.91)
expenditures miscellaneous 2017 expenses for other properties	17.22 (3.38)	17.21 (3.39)
expenditures miscellaneous 2017 credit card memberships	2.09 (0.61)	2.09 (0.61)
expenditures miscellaneous 2017 shopping club membership fees	7.37 (1.12)	7.37 (1.12)
expenditures miscellaneous 2017 vacation clubs	12.16 (6.14)	12.18 (6.16)
expenditures miscellaneous 2017 cash contributions	742.11 (126.87)	742.13 (127.03)
expenditures miscellaneous 2017 support for college students	42.38 (8.22)	42.40 (8.25)
expenditures miscellaneous 2017 alimony expenditures	29.89 (6.08)	29.89 (6.09)
expenditures miscellaneous 2017 child support expenditures	85.25 (9.87)	85.24 (9.90)
expenditures miscellaneous 2017 gift to non cu members of stocks bonds and mutual funds	25.19 (4.06)	25.19 (4.06)
expenditures miscellaneous 2017 cash contributions to charities and other organizations	92.80 (25.62)	92.82 (25.69)
expenditures miscellaneous 2017 cash contributions to church religious organizations	317.82 (45.37)	317.79 (45.39)
expenditures miscellaneous 2017 cash contribution to educational institutions	16.14 (6.26)	16.15 (6.28)
expenditures miscellaneous 2017 cash contribution to political organizations	3.24 (0.67)	3.23 (0.68)

expenditures miscellaneous 2017 other cash gifts	130.24 (19.61)	130.20 (19.61)
expenditures miscellaneous 2017 personal insurance and pensions	2768.03 (594.74)	2769.16 (596.42)
expenditures miscellaneous 2017 life and other personal insurance	141.35 (24.85)	141.37 (24.89)

HH=Household
Fam=Family
Pop=Population
Non Fam=Non family
OT=Other
ER=Emergency room
RV=recreational vehicle
Equip=equipment
Misc.=miscellaneous
BCBS=Blue Cross Blue Shield
OOT=Out of town
RIHC=resource intensive healthcare
