# Supplementary material for: Can diverse population characteristics be leveraged in a machine learning pipeline to predict resource intensive healthcare utilization among hospital service areas?
Source: BMC Health Serv Res. 2022 Jun 30;22:847. doi: 10.1186/s12913-022-08154-4 (PMC9248096; doi:10.1186/s12913-022-08154-4)
Supplement: Supplementary file 9 — Additional file 9. [file 12913_2022_8154_MOESM9_ESM.pdf]

Additional File 9. Descriptive Statistics for Consumer Expenditures Candidate Predictors (second order terms)

- Additional File 9
  - File format: PDF
  - File title: Descriptive Statistics for Consumer Expenditures Candidate Predictors (second order terms)
  - File description: Long table with univariate results for second order terms

ER Visits (mean(SD))	
expenditures food 2017 dinner at employer and school cafeterias total amount expenditures miscellaneous 2017 wigs and hairpieces total amount	2.09 (1.31)
expenditures food 2017 education total amount expenditures miscellaneous 2017 wigs and hairpieces total amount	510.65 (297.03)
expenditures food 2017 test preparation tutoring services total amount expenditures miscellaneous 2017 wigs and hairpieces total amount	6.52 (4.02)
expenditures food 2017 other schools tuition total amount expenditures miscellaneous 2017 wigs and hairpieces total amount	3.16 (1.82)
expenditures food 2017 school lunches total amount expenditures miscellaneous 2017 wigs and hairpieces total amount	22.36 (13.73)
expenditures food 2017 alcoholic beverages away from home total amount expenditures miscellaneous 2017 wigs and hairpieces total amount	80.15 (43.68)
expenditures food 2017 wine away total amount expenditures miscellaneous 2017 wigs and hairpieces total amount	15.68 (8.68)
expenditures food 2017 candy and chewing gum total amount expenditures miscellaneous 2017 wigs and hairpieces total amount	36.04 (20.43)
expenditures food 2017 rice total amount expenditures miscellaneous 2017 wigs and hairpieces total amount	9.49 (6.14)
expenditures food 2017 pies tarts turnovers total amount expenditures miscellaneous 2017 wigs and hairpieces total amount	5.39 (3.14)
expenditures food 2017 beef total amount expenditures miscellaneous 2017 wigs and hairpieces total amount	84.81 (50.45)
expenditures food 2017 margarine total amount expenditures miscellaneous 2017 wigs and hairpieces total amount	2.11 (1.31)
expenditures food 2017 peanut butter total amount expenditures miscellaneous 2017 wigs and hairpieces total amount	7.38 (4.45)
expenditures food 2017 baby food total amount expenditures miscellaneous 2017 wigs and hairpieces total amount	11.62 (7.02)
expenditures home 2017 telephones and accessories total amount expenditures miscellaneous 2017 wigs and hairpieces total amount	19.14 (13.62)
expenditures home 2017 rental of furniture total amount expenditures miscellaneous 2017 wigs and hairpieces total amount	1.91 (1.89)
expenditures home 2017 mens nightwear total amount expenditures miscellaneous 2017 wigs and hairpieces total amount	0.71 (0.42)
expenditures home 2017 boys suits sportcoats vests total amount expenditures miscellaneous 2017 wigs and hairpieces total amount	0.42 (0.26)

expenditures home 2017 rent total amount expenditures miscellaneous 2017 wigs and hairpieces total amount	1216.59 (1317.84)
expenditures home 2017 maintenance insurance and other expenses rented total amount expenditures miscellaneous 2017 wigs and hairpieces total amount	36.79 (25.36)
expenditures home 2017 tenants insurance total amount expenditures miscellaneous 2017 wigs and hairpieces total amount	7.25 (7.49)
expenditures home 2017 electricity total amount expenditures miscellaneous 2017 wigs and hairpieces total amount	497.92 (371.43)
expenditures home 2017 fuel oil total amount expenditures miscellaneous 2017 wigs and hairpieces total amount	21.26 (12.98)
expenditures home 2017 housekeeping services total amount expenditures miscellaneous 2017 wigs and hairpieces total amount	55.11 (29.69)
expenditures home 2017 coin operated household laundry and dry cleaning nonclothing total amount expenditures miscellaneous 2017 wigs and hairpieces total amount	3.73 (4.59)
expenditures home 2017 computer installation total amount expenditures miscellaneous 2017 wigs and hairpieces total amount	0.07 (0.05)
expenditures home 2017 infants furniture total amount expenditures miscellaneous 2017 wigs and hairpieces total amount	3.03 (2.00)
expenditures home 2017 repair of tv radio and sound equipment total amount expenditures miscellaneous 2017 wigs and hairpieces total amount	0.49 (0.35)
expenditures home 2017 c ds records audio tapes total amount expenditures miscellaneous 2017 wigs and hairpieces total amount	2.46 (1.40)
expenditures home 2017 boys uniforms and active sportswear total amount expenditures miscellaneous 2017 wigs and hairpieces total amount	5.57 (3.66)
expenditures home 2017 girls active sportswear total amount expenditures miscellaneous 2017 wigs and hairpieces total amount	4.40 (2.35)
expenditures home 2017 girls costumes total amount expenditures miscellaneous 2017 wigs and hairpieces total amount	0.90 (0.46)
expenditures miscellaneous 2017 auto truck rental total amount expenditures miscellaneous 2017 wigs and hairpieces total amount	15.64 (11.30)
expenditures miscellaneous 2017 termination fee for car truck lease total amount expenditures miscellaneous 2017 wigs and hairpieces total amount	0.15 (0.12)
expenditures miscellaneous 2017 gas tank repair replacement total amount expenditures miscellaneous 2017 wigs and hairpieces total amount	3.95 (1.59)
expenditures miscellaneous 2017 value surrender of whole life insurance policy total amount expenditures miscellaneous 2017 wigs and hairpieces total amount	2752.00 (1629.23)
expenditures miscellaneous 2017 day care centers nursery and preschools total amount expenditures miscellaneous 2017 wigs and hairpieces total amount	123.27 (73.04)
expenditures miscellaneous 2017 watches total amount expenditures miscellaneous 2017 wigs and hairpieces total amount	23.61 (16.27)
expenditures miscellaneous 2017 coin operated apparel laundry and dry cleaning total amount expenditures miscellaneous 2017 wigs and hairpieces total amount	14.19 (15.34)
expenditures miscellaneous 2017 apparel laundry and dry cleaning not coin operated total amount expenditures miscellaneous 2017 wigs and hairpieces total amount	16.24 (11.76)
expenditures miscellaneous 2017 parking fees total amount expenditures miscellaneous 2017 wigs and hairpieces total amount	16.95 (10.45)
expenditures miscellaneous 2017 parking fees in home city excluding residence total amount expenditures miscellaneous 2017 wigs and hairpieces total amount	14.50 (9.10)
expenditures miscellaneous 2017 intracity mass transit fares total amount expenditures miscellaneous 2017 wigs and hairpieces total amount	33.82 (33.34)

expenditures miscellaneous 2017 school bus total amount expenditures miscellaneous 2017 wigs and hairpieces total amount	2.24 (1.40)
expenditures miscellaneous 2017 medicare payments total amount expenditures miscellaneous 2017 wigs and hairpieces total amount	147.61 (92.88)
expenditures miscellaneous 2017 medical equipment for general use total amount expenditures miscellaneous 2017 wigs and hairpieces total amount	1.45 (0.77)
expenditures miscellaneous 2017 playground equipment total amount expenditures miscellaneous 2017 wigs and hairpieces total amount	1.32 (0.62)
expenditures miscellaneous 2017 camping equipment total amount expenditures miscellaneous 2017 wigs and hairpieces total amount	4.20 (2.66)
expenditures miscellaneous 2017 global positioning system devices total amount expenditures miscellaneous 2017 wigs and hairpieces total amount	0.58 (1.11)
expenditures miscellaneous 2017 other photographic supplies total amount expenditures miscellaneous 2017 wigs and hairpieces total amount	0.19 (0.09)
expenditures miscellaneous 2017 photographic equipment total amount expenditures miscellaneous 2017 wigs and hairpieces total amount	6.42 (4.58)
expenditures miscellaneous 2017 visual goods total amount expenditures miscellaneous 2017 wigs and hairpieces total amount	0.28 (0.12)
expenditures miscellaneous 2017 digital book readers total amount expenditures miscellaneous 2017 wigs and hairpieces total amount	8.49 (5.53)
expenditures miscellaneous 2017 occupational expenses total amount expenditures miscellaneous 2017 wigs and hairpieces total amount	15.97 (9.96)
expenditures miscellaneous 2017 vacation clubs total amount expenditures miscellaneous 2017 wigs and hairpieces total amount	9.85 (6.46)
expenditures miscellaneous 2017 support for college students total amount expenditures miscellaneous 2017 wigs and hairpieces total amount	36.02 (22.54)
Inpatient Days (mean(SD))	
expenditures food 2017 lunch at vending machines and mobile vendors expenditures miscellaneous 2017 coin operated apparel laundry and dry cleaning	77.72 (15.55)
expenditures food 2017 dinner at employer and school cafeterias expenditures miscellaneous 2017 coin operated apparel laundry and dry cleaning	34.81 (8.09)
expenditures food 2017 breakfast and brunch at full service restaurants expenditures miscellaneous 2017 coin operated apparel laundry and dry cleaning	705.35 (139.62)
expenditures food 2017 education expenditures miscellaneous 2017 coin operated apparel laundry and dry cleaning	8667.61 (1985.91)
expenditures food 2017 college tuition expenditures miscellaneous 2017 coin operated apparel laundry and dry cleaning	5254.71 (1272.63)
expenditures food 2017 test preparation tutoring services expenditures miscellaneous 2017 coin operated apparel laundry and dry cleaning	111.37 (38.17)
expenditures food 2017 other schools tuition expenditures miscellaneous 2017 coin operated apparel laundry and dry cleaning	54.11 (14.73)
expenditures food 2017 other school expenses including rentals expenditures miscellaneous 2017 coin operated apparel laundry and dry cleaning	282.00 (60.72)
expenditures food 2017 school books supplies equipment for elementary high school expenditures miscellaneous 2017 coin operated apparel laundry and dry cleaning	101.22 (21.86)
expenditures food 2017 school lunches expenditures miscellaneous 2017 coin operated apparel laundry and dry cleaning	376.04 (82.27)
expenditures food 2017 meals as pay expenditures miscellaneous 2017 coin operated apparel laundry and dry cleaning	161.31 (44.87)

expenditures food 2017 wine expenditures miscellaneous 2017 coin operated apparel laundry and dry cleaning	809.71 (178.76)
expenditures food 2017 alcoholic beverages purchased on trips expenditures miscellaneous 2017 coin operated apparel laundry and dry cleaning	366.36 (84.19)
expenditures food 2017 fresh fish and shellfish expenditures miscellaneous 2017 coin operated apparel laundry and dry cleaning	381.41 (81.02)
expenditures food 2017 artificial sweeteners expenditures miscellaneous 2017 coin operated apparel laundry and dry cleaning	24.82 (5.18)
expenditures food 2017 jams preserves other sweets expenditures miscellaneous 2017 coin operated apparel laundry and dry cleaning	173.85 (35.32)
expenditures food 2017 fats and oils expenditures miscellaneous 2017 coin operated apparel laundry and dry cleaning	204.74 (42.74)
expenditures food 2017 prepared desserts expenditures miscellaneous 2017 coin operated apparel laundry and dry cleaning	77.25 (15.39)
expenditures food 2017 baby food expenditures miscellaneous 2017 coin operated apparel laundry and dry cleaning	196.39 (41.19)
expenditures home 2017 telephones and accessories expenditures miscellaneous 2017 coin operated apparel laundry and dry cleaning	314.84 (96.13)
expenditures home 2017 lawn and garden equipment expenditures miscellaneous 2017 coin operated apparel laundry and dry cleaning	327.61 (77.22)
expenditures home 2017 office furniture for home use expenditures miscellaneous 2017 coin operated apparel laundry and dry cleaning	43.61 (15.70)
expenditures home 2017 mens sportcoats tailored jackets expenditures miscellaneous 2017 coin operated apparel laundry and dry cleaning	48.83 (15.46)
expenditures home 2017 womens sportcoats tailored jackets expenditures miscellaneous 2017 coin operated apparel laundry and dry cleaning	52.74 (21.39)
expenditures home 2017 rent expenditures miscellaneous 2017 coin operated apparel laundry and dry cleaning	19062.75 (9193.11)
expenditures home 2017 rent as pay expenditures miscellaneous 2017 coin operated apparel laundry and dry cleaning	485.40 (256.49)
expenditures home 2017 maintenance insurance and other expenses rented expenditures miscellaneous 2017 coin operated apparel laundry and dry cleaning	609.61 (166.28)
expenditures home 2017 electricity expenditures miscellaneous 2017 coin operated apparel laundry and dry cleaning	8168.55 (2053.92)
expenditures home 2017 housekeeping services expenditures miscellaneous 2017 coin operated apparel laundry and dry cleaning	961.52 (304.35)
expenditures home 2017 coin operated household laundry and dry cleaning nonclothing expenditures miscellaneous 2017 coin operated apparel laundry and dry cleaning	55.87 (28.45)
expenditures home 2017 home security system service fee expenditures miscellaneous 2017 coin operated apparel laundry and dry cleaning	170.91 (43.60)
expenditures home 2017 rental of televisions expenditures miscellaneous 2017 coin operated apparel laundry and dry cleaning	3.34 (3.35)
expenditures home 2017 boys uniforms and active sportswear expenditures miscellaneous 2017 coin operated apparel laundry and dry cleaning	92.32 (19.89)
expenditures home 2017 maintenance and repair services rented expenditures miscellaneous 2017 coin operated apparel laundry and dry cleaning	214.60 (58.99)
expenditures home 2017 womens hosiery expenditures miscellaneous 2017 coin operated apparel laundry and dry cleaning	128.41 (27.17)
expenditures home 2017 girls coats and jackets expenditures miscellaneous 2017 coin operated apparel laundry and dry cleaning	31.87 (6.98)

expenditures home 2017 girls footwear expenditures miscellaneous 2017 coin operated apparel laundry and dry cleaning	235.72 (57.57)
expenditures miscellaneous 2017 auto truck rental expenditures miscellaneous 2017 coin operated apparel laundry and dry cleaning	255.28 (65.58)
expenditures miscellaneous 2017 finance late interest charges for student loans expenditures miscellaneous 2017 coin operated apparel laundry and dry cleaning	882.47 (229.52)
expenditures miscellaneous 2017 gas tank repair replacement expenditures miscellaneous 2017 coin operated apparel laundry and dry cleaning	71.16 (16.47)
expenditures miscellaneous 2017 newspapers expenditures miscellaneous 2017 coin operated apparel laundry and dry cleaning	0.23 (0.05)
expenditures miscellaneous 2017 value surrender of whole life insurance policy expenditures miscellaneous 2017 coin operated apparel laundry and dry cleaning	47120.16 (15387.72)
expenditures miscellaneous 2017 watches expenditures miscellaneous 2017 coin operated apparel laundry and dry cleaning	397.39 (164.59)
expenditures miscellaneous 2017 watch and jewelry repair expenditures miscellaneous 2017 coin operated apparel laundry and dry cleaning	172.63 (84.99)
expenditures miscellaneous 2017 apparel laundry and dry cleaning not coin operated expenditures miscellaneous 2017 coin operated apparel laundry and dry cleaning	266.66 (86.03)
expenditures miscellaneous 2017 rented vehicles expenditures miscellaneous 2017 coin operated apparel laundry and dry cleaning	394.40 (98.44)
expenditures miscellaneous 2017 towing charges expenditures miscellaneous 2017 coin operated apparel laundry and dry cleaning	23.95 (7.20)
expenditures miscellaneous 2017 public and other transportation expenditures miscellaneous 2017 coin operated apparel laundry and dry cleaning	4050.25 (1195.95)
expenditures miscellaneous 2017 intracity mass transit fares expenditures miscellaneous 2017 coin operated apparel laundry and dry cleaning	515.43 (190.84)
expenditures miscellaneous 2017 school bus expenditures miscellaneous 2017 coin operated apparel laundry and dry cleaning	39.99 (22.24)
expenditures miscellaneous 2017 rental of supportive convalescent medical equipment expenditures miscellaneous 2017 coin operated apparel laundry and dry cleaning	3.17 (0.74)
expenditures miscellaneous 2017 global positioning system devices expenditures miscellaneous 2017 coin operated apparel laundry and dry cleaning	6.81 (7.39)
expenditures miscellaneous 2017 photographic equipment expenditures miscellaneous 2017 coin operated apparel laundry and dry cleaning	105.22 (28.97)
expenditures miscellaneous 2017 wigs and hairpieces expenditures miscellaneous 2017 coin operated apparel laundry and dry cleaning	14.24 (15.39)
expenditures miscellaneous 2017 vacation clubs expenditures miscellaneous 2017 coin operated apparel laundry and dry cleaning	171.30 (86.03)
expenditures miscellaneous 2017 child support expenditures expenditures miscellaneous 2017 coin operated apparel laundry and dry cleaning	1222.24 (264.80)
expenditures miscellaneous 2017 cash contribution to educational institutions expenditures miscellaneous 2017 coin operated apparel laundry and dry cleaning	226.77 (85.28)
Hospital Expenditures (mean(SD))	
expenditures food 2017 lunch at vending machines and mobile vendors expenditures miscellaneous 2017 coin operated apparel laundry and dry cleaning	77.72 (15.55)
expenditures food 2017 dinner at employer and school cafeterias expenditures miscellaneous 2017 coin operated apparel laundry and dry cleaning	34.81 (8.09)

expenditures food 2017 breakfast and brunch at full service restaurants expenditures miscellaneous 2017 coin operated apparel laundry and dry cleaning	705.35 (139.62)
expenditures food 2017 education expenditures miscellaneous 2017 coin operated apparel laundry and dry cleaning	8667.61 (1985.91)
expenditures food 2017 college tuition expenditures miscellaneous 2017 coin operated apparel laundry and dry cleaning	5254.71 (1272.63)
expenditures food 2017 test preparation tutoring services expenditures miscellaneous 2017 coin operated apparel laundry and dry cleaning	111.37 (38.17)
expenditures food 2017 other schools tuition expenditures miscellaneous 2017 coin operated apparel laundry and dry cleaning	54.11 (14.73)
expenditures food 2017 other school expenses including rentals expenditures miscellaneous 2017 coin operated apparel laundry and dry cleaning	282.00 (60.72)
expenditures food 2017 school books supplies equipment for elementary high school expenditures miscellaneous 2017 coin operated apparel laundry and dry cleaning	101.22 (21.86)
expenditures food 2017 school lunches expenditures miscellaneous 2017 coin operated apparel laundry and dry cleaning	376.04 (82.27)
expenditures food 2017 meals as pay expenditures miscellaneous 2017 coin operated apparel laundry and dry cleaning	161.31 (44.87)
expenditures food 2017 wine expenditures miscellaneous 2017 coin operated apparel laundry and dry cleaning	809.71 (178.76)
expenditures food 2017 alcoholic beverages purchased on trips expenditures miscellaneous 2017 coin operated apparel laundry and dry cleaning	366.36 (84.19)
expenditures food 2017 fresh fish and shellfish expenditures miscellaneous 2017 coin operated apparel laundry and dry cleaning	381.41 (81.02)
expenditures food 2017 artificial sweeteners expenditures miscellaneous 2017 coin operated apparel laundry and dry cleaning	24.82 (5.18)
expenditures food 2017 jams preserves other sweets expenditures miscellaneous 2017 coin operated apparel laundry and dry cleaning	173.85 (35.32)
expenditures food 2017 fats and oils expenditures miscellaneous 2017 coin operated apparel laundry and dry cleaning	204.74 (42.74)
expenditures food 2017 prepared desserts expenditures miscellaneous 2017 coin operated apparel laundry and dry cleaning	77.25 (15.39)
expenditures food 2017 baby food expenditures miscellaneous 2017 coin operated apparel laundry and dry cleaning	196.39 (41.19)
expenditures home 2017 telephones and accessories expenditures miscellaneous 2017 coin operated apparel laundry and dry cleaning	314.84 (96.13)
expenditures home 2017 lawn and garden equipment expenditures miscellaneous 2017 coin operated apparel laundry and dry cleaning	327.61 (77.22)
expenditures home 2017 office furniture for home use expenditures miscellaneous 2017 coin operated apparel laundry and dry cleaning	43.61 (15.70)
expenditures home 2017 mens sportcoats tailored jackets expenditures miscellaneous 2017 coin operated apparel laundry and dry cleaning	48.83 (15.46)
expenditures home 2017 womens sportcoats tailored jackets expenditures miscellaneous 2017 coin operated apparel laundry and dry cleaning	52.74 (21.39)
expenditures home 2017 rent expenditures miscellaneous 2017 coin operated apparel laundry and dry cleaning	19062.75 (9193.11)
expenditures home 2017 rent as pay expenditures miscellaneous 2017 coin operated apparel laundry and dry cleaning	485.40 (256.49)
expenditures home 2017 maintenance insurance and other expenses rented expenditures miscellaneous 2017 coin operated apparel laundry and dry cleaning	609.61 (166.28)

expenditures home 2017 electricity expenditures miscellaneous 2017 coin operated apparel laundry and dry cleaning	8168.55 (2053.92)
expenditures home 2017 housekeeping services expenditures miscellaneous 2017 coin operated apparel laundry and dry cleaning	961.52 (304.35)
expenditures home 2017 coin operated household laundry and dry cleaning nonclothing expenditures miscellaneous 2017 coin operated apparel laundry and dry cleaning	55.87 (28.45)
expenditures home 2017 home security system service fee expenditures miscellaneous 2017 coin operated apparel laundry and dry cleaning	170.91 (43.60)
expenditures home 2017 rental of televisions expenditures miscellaneous 2017 coin operated apparel laundry and dry cleaning	3.34 (3.35)
expenditures home 2017 boys uniforms and active sportswear expenditures miscellaneous 2017 coin operated apparel laundry and dry cleaning	92.32 (19.89)
expenditures home 2017 maintenance and repair services rented expenditures miscellaneous 2017 coin operated apparel laundry and dry cleaning	214.60 (58.99)
expenditures home 2017 womens hosiery expenditures miscellaneous 2017 coin operated apparel laundry and dry cleaning	128.41 (27.17)
expenditures home 2017 girls coats and jackets expenditures miscellaneous 2017 coin operated apparel laundry and dry cleaning	31.87 (6.98)
expenditures home 2017 girls footwear expenditures miscellaneous 2017 coin operated apparel laundry and dry cleaning	235.72 (57.57)
expenditures miscellaneous 2017 auto truck rental expenditures miscellaneous 2017 coin operated apparel laundry and dry cleaning	255.28 (65.58)
expenditures miscellaneous 2017 finance late interest charges for student loans expenditures miscellaneous 2017 coin operated apparel laundry and dry cleaning	882.47 (229.52)
expenditures miscellaneous 2017 gas tank repair replacement expenditures miscellaneous 2017 coin operated apparel laundry and dry cleaning	71.16 (16.47)
expenditures miscellaneous 2017 newspapers expenditures miscellaneous 2017 coin operated apparel laundry and dry cleaning	0.23 (0.05)
expenditures miscellaneous 2017 value surrender of whole life insurance policy expenditures miscellaneous 2017 coin operated apparel laundry and dry cleaning	47120.16 (15387.72)
expenditures miscellaneous 2017 watches expenditures miscellaneous 2017 coin operated apparel laundry and dry cleaning	397.39 (164.59)
expenditures miscellaneous 2017 watch and jewelry repair expenditures miscellaneous 2017 coin operated apparel laundry and dry cleaning	172.63 (84.99)
expenditures miscellaneous 2017 apparel laundry and dry cleaning not coin operated expenditures miscellaneous 2017 coin operated apparel laundry and dry cleaning	266.66 (86.03)
expenditures miscellaneous 2017 rented vehicles expenditures miscellaneous 2017 coin operated apparel laundry and dry cleaning	394.40 (98.44)
expenditures miscellaneous 2017 towing charges expenditures miscellaneous 2017 coin operated apparel laundry and dry cleaning	23.95 (7.20)
expenditures miscellaneous 2017 public and other transportation expenditures miscellaneous 2017 coin operated apparel laundry and dry cleaning	4050.25 (1195.95)
expenditures miscellaneous 2017 intracity mass transit fares expenditures miscellaneous 2017 coin operated apparel laundry and dry cleaning	515.43 (190.84)
expenditures miscellaneous 2017 school bus expenditures miscellaneous 2017 coin operated apparel laundry and dry cleaning	39.99 (22.24)
expenditures miscellaneous 2017 rental of supportive convalescent medical equipment expenditures miscellaneous 2017 coin operated apparel laundry and dry cleaning	3.17 (0.74)

expenditures miscellaneous 2017 global positioning system devices expenditures miscellaneous 2017 coin operated apparel laundry and dry cleaning	6.81 (7.39)
expenditures miscellaneous 2017 photographic equipment expenditures miscellaneous 2017 coin operated apparel laundry and dry cleaning	105.22 (28.97)
expenditures miscellaneous 2017 wigs and hairpieces expenditures miscellaneous 2017 coin operated apparel laundry and dry cleaning	14.24 (15.39)
expenditures miscellaneous 2017 vacation clubs expenditures miscellaneous 2017 coin operated apparel laundry and dry cleaning	171.30 (86.03)
expenditures miscellaneous 2017 child support expenditures expenditures miscellaneous 2017 coin operated apparel laundry and dry cleaning	1222.24 (264.80)
expenditures miscellaneous 2017 cash contribution to educational institutions expenditures miscellaneous 2017 coin operated apparel laundry and dry cleaning	226.77 (85.28)

HH=Household
Fam=Family
Pop=Population
Non Fam=Non family
OT=Other
ER=Emergency room
RV=recreational vehicle
Equip=equipment
Misc.=miscellaneous
BCBS=Blue Cross Blue Shield
OOT=Out of town
RIHC=resource intensive healthcare
