# Supplementary material for: Can diverse population characteristics be leveraged in a machine learning pipeline to predict resource intensive healthcare utilization among hospital service areas?
Source: BMC Health Serv Res. 2022 Jun 30;22:847. doi: 10.1186/s12913-022-08154-4 (PMC9248096; doi:10.1186/s12913-022-08154-4)

Additional File 10. QQ Plots for Predicted and Actual Outcome Values from Machine Learning Prediction Models

- Additional File 10
  - File format: PDF
  - File title: QQ Plots for Predicted and Actual Outcome Values from Machine Learning Prediction Models
  - File description: Additional figure to complement prediction model results

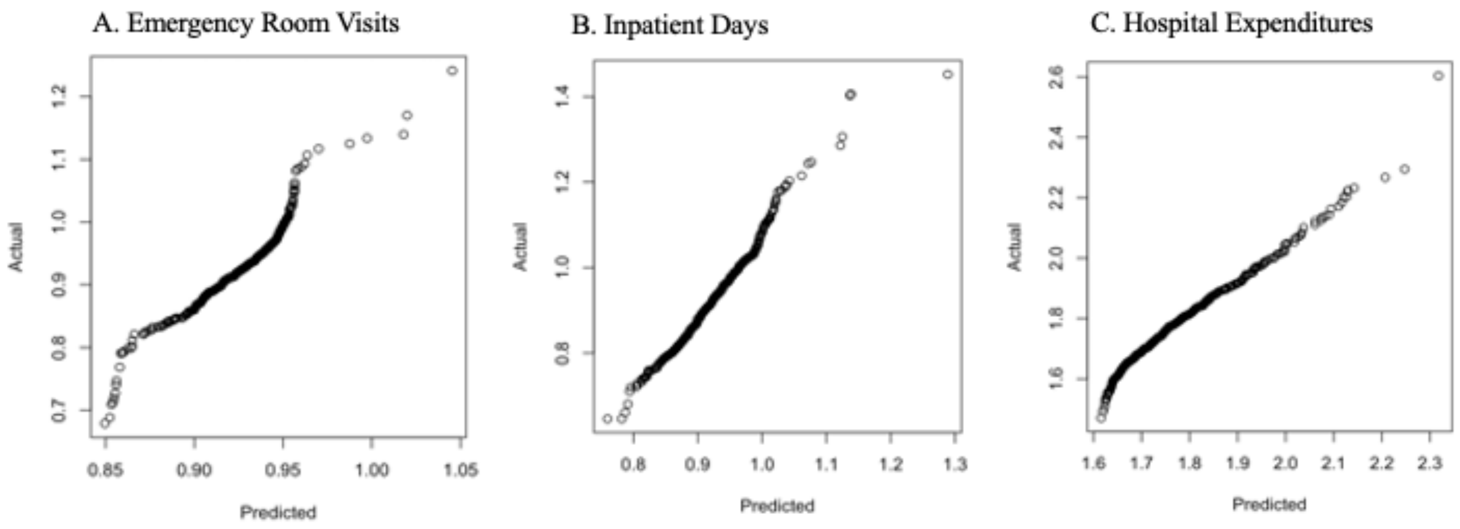

Supplement: Supplementary file 10 — Additional file 10. [file 12913_2022_8154_MOESM10_ESM.pdf]
