# Supplementary material for: Can diverse population characteristics be leveraged in a machine learning pipeline to predict resource intensive healthcare utilization among hospital service areas?
Source: BMC Health Serv Res. 2022 Jun 30;22:847. doi: 10.1186/s12913-022-08154-4 (PMC9248096; doi:10.1186/s12913-022-08154-4)

Additional File 11. QQ Plots with Orthonormal Scale for Predicted and Actual Outcome Values from Machine Learning Prediction Models

- Additional File 11
    - File format: PDF
    - File title: QQ Plots with Orthonormal Scale for Predicted and Actual Outcome Values from Machine Learning Prediction Models
    - File description: Additional figure to complement prediction model results with an orthonormal scale

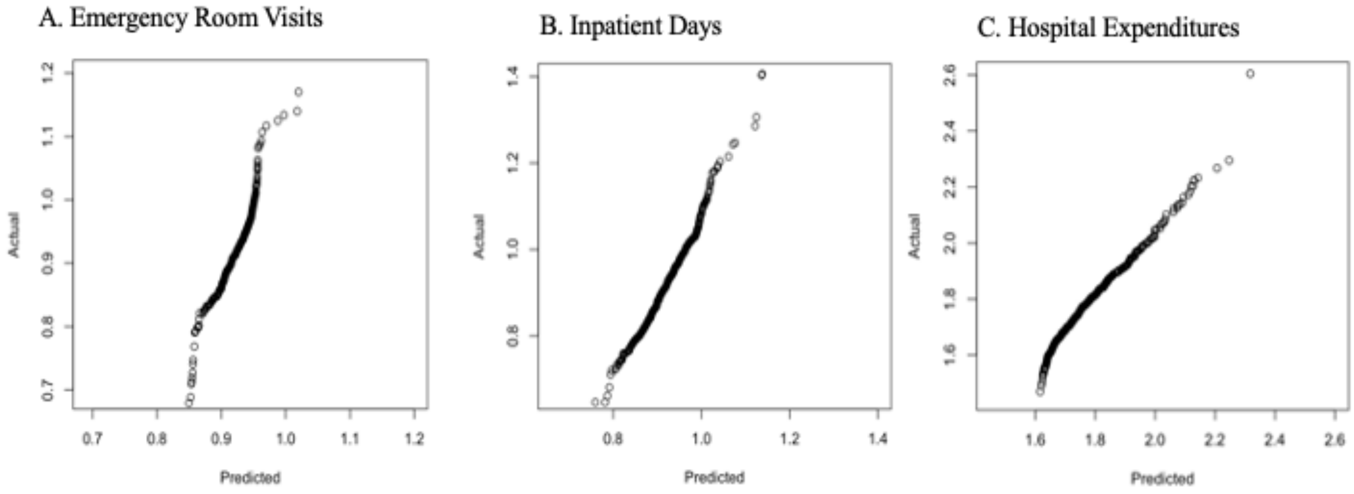

Supplement: Supplementary file 11 — Additional file 11. [file 12913_2022_8154_MOESM11_ESM.pdf]
