# Supplementary material for: Can diverse population characteristics be leveraged in a machine learning pipeline to predict resource intensive healthcare utilization among hospital service areas?
Source: BMC Health Serv Res. 2022 Jun 30;22:847. doi: 10.1186/s12913-022-08154-4 (PMC9248096; doi:10.1186/s12913-022-08154-4)
Supplement: Supplementary file 12 — Additional file 12. [file 12913_2022_8154_MOESM12_ESM.pdf]

Additional File 12. Variable Importance from Best Performing Machine Learning Model for Log ER Visits per Capita

- Additional File 12
  - File format: PDF
  - File title: Variable Importance from Best Performing Machine Learning Model for Log ER Visits per Capita
  - File description: Long table, prediction model output for log ER visits per capita

	Variable Importance
census employment 2017 employment civilian males pop 16 persons	12.278
census employment 2017 employment agriculture forestry fishing and hunting pop 16 persons	10.825
census demographics 2017 families married families	10.290
census demographics 2017 family head of household female households	10.009
expenditures miscellaneous 2017 adult diapers total amount	9.732
census demographics 2017 households with no vehicles households	9.477
health children 2017 hay fever count of households persons	9.440
census demographics 2017 family head of household male households	9.379
census housing units 2017 housing owner households valued 25000 29999 count housing units	8.617
census employment 2017 employment work at home empl 16 persons	8.501
census employment 2017 employment self employed workers in own not incorporated business pop 16 persons	8.467
expenditures home 2017 ground rent total amount	8.224
census demographics 2017 non families 7 or more person households	8.167
health children 2017 all persons with a usual place of health care emergency room count of households persons	7.398
health children 2017 skin allergies count of households persons	7.264
census housing units 2017 housing built 1940 to 1949 count housing units	7.257
health children 2017 emergency room visits in past 12 months for children under 18 one count of households persons	7.242
expenditures miscellaneous 2017 gift to non cu members of stocks bonds and mutual funds total amount	7.211
census employment 2017 employment travel time 30 59 min empl 16 persons	7.158
health children 2017 all persons with a usual place of health care hospital outpatient count of households persons	7.075
health children 2017 respiratory allergies count of households persons	6.879
census demographics 2017 non families aged 75 years and over households	6.854
health children 2017 children 2 17 years more than 2 years but not more than 5 years ago since last dental visit count of households persons	6.776

census demographics 2017 families 3 person families	6.752
health children 2017 children receiving special education or early intervention services count of households persons	6.685
census housing units 2017 housing median year moved in count year census housing units 2017 housing median year built count year	6.484
expenditures food 2017 college tuition total amount	6.468
health children 2017 all persons with a usual place of health care some other place count of households persons	6.468
census demographics 2017 household income average	6.308
census housing units 2017 housing median rent count census housing units 2017 housing median year built count year	6.151
health children 2017 number school days missed in past 12 months due to illness or injury aged 5 17 6 10 days count of households persons	6.124
census employment 2017 employment walked to work empl 16 persons	6.119
health children 2017 prescription medication taken regularly for at least 3 months count of households persons	6.086
census demographics 2017 households median size number persons	6.025
census demographics 2017 households with 4 vehicles households	5.974
census employment 2017 employment travel time 15 29 min empl 16 persons	5.953
census housing units 2017 housing rent 500 749 count housing units	5.947
census employment 2017 employment private not for profit wage and salary workers pop 16 persons	5.826
health children 2017 very good health status respondent assessed count of households persons	5.788
health children 2017 food allergies count of households persons	5.749
census employment 2017 employment not in the labor force female pop 16 persons	5.721
expenditures miscellaneous 2017 rental of medical equipment total amount	5.683
census demographics 2017 asian population alone persons	5.649
census housing units 2017 housing owner households valued 40000 49999 count housing units	5.592
census housing units 2017 housing owner households valued 150000 174999 count housing units	5.590
census housing units 2017 housing owner households valued 20000 24999 count housing units	5.575
health children 2017 children 2 17 years more than 1 year but not more than 2 years since last dental visit count of households persons	5.569
expenditures home 2017 infant nightwear loungewear total amount	5.551
census housing units 2017 housing rent 1250 1499 count housing units	5.516
census employment 2017 employment accommodation and food services pop 16 persons	5.500
census employment 2017 occupation professional and related pop 16 persons	5.465
health children 2017 children 3 to 17 learning disability count of households persons	5.458
census demographics 2017 households with income 15000 to 24999 households	5.431

census employment 2017 employment private for profit wage and salary workers self pop 16 persons	5.418
census housing units 2017 housing median rent count	5.413
health children 2017 last health care professional visit 6 months or less count of households persons	5.398
census demographics 2017 population citizenship native persons	5.334
census housing units 2017 housing built 1950 to 1959 count housing units	5.333
expenditures home 2017 infants equipment total amount	5.286
health children 2017 number school days missed in past 12 months due to illness or injury aged 5 17 3 5 days count of households persons	5.270
expenditures miscellaneous 2017 photographic equipment total amount	5.210
census demographics 2017 education enrolled private grades 5 8 pop 3 persons	5.186
health children 2017 children 2 17 years more than 6 months but less than 1 year since last dental visit count of households persons	5.180
health adults 2017 full guidelines strengthening and aerobic combined met both muscle strengthening and aerobic guidelines count of households persons	5.137
census demographics 2017 families married with children under 18 families	5.131
census demographics 2017 family median size number persons	5.116
census housing units 2017 housing rent less than 250 count housing units	5.103
expenditures miscellaneous 2017 day care centers nursery and preschools total amount	5.061
census employment 2017 employment unemployed female pop 16 persons	5.006
census demographics 2017 education attainment professional degree pop 25 persons	5.001
census employment 2017 occupation construction extraction and maintenance pop 16 persons	4.982
health children 2017 last health care professional visit more than 1 year but not more than 2 years ago count of households persons	4.944
health adults 2017 alcohol current infrequent count of households persons	4.942
health adults 2017 type some other place count of households persons	4.928
census demographics 2017 families aged under 25 years families	4.902
census housing units 2017 housing structure with 10 19 units count housing units	4.884
health adults 2017 last doctor visit more than 2 years but less than 5 years ago count of households persons	4.865
expenditures home 2017 boys uniforms and active sportswear total amount	4.844
health children 2017 number school days missed in past 12 months due to illness or injury aged 5 17 11 or more days count of households persons	4.814
health children 2017 children 2 17 years less than 6 months since last dental visit count of households persons	4.803
census housing units 2017 housing structure with 50 units count housing units	4.795
census housing units 2017 housing structure with 5 9 units count housing units	4.755
census housing units 2017 housing built 2000 to 2009 count housing units	4.753

census demographics 2017 non families 5 person households	4.687
expenditures miscellaneous 2017 shoe repair and other shoe service total amount	4.664
health children 2017 unmet medical need count of households persons	4.649
census housing units 2017 housing owner households valued less than 10000 count housing units	4.649
expenditures home 2017 boys hosiery total amount	4.583
census demographics 2017 education enrolled public graduate or professional school pop 3 persons	4.582
census demographics 2017 two or more races head of households households	4.566
census demographics 2017 population citizenship foreign born not a citizen persons	4.558
census demographics 2017 households 3 person households	4.556
expenditures home 2017 applications games ringtones for handheld devices total amount	4.542
census demographics 2017 two or more races population persons	4.533
health adults 2017 last dental visit more than 1 year but not more than 2 years ago count of households persons	4.484
census housing units 2017 housing year moved in 1970 to 1979 count housing units	4.433
census demographics 2017 american indian and alaska native head of households households	4.430
census demographics 2017 households with income less than 15000 households	4.423
health adults 2017 some day smokers count of households persons	4.410
health adults 2017 all persons without a usual place of health care count of households persons	4.401
health children 2017 delayed care due to cost count of households persons	4.389
census demographics 2017 householder aged 35 to 44 years households	4.378
health adults 2017 stroke count of households persons	4.371
expenditures food 2017 food or board at school total amount	4.357
census housing units 2017 housing year moved in 2000 to 2009 count housing units	4.356
health children 2017 emergency room visits in past 12 months for children under 18 two or more count of households persons	4.331
health adults 2017 hay fever count of households persons	4.316
census employment 2017 employment unpaid family workers pop 16 persons	4.299
health adults 2017 liver disease count of households persons	4.277
census employment 2017 occupation service pop 16 persons	4.249
health children 2017 all persons with a usual place of health care clinic count of households persons	4.227
expenditures miscellaneous 2017 rental of supportive convalescent medical equipment total amount	4.220
health children 2017 last health care professional visit more than six months but less than 1 year count of households persons	4.185
census housing units 2017 housing built 1990 to 1999 count housing units	4.172
census demographics 2017 population institutional group quarters persons	4.172

health children 2017 number school days missed in past 12 months due to illness or injury aged 5 17 1 2 days count of households persons	4.083
health children 2017 children 2 17 years more than 5 years since last dental visit count of households persons	3.983
census housing units 2017 housing rent 750 999 count housing units	3.981
health children 2017 all persons without a usual place of health care count of households persons	3.961
census employment 2017 employment bicycle to work empl 16 persons	3.909
expenditures home 2017 mens nightwear total amount	3.903
health children 2017 number school days missed in past 12 months due to illness or injury aged 5 17 did not go to school count of households persons	3.847
health children 2017 children 2 17 years no unmet dental need count of households persons	3.837
census demographics 2017 non families aged under 25 years households	3.830
health children 2017 children 2 17 years yes unmet dental need count of households persons	3.830
census employment 2017 employment blue collar pop 16 persons	3.828
census demographics 2017 population non institutional group quarters persons	3.772
census demographics 2017 non families aged 55 to 64 years households	3.701
health adults 2017 last doctor visit never count of households persons	3.694
health children 2017 last health care professional visit more than 2 years but less than 5 years ago count of households persons	3.677
census demographics 2017 education not enrolled in school pop 3 persons	3.665
expenditures food 2017 cereals and cereal products total amount	3.664
census demographics 2017 other race population alone persons	3.623
census demographics 2017 education attainment high school pop 25 persons 2	3.606
census employment 2017 employment local government workers pop 16 persons	3.602
census employment 2017 employment health care and social assistance pop 16 persons	3.594
health adults 2017 all current smokers count of households persons	3.590
census demographics 2017 education enrolled private preprimary pop 3 persons	3.578
census employment 2017 employment real estate and rental and leasing pop 16 persons	3.569
census demographics 2017 non family households female householder with people under 18 households	3.492
census employment 2017 employment unemployed males pop 16 persons	3.469
census demographics 2017 population urban persons	3.460
census housing units 2017 home heating fuel wood count housing units	3.406
health adults 2017 last dental visit more than 2 years ago but not more than 5 years ago count of households persons	3.400
census demographics 2017 other families female householder no husband present with no children under 18 other families	3.313
health adults 2017 last doctor visit more than 1 year but not more than 2 years ago count of households persons	3.306

health adults 2017 worthlessness all or most of the time count of households persons	3.301
census demographics 2017 asian households households	3.292
census demographics 2017 american indian and alaska native population alone persons	3.231
census demographics 2017 non families aged 45 to 54 years households	3.204
health adults 2017 pain in face or jaw count of households persons	3.182
health children 2017 all persons with a usual place of health care count of households persons	3.171
census housing units 2017 housing owner households with mortgage any count households	3.142
census housing units 2017 housing owner households valued 90000 99999 count housing units	3.097
census housing units 2017 housing rent 1500 1999 count housing units	3.080
census employment 2017 employment car truck van to work carpool empl 16 persons	3.066
census housing units 2017 home heating fuel utility gas count housing units	3.037
census housing units 2017 housing year moved in 1990 to 1999 count housing units	3.015
census demographics 2017 families aged 55 to 64 years families	2.994
census housing units 2017 housing owner households valued more than 1000000 count housing units	2.974
health children 2017 fair of poor health status respondent assessed count of households persons	2.964
census housing units 2017 housing structure with 2 units count housing units	2.951
census housing units 2017 housing renter occupied count housing units	2.950
census demographics 2017 education enrolled public grades 9 12 pop 3 persons	2.949
expenditures home 2017 girls uniforms total amount	2.936
census housing units 2017 housing owner households valued 60000 69999 count housing units	2.925
census demographics 2017 black population alone persons	2.898
census employment 2017 employment administrative and support and waste mgt services pop 16 persons	2.867
expenditures food 2017 bread and cracker products total amount	2.816
health children 2017 good health status respondent assessed count of households persons	2.790
expenditures food 2017 school books supplies equipment for college total amount	2.730
census employment 2017 employment travel time 90 min empl 16 persons	2.657
expenditures home 2017 infant coat jacket snowsuit total amount	2.645
expenditures food 2017 snacks and nonalcoholic beverages at full service restaurants total amount	2.603
census demographics 2017 population speaks other language pop 5 persons	2.598
census demographics 2017 non families aged 65 to 74 years households	2.534
census employment 2017 employment other transportation to work empl 16 persons	2.532
census demographics 2017 veterans total persons	2.475
expenditures home 2017 repair of tv radio and sound equipment total amount	2.463

census housing units 2017 housing owner households valued 80000 89999 count housing units	2.449
census employment 2017 employment railroad to work empl 16 persons	2.332
census employment 2017 employment motorcycle to work empl 16 persons	2.332
census demographics 2017 education enrolled private graduate or professional school pop 3 persons	2.296
expenditures home 2017 computer installation total amount	2.277
census housing units 2017 housing built 1980 to 1989 count housing units	2.217
census employment 2017 employment armed forces male pop 16 persons	2.194
census employment 2017 employment public administration pop 16 persons	2.157
census employment 2017 employment potential pop 16 persons	2.119
census demographics 2017 education enrolled private kindergarten pop 3 persons	2.094
census demographics 2017 education enrolled private grades 1 4 pop 3 persons	2.092
census housing units 2017 housing built 1970 to 1979 count housing units	2.072
census employment 2017 employment management of companies and enterprises pop 16 persons	1.855
census employment 2017 employment construction pop 16 persons	1.756
census employment 2017 employment taxi to work empl 16 persons	1.673
census housing units 2017 housing structure with 3 4 units count housing units	1.558
census housing units 2017 housing vacant units for migrant workers count housing units	1.365
health children 2017 last health care professional visit more than 5 years count of households persons	1.229
census housing units 2017 housing vacant units rented not occupied count housing units	1.114
census housing units 2017 housing structure with 1 unit attached count housing units	1.047
expenditures food 2017 school books supplies equipment for day care nursery total amount	1.042
census employment 2017 employment state government workers pop 16 persons	0.647
census housing units 2017 housing vacant units for rent count housing units	0.497
census housing units 2017 home heating fuel no fuel used count housing units	0.414
census demographics 2017 education enrolled public preprimary pop 3 persons	0.188
census housing units 2017 housing structure boat rv van other count housing units	0.133
census employment 2017 employment federal government workers pop 16 persons	-1.140

HH=Household
Fam=Family
Pop=Population
Non Fam=Non family
OT=Other
ER=Emergency room
RV=recreational vehicle
Equip=equipment

Misc.=miscellaneous
BCBS=Blue Cross Blue Shield
OOT=Out of town
RIHC=resource intensive healthcare
