# Supplementary material for: Can diverse population characteristics be leveraged in a machine learning pipeline to predict resource intensive healthcare utilization among hospital service areas?
Source: BMC Health Serv Res. 2022 Jun 30;22:847. doi: 10.1186/s12913-022-08154-4 (PMC9248096; doi:10.1186/s12913-022-08154-4)
Supplement: Supplementary file 13 — Additional file 13. [file 12913_2022_8154_MOESM13_ESM.pdf]

Additional File 13. Coefficient Output from Best Performing Machine Learning Model for log Inpatient Days

- Additional File 13
    - File format: PDF
    - File title: Coefficient Output from Best Performing Machine Learning Model for log Inpatient Days
    - File description: Long table, prediction model output for log inpatient days per capita

	Coefficient
Intercept	-1.435
census employment 2017 employment public transportation to work empl 16 persons	0.001
census employment 2017 employment streetcar or trolley car to work empl 16 persons	0.038
census employment 2017 employment railroad to work empl 16 persons	0.003
census employment 2017 employment taxi to work empl 16 persons	0.002
census employment 2017 employment bicycle to work empl 16 persons	-0.011
census employment 2017 employment other transportation to work empl 16 persons	0.004
census employment 2017 employment travel time 15 29 min empl 16 persons	0.003
census employment 2017 employment travel time 30 59 min empl 16 persons	-0.001
census employment 2017 employment potential pop 16 persons	0.023
census employment 2017 employment unemployed female pop 16 persons	0.002
census employment 2017 employment mining quarrying and oil and gas extraction pop 16 persons	-0.002
census employment 2017 employment construction pop 16 persons	-0.005
census employment 2017 employment manufacturing pop 16 persons	-0.002
census employment 2017 employment wholesale trade pop 16 persons	0.006
census employment 2017 employment management of companies and enterprises pop 16 persons	0.027
census employment 2017 employment administrative and support and waste mgt services pop 16 persons	0.001
census employment 2017 employment health care and social assistance pop 16 persons	0.012
census employment 2017 employment arts entertainment and recreation pop 16 persons	0.004
census employment 2017 employment other services pop 16 persons	-0.004
census employment 2017 employment public administration pop 16 persons	-0.002
census employment 2017 occupation service pop 16 persons	-0.001
census employment 2017 occupation farming fishing and forestry pop 16 persons	-0.001
census employment 2017 employment private for profit wage and salary workers employee pop 16 persons	-0.001

census employment 2017 employment private for profit wage and salary workers self pop 16 persons	0.009
census employment 2017 employment private not for profit wage and salary workers pop 16 persons	0.001
census employment 2017 employment local government workers pop 16 persons	0.001
census employment 2017 employment unpaid family workers pop 16 persons	0.006
census housing units 2017 home heating fuel fuel oil kerosene etc count housing units	-0.001
census housing units 2017 home heating fuel wood count housing units	-0.001
census housing units 2017 home heating fuel solar energy count housing units	-0.007
census housing units 2017 home heating fuel other fuel count housing units	0.001
census housing units 2017 home heating fuel no fuel used count housing units	0.001
census housing units 2017 housing vacant units for rent count housing units	0.003
census housing units 2017 housing vacant units rented not occupied count housing units	-0.008
census housing units 2017 housing vacant units for sale count housing units	-0.013
census housing units 2017 housing vacant units sold not occupied count housing units	-0.003
census housing units 2017 housing vacant units for migrant workers count housing units	-0.019
census housing units 2017 housing vacant units vacant other count housing units	0.001
census housing units 2017 housing structure with 20 49 units count housing units	-0.001
census housing units 2017 housing structure with 50 units count housing units	0.001
census housing units 2017 housing structure boat rv van other count housing units	-0.022
census housing units 2017 housing rent 750 999 count housing units	0.001
census housing units 2017 housing no cash rent count housing units	-0.006
census housing units 2017 housing owner households valued less than 10000 count housing units	0.003
census housing units 2017 housing owner households valued 10000 14999 count housing units	0.003
census housing units 2017 housing owner households valued 20000 24999 count housing units	-0.007
census housing units 2017 housing owner households valued 25000 29999 count housing units	-0.004
census housing units 2017 housing owner households valued 30000 34999 count housing units	0.000
census housing units 2017 housing owner households valued 35000 39999 count housing units	-0.003
census housing units 2017 housing owner households valued 50000 59999 count housing units	0.001
census housing units 2017 housing owner households valued 250000 299999 count housing units	-0.001
census housing units 2017 housing owner households valued 750000 999999 count housing units	-0.001
census housing units 2017 housing built 1980 to 1989 count housing units	0.001
census housing units 2017 housing built 1950 to 1959 count housing units	0.001
census housing units 2017 housing year moved in 2000 to 2009 count housing units	-0.001
census housing units 2017 housing year moved in 1970 to 1979 count housing units	0.004
census housing units 2017 housing year moved in 1969 or earlier count housing units	0.004

Variables with coefficient of 0 were not included

HH=Household

Fam=Family
Pop=Population
Non Fam=Non family
OT=Other
ER=Emergency room
RV=recreational vehicle
Equip=equipment
Misc.=miscellaneous
BCBS=Blue Cross Blue Shield
OOT=Out of town
RIHC=resource intensive healthcare
