# Supplementary material for: Can diverse population characteristics be leveraged in a machine learning pipeline to predict resource intensive healthcare utilization among hospital service areas?
Source: BMC Health Serv Res. 2022 Jun 30;22:847. doi: 10.1186/s12913-022-08154-4 (PMC9248096; doi:10.1186/s12913-022-08154-4)
Supplement: Supplementary file 14 — Additional file 14. [file 12913_2022_8154_MOESM14_ESM.pdf]

Additional File 14. Relative Influence of Variables from Best Performing Machine Learning Model for log Hospital Expenditures per Capita

- Additional File 14
  - File format: PDF
  - File title: Relative Influence of Variables from Best Performing Machine Learning Model for log Hospital Expenditures per Capita
  - File description: long table, prediction model output for log hospital expenditures per capita

	Relative Influence
census demographics 2017 population urban persons	18.520
census demographics 2017 non families 6 person households	7.689
census employment 2017 employment travel time less than 15 min empl 16 persons	7.051
census demographics 2017 non families 7 or more person households	6.595
health children 2017 still have asthma count of households persons	4.387
expenditures miscellaneous 2017 global positioning system devices total amount	4.355
census demographics 2017 head of household male households	4.322
census employment 2017 employment local government workers pop 16 persons	4.308
census housing units 2017 housing median year moved in count year	4.208
census employment 2017 employment agriculture forestry fishing and hunting pop 16 persons	3.116
census demographics 2017 household income median	2.092
health children 2017 all persons with a usual place of health care some other place count of households persons	1.929
census demographics 2017 non family households female householder with people under 18 households	1.846
census demographics 2017 education enrolled private kindergarten pop 3 persons	1.656
census housing units 2017 housing median year built count year	1.597
census housing units 2017 housing built 2000 to 2009 count housing units	1.429
health children 2017 last health care professional visit more than 1 year but not more than 2 years ago count of households persons	1.396
census employment 2017 employment motorcycle to work empl 16 persons	1.387
census demographics 2017 non families 4 person households	1.311
census employment 2017 employment retail trade pop 16 persons	1.040
expenditures miscellaneous 2017 trailer and other attachable campers total amount	0.989
census demographics 2017 non family households male householder with people under 18 households	0.957
health children 2017 children 2 17 years no unmet dental need count of households persons	0.915

census employment 2017 employment health care and social assistance pop 16 persons	0.911
census employment 2017 employment utilities pop 16 persons	0.906
census housing units 2017 housing vacant units rented not occupied count housing units	0.904
health children 2017 last health care professional visit more than 5 years count of households persons	0.892
census housing units 2017 housing owner households valued 20000 24999 count housing units	0.871
census demographics 2017 population institutional group quarters persons	0.860
health children 2017 last health care professional visit more than 2 years but less than 5 years ago count of households persons	0.830
census employment 2017 employment private not for profit wage and salary workers pop 16 persons	0.794
health adults 2017 stroke count of households persons	0.777
census demographics 2017 education enrolled public preprimary pop 3 persons	0.754
census housing units 2017 housing rent 1500 1999 count housing units	0.720
health children 2017 all persons with a usual place of health care emergency room count of households persons	0.662
census demographics 2017 household income average	0.624
census demographics 2017 householder aged 85 years and over households	0.616
census housing units 2017 housing structure with 2 units count housing units	0.603
expenditures miscellaneous 2017 termination fee for car truck lease total amount	0.536
census demographics 2017 two or more races head of households households	0.511
census demographics 2017 households 1 person households	0.469
census housing units 2017 housing owner households valued 400000 499999 count housing units	0.376
census employment 2017 employment transportation and warehousing pop 16 persons	0.358
census demographics 2017 education attainment professional degree pop 25 persons	0.344
health children 2017 number school days missed in past 12 months due to illness or injury aged 5 17 6 10 days count of households persons	0.305
census demographics 2017 asian households households	0.301
census demographics 2017 population male persons	0.277
census employment 2017 employment other transportation to work empl 16 persons	0.262
census demographics 2017 education enrolled private grades 5 8 pop 3 persons	0.260
census demographics 2017 families aged 55 to 64 years families	0.246
census demographics 2017 non family households male householder with no people under 18 households	0.215
census demographics 2017 householder aged 55 to 64 years households	0.209
census demographics 2017 families 7 or more person families	0.192
census employment 2017 employment walked to work empl 16 persons	0.165
census demographics 2017 population median age years	0.152

Variables with relative influence of 0 were not included

HH=Household
Fam=Family
Pop=Population
Non Fam=Non family
OT=Other
ER=Emergency room
RV=recreational vehicle
Equip=equipment
Misc.=miscellaneous
BCBS=Blue Cross Blue Shield
OOT=Out of town
RIHC=resource intensive healthcare
