# Supplementary material for: Can diverse population characteristics be leveraged in a machine learning pipeline to predict resource intensive healthcare utilization among hospital service areas?
Source: BMC Health Serv Res. 2022 Jun 30;22:847. doi: 10.1186/s12913-022-08154-4 (PMC9248096; doi:10.1186/s12913-022-08154-4)
Supplement: Supplementary file 15 — Additional file 15. [file 12913_2022_8154_MOESM15_ESM.pdf]

Additional File 15. Multivariable linear regression model of log Emergency Room visits per capita in Hospital Service Areas 2017 (N=3,153, adjusted R^2 0.312)

- Additional File 15
  - File format: PDF
  - File title: Multivariable linear regression model of log Emergency Room visits per capita in Hospital Service Areas 2017 (N=3,153, adjusted R^2 0.312)
  - File description: Long table, model output for regression model for ER visits per capita

Variable (expressed as per capita or per capita percent)	Coefficient	Standard Error	Z Statistics	P Value	95% Lower	95% Upper
(Intercept)	-0.869	5.319	-0.163	0.870	-11.294	9.557
census demographics 2017 household income average	0.000	0.000	-0.597	0.551	0.000	0.000
census demographics 2017 population speaks other language pop 5 persons	-0.044	0.050	-0.884	0.377	-0.142	0.054
health adults 2017 worthlessness all or most of the time	-0.457	0.169	-2.708	0.007	-0.788	-0.126
census demographics 2017 population citizenship foreign born not a citizen persons	0.001	0.001	1.353	0.176	-0.001	0.003
census demographics 2017 veterans total persons	0.000	0.001	-0.736	0.462	-0.002	0.001
census demographics 2017 population institutional group quarters persons	0.000	0.000	0.797	0.426	0.000	0.000
census demographics 2017 population non institutional group quarters persons	0.305	0.240	1.271	0.204	-0.165	0.776
census demographics 2017 householder aged 35 to 44 years households	-0.002	0.002	-0.797	0.426	-0.006	0.003
health adults 2017 last doctor visit more than 1 year but not more than 2 years ago	-0.273	0.135	-2.017	0.044	-0.538	-0.008
census demographics 2017 households with 4 vehicles households	-0.001	0.001	-1.292	0.196	-0.004	0.001
census demographics 2017 education enrolled public preprimary pop 3 persons	0.001	0.001	1.505	0.132	0.000	0.002
census demographics 2017 education enrolled private preprimary pop 3 persons	0.002	0.001	1.805	0.071	0.000	0.004
census demographics 2017 education enrolled private kindergarten pop 3 persons	-0.001	0.003	-0.242	0.809	-0.006	0.004
health adults 2017 stroke	-0.161	0.076	-2.109	0.035	-0.311	-0.011
census demographics 2017 education enrolled private grades 5 8 pop 3 persons	0.001	0.001	1.196	0.232	-0.001	0.003
census demographics 2017 education enrolled public grades 9 12 pop 3 persons	-0.001	0.000	-1.675	0.094	-0.001	0.000
health children 2017 without a usual place of health care	-0.151	0.038	-3.918	0.000	-0.226	-0.075

census demographics 2017 education enrolled private graduate or professional school pop 3 persons	0.002	0.001	1.667	0.096	0.000	0.004
census demographics 2017 education not enrolled in school pop 3 persons	0.000	0.000	0.428	0.668	0.000	0.000
census demographics 2017 education attainment high school pop 25 persons 2	0.000	0.000	0.125	0.901	0.000	0.001
census demographics 2017 education attainment professional degree pop 25 persons	0.003	0.002	1.695	0.090	0.000	0.006
census demographics 2017 households with income less than 15000 households	0.000	0.003	0.027	0.978	-0.006	0.006
health children 2017 unmet medical need	-0.125	0.043	-2.907	0.004	-0.209	-0.041
census demographics 2017 households 3 person households	0.005	0.006	0.860	0.390	-0.007	0.017
census demographics 2017 families married with children under 18 families	0.001	0.001	1.776	0.076	0.000	0.002
census demographics 2017 family head of household male households	0.000	0.000	0.055	0.956	-0.001	0.001
health children 2017 children 3 to 17 learning disability	-0.060	0.030	-2.015	0.044	-0.118	-0.002
census demographics 2017 other families female householder no husband present with no children under 18 other families	0.001	0.000	1.654	0.098	0.000	0.001
census demographics 2017 non family households female householder with people under 18 households	0.000	0.007	0.001	0.999	-0.014	0.014
census demographics 2017 population urban persons	0.000	0.000	0.053	0.958	0.000	0.000
census demographics 2017 black population alone persons	-0.002	0.001	-1.705	0.088	-0.005	0.000
census demographics 2017 asian population alone persons	0.002	0.002	0.739	0.460	-0.003	0.006
health children 2017 number school days missed in past 12 months due to illness or injury aged 5 17 6 10 days	-0.055	0.028	-1.957	0.050	-0.111	0.000
census demographics 2017 other race population alone persons	-0.246	0.190	-1.295	0.195	-0.619	0.126
census demographics 2017 two or more races population persons	0.000	0.000	-1.246	0.213	0.000	0.000
census demographics 2017 asian households households	-0.011	0.008	-1.336	0.182	-0.027	0.005
census demographics 2017 american indian and alaska native head of households households	-0.006	0.003	-1.703	0.089	-0.013	0.001
census demographics 2017 two or more races head of households households	-0.006	0.003	-1.890	0.059	-0.012	0.000
census demographics 2017 families 3 person families	0.000	0.002	-0.277	0.781	-0.004	0.003
census demographics 2017 non families 5 person households	0.001	0.004	0.127	0.899	-0.008	0.009
census demographics 2017 non families 7 or more person households	-0.018	0.009	-1.980	0.048	-0.036	0.000
census demographics 2017 families aged under 25 years families	0.000	0.001	0.357	0.721	-0.002	0.003
census demographics 2017 families aged 55 to 64 years families	0.000	0.001	0.117	0.907	-0.001	0.001
census demographics 2017 non families aged under 25 years households	-0.001	0.001	-1.129	0.259	-0.002	0.001

census demographics 2017 non families aged 45 to 54 years households	0.000	0.001	0.491	0.623	-0.001	0.001
census demographics 2017 non families aged 55 to 64 years households	-0.001	0.001	-1.061	0.289	-0.002	0.001
census demographics 2017 non families aged 65 to 74 years households	0.000	0.001	-0.409	0.683	-0.001	0.001
census demographics 2017 non families aged 75 years and over households	0.000	0.001	0.049	0.961	-0.001	0.001
census demographics 2017 family median size number persons	0.002	0.006	0.385	0.700	-0.009	0.013
census demographics 2017 households median size number persons	0.002	0.005	0.484	0.628	-0.007	0.012
health adults 2017 full guidelines strengthening and aerobic combined met both muscle strengthening and aerobic guidelines	-0.033	0.048	-0.685	0.494	-0.128	0.062
census employment 2017 employment potential	-0.018	0.006	-2.840	0.005	-0.030	-0.005
health adults 2017 last dental visit more than 2 years ago but not more than 5 years ago	0.150	0.145	1.033	0.302	-0.134	0.434
health adults 2017 liver disease	-0.174	0.171	-1.013	0.311	-0.510	0.162
health adults 2017 pain in face or jaw	0.130	0.167	0.777	0.437	-0.198	0.458
census demographics 2017 education enrolled private grades 1 4 pop 3 persons	-0.004	0.001	-3.732	0.000	-0.006	-0.002
census employment 2017 employment walked to work empl	-0.004	0.001	-3.736	0.000	-0.006	-0.002
health adults 2017 all current smokers	-0.126	0.103	-1.223	0.221	-0.327	0.076
health adults 2017 some day smokers	-0.021	0.168	-0.127	0.899	-0.350	0.307
health adults 2017 alcohol current infrequent	-0.085	0.060	-1.415	0.157	-0.202	0.033
health adults 2017 hay fever	0.220	0.131	1.684	0.092	-0.036	0.476
health adults 2017 without a usual place of health care	0.113	0.098	1.154	0.248	-0.079	0.306
health adults 2017 type some other place	-0.237	0.177	-1.337	0.181	-0.585	0.111
expenditures home 2017 infants equipment	-0.004	0.001	-2.817	0.005	-0.006	0.001
health adults 2017 last doctor visit more than 2 years but less than 5 years ago	-0.113	0.166	-0.682	0.495	-0.439	0.212
census employment 2017 employment agriculture forestry fishing and hunting	-0.002	0.001	-2.711	0.007	-0.004	-0.001
health children 2017 number school days missed in past 12 months due to illness or injury aged 5 17 1 2 days	-0.024	0.025	-0.968	0.333	-0.074	0.025
health children 2017 number school days missed in past 12 months due to illness or injury aged 5 17 3 5 days	0.049	0.029	1.685	0.092	-0.008	0.106
census housing units 2017 housing year moved in 1990 to 1999	-0.002	0.001	-2.903	0.004	-0.003	-0.001
health children 2017 number school days missed in past 12 months due to illness or injury aged 5 17 11 or more days	-0.026	0.028	-0.914	0.361	-0.081	0.030
health children 2017 number school days missed in past 12 months due to illness or injury aged 5 17 did not go to school	-0.001	0.003	-0.362	0.717	-0.006	0.004

health children 2017 emergency room visits in past 12 months for children under 18 one	0.014	0.031	0.466	0.641	-0.046	0.075
health children 2017 emergency room visits in past 12 months for children under 18 two or more	-0.012	0.037	-0.333	0.739	-0.085	0.061
health children 2017 children receiving special education or early intervention services	0.061	0.037	1.662	0.097	-0.011	0.133
health children 2017 hay fever	-0.024	0.038	-0.642	0.521	-0.098	0.050
health children 2017 respiratory allergies	-0.005	0.036	-0.140	0.889	-0.076	0.066
health children 2017 food allergies	0.077	0.040	1.937	0.053	-0.001	0.155
health children 2017 skin allergies	-0.034	0.028	-1.204	0.229	-0.089	0.021
census housing units 2017 housing structure with 10 19 units	-0.001	0.001	-2.129	0.033	-0.003	0.000
census employment 2017 employment travel time 30 59 min empl	-0.001	0.000	-4.340	0.000	-0.002	-0.001
health children 2017 very good health status respondent assessed	0.014	0.034	0.403	0.687	-0.054	0.081
health children 2017 good health status respondent assessed	0.053	0.030	1.730	0.084	-0.007	0.112
health children 2017 fair of poor health status respondent assessed	-0.054	0.039	-1.376	0.169	-0.132	0.023
census demographics 2017 families married families	-0.001	0.000	-2.082	0.037	-0.002	0.000
health children 2017 with a usual place of health care	0.003	0.035	0.096	0.924	-0.066	0.072
health children 2017 with a usual place of health care clinic	-0.002	0.016	-0.093	0.926	-0.033	0.030
census housing units 2017 housing structure with 1 unit attached	-0.001	0.000	-2.467	0.014	-0.001	0.000
health children 2017 with a usual place of health care hospital outpatient	-0.050	0.039	-1.305	0.192	-0.126	0.025
health children 2017 with a usual place of health care some other place	-0.052	0.040	-1.314	0.189	-0.130	0.026
health children 2017 last health care professional visit 6 months or less	-0.055	0.035	-1.573	0.116	-0.124	0.014
census housing units 2017 housing built 2000 to 2009	-0.001	0.000	-1.989	0.047	-0.001	0.000
health children 2017 last health care professional visit more than 1 year but not more than 2 years ago	0.053	0.040	1.331	0.183	-0.025	0.131
health children 2017 last health care professional visit more than 2 years but less than 5 years ago	-0.068	0.040	-1.687	0.092	-0.147	0.011
health children 2017 last health care professional visit more than 5 years	-0.042	0.039	-1.088	0.277	-0.119	0.034
census housing units 2017 housing median rent count census housing units 2017 housing median year built count year	0.000	0.000	-2.070	0.039	0.000	0.000
health children 2017 delayed care due to cost	0.043	0.037	1.162	0.245	-0.029	0.114
health children 2017 children 2 17 years yes unmet dental need	0.036	0.035	1.038	0.299	-0.032	0.103
health children 2017 children 2 17 years no unmet dental need	0.043	0.028	1.521	0.128	-0.012	0.099
health children 2017 children 2 17 years less than 6 months since last dental visit	-0.006	0.025	-0.230	0.818	-0.054	0.043

health children 2017 children 2 17 years more than 6 months but less than 1 year since last dental visit	0.025	0.032	0.783	0.434	-0.037	0.087
health children 2017 children 2 17 years more than 1 year but not more than 2 years since last dental visit	0.006	0.032	0.197	0.844	-0.056	0.069
health children 2017 children 2 17 years more than 2 years but not more than 5 years ago since last dental visit	-0.051	0.034	-1.496	0.135	-0.118	0.016
census housing units 2017 housing median rent count	0.000	0.000	2.478	0.013	0.000	0.000
census housing units 2017 housing built 1950 to 1959	0.001	0.000	2.295	0.022	0.000	0.001
census employment 2017 employment railroad to work empl	-0.002	0.002	-1.170	0.242	-0.006	0.001
census employment 2017 employment taxi to work empl	0.008	0.007	1.074	0.283	-0.006	0.022
census employment 2017 employment motorcycle to work empl	0.007	0.007	1.110	0.267	-0.006	0.020
census employment 2017 employment bicycle to work empl	-0.003	0.002	-1.143	0.253	-0.008	0.002
census housing units 2017 housing built 1990 to 1999	0.001	0.000	3.351	0.001	0.000	0.002
census employment 2017 employment other transportation to work empl	-0.001	0.002	-0.616	0.538	-0.005	0.003
census employment 2017 employment work at home empl	-0.001	0.001	-0.889	0.374	-0.003	0.001
census employment 2017 employment travel time 15 29 min empl	0.000	0.000	-0.132	0.895	-0.001	0.001
census housing units 2017 housing rent 750 999	0.001	0.001	2.058	0.040	0.000	0.002
census employment 2017 employment travel time 90 min empl	-0.001	0.001	-0.543	0.587	-0.003	0.002
census demographics 2017 education enrolled public graduate or professional school pop 3 persons	0.002	0.001	2.258	0.024	0.000	0.003
census employment 2017 employment civilian males	-0.001	0.001	-1.052	0.293	-0.003	0.001
census employment 2017 employment armed forces male	-0.001	0.001	-0.945	0.345	-0.003	0.001
census employment 2017 employment unemployed males	0.013	0.011	1.217	0.224	-0.008	0.034
census employment 2017 employment unemployed female	-0.002	0.012	-0.192	0.848	-0.027	0.022
census employment 2017 employment not in the labor force female	0.001	0.001	0.826	0.409	-0.001	0.002
census employment 2017 employment car truck van to work carpool empl	0.002	0.001	2.748	0.006	0.001	0.003
census employment 2017 employment construction	0.000	0.001	-0.096	0.924	-0.002	0.002
census employment 2017 employment real estate and rental and leasing	0.005	0.003	1.696	0.090	-0.001	0.010
census employment 2017 employment management of companies and enterprises	-0.010	0.016	-0.649	0.516	-0.041	0.021
census employment 2017 employment administrative and support and waste mgt services	0.003	0.002	1.533	0.125	-0.001	0.006
expenditures food 2017 food or board at school	0.002	0.001	2.162	0.031	0.000	0.004
census employment 2017 employment accommodation and food services	0.002	0.001	1.616	0.106	0.000	0.004

census employment 2017 employment public administration	-0.002	0.001	-1.773	0.076	-0.004	0.000
census employment 2017 occupation professional and related	0.000	0.001	0.396	0.692	-0.001	0.002
census employment 2017 occupation service	-0.001	0.001	-0.896	0.370	-0.002	0.001
census employment 2017 occupation construction extraction and maintenance	-0.001	0.001	-1.482	0.138	-0.003	0.000
census employment 2017 employment blue collar	0.000	0.001	-0.110	0.912	-0.001	0.001
census employment 2017 employment private for profit wage and salary workers self	-0.002	0.001	-1.470	0.142	-0.005	0.001
census employment 2017 employment private not for profit wage and salary workers	0.002	0.001	2.586	0.010	0.001	0.004
census employment 2017 employment local government workers	0.001	0.001	1.372	0.170	-0.001	0.003
census employment 2017 employment state government workers	0.000	0.001	-0.035	0.972	-0.002	0.002
census demographics 2017 households with no vehicles households	0.002	0.001	2.416	0.016	0.000	0.004
census employment 2017 employment self employed workers in own not incorporated business	-0.001	0.001	-0.829	0.407	-0.003	0.001
census employment 2017 employment unpaid family workers	-0.003	0.005	-0.506	0.613	-0.013	0.008
census employment 2017 employment health care and social assistance	0.003	0.001	3.282	0.001	0.001	0.004
census housing units 2017 home heating fuel utility gas	0.000	0.000	-1.107	0.268	0.000	0.000
census housing units 2017 home heating fuel wood	0.000	0.000	1.099	0.272	0.000	0.001
census housing units 2017 home heating fuel no fuel used	0.000	0.000	-0.740	0.459	-0.001	0.001
census housing units 2017 housing vacant units for rent	-0.001	0.001	-1.063	0.288	-0.003	0.001
census housing units 2017 housing vacant units rented not occupied	0.001	0.010	0.077	0.939	-0.018	0.020
census housing units 2017 housing vacant units for migrant workers	0.010	0.008	1.266	0.206	-0.006	0.026
expenditures home 2017 boys uniforms and active sportswear	0.003	0.001	2.216	0.027	0.000	0.006
census housing units 2017 housing structure with 2 units	-0.001	0.001	-1.649	0.099	-0.002	0.000
census housing units 2017 housing structure with 3 4 units	0.001	0.001	0.888	0.375	-0.001	0.002
census housing units 2017 housing structure with 5 9 units	-0.001	0.001	-1.562	0.118	-0.002	0.000
census employment 2017 employment federal government workers	0.004	0.001	3.045	0.002	0.001	0.006
census housing units 2017 housing structure with 50 units	0.001	0.001	0.831	0.406	-0.001	0.002
census housing units 2017 housing structure boat rv van other	-0.002	0.005	-0.425	0.671	-0.011	0.007
census housing units 2017 housing rent less than 250	0.000	0.001	0.085	0.933	-0.001	0.002
census housing units 2017 housing rent 500 749	0.001	0.000	1.218	0.223	0.000	0.001
census demographics 2017 american indian and alaska native population alone persons	0.004	0.002	2.584	0.010	0.001	0.007

census housing units 2017 housing rent 1250 1499	-0.001	0.001	-0.839	0.402	-0.004	0.002
census housing units 2017 housing rent 1500 1999	0.000	0.002	-0.005	0.996	-0.003	0.003
census housing units 2017 housing owner households valued less than 10000	-0.001	0.001	-0.973	0.331	-0.004	0.001
census housing units 2017 housing owner households valued 20000 24999	-0.001	0.002	-0.934	0.350	-0.005	0.002
census housing units 2017 housing owner households valued 25000 29999	-0.003	0.002	-1.768	0.077	-0.006	0.000
census housing units 2017 housing owner households valued 40000 49999	0.000	0.001	-0.398	0.690	-0.003	0.002
census housing units 2017 housing owner households valued 60000 69999	0.001	0.001	0.713	0.476	-0.001	0.002
census housing units 2017 housing owner households valued 80000 89999	0.001	0.001	1.303	0.193	-0.001	0.003
census housing units 2017 housing owner households valued 90000 99999	0.000	0.001	0.315	0.753	-0.002	0.002
census housing units 2017 housing owner households valued 150000 174999	0.001	0.001	1.923	0.055	0.000	0.002
census housing units 2017 housing owner households valued more than 1000000	-0.001	0.001	-0.847	0.397	-0.002	0.001
census demographics 2017 households with income 15000 to 24999 households	0.011	0.005	2.379	0.017	0.002	0.021
expenditures home 2017 boys hosiery	0.013	0.005	2.487	0.013	0.003	0.023
census housing units 2017 housing built 1980 to 1989	0.000	0.000	0.551	0.581	0.000	0.001
census housing units 2017 housing built 1970 to 1979	-0.001	0.000	-1.851	0.064	-0.001	0.000
health children 2017 children 2 17 years more than 5 years since last dental visit	0.059	0.024	2.450	0.014	0.012	0.105
census housing units 2017 housing built 1940 to 1949	0.001	0.001	1.443	0.149	0.000	0.002
census housing units 2017 housing year moved in 2000 to 2009	0.000	0.001	0.641	0.521	-0.001	0.001
health children 2017 prescription medication taken regularly for at least 3 months	0.071	0.033	2.161	0.031	0.007	0.136
census housing units 2017 housing year moved in 1970 to 1979	0.000	0.001	0.366	0.714	-0.001	0.002
census housing units 2017 housing renter occupied	0.000	0.000	0.204	0.838	-0.001	0.001
census housing units 2017 housing owner households with mortgage any	0.000	0.000	-0.693	0.488	-0.001	0.000
census housing units 2017 housing median year moved in count year census housing units 2017 housing median year built count year	0.000	0.000	1.754	0.080	0.000	0.000
health children 2017 with a usual place of health care emergency room	0.078	0.039	1.984	0.047	0.001	0.154
expenditures food 2017 snacks and nonalcoholic beverages at full service restaurants	0.001	0.001	0.778	0.436	-0.001	0.002
health children 2017 last health care professional visit more than six months but less than 1 year	0.089	0.037	2.373	0.018	0.015	0.162
expenditures food 2017 college tuition	0.000	0.000	-0.913	0.361	0.000	0.000
expenditures food 2017 school books supplies equipment for college	0.000	0.000	-0.150	0.881	-0.001	0.001

expenditures food 2017 school books supplies equipment for day care nursery	-0.026	0.049	-0.522	0.602	-0.123	0.071
expenditures food 2017 bread and cracker products	-0.008	0.008	-1.023	0.306	-0.024	0.008
expenditures food 2017 cereals and cereal products	0.000	0.000	-0.175	0.861	0.000	0.000
census demographics 2017 population citizenship native persons	0.149	0.067	2.232	0.026	0.018	0.280
expenditures home 2017 mens nightwear	-0.014	0.026	-0.561	0.575	-0.065	0.036
health adults 2017 last dental visit more than 1 year but not more than 2 years ago	0.175	0.087	2.021	0.043	0.005	0.345
expenditures home 2017 ground rent	0.001	0.000	1.564	0.118	0.000	0.001
expenditures home 2017 computer installation	-0.027	0.064	-0.432	0.666	-0.152	0.097
expenditures home 2017 applications games ringtones for handheld devices	0.025	0.026	0.956	0.339	-0.026	0.076
expenditures home 2017 repair of tv radio and sound equipment	-0.023	0.038	-0.612	0.540	-0.098	0.052
health adults 2017 last doctor visit never	0.659	0.192	3.441	0.001	0.284	1.034
expenditures home 2017 girls uniforms	-0.030	0.024	-1.223	0.221	-0.077	0.018
expenditures home 2017 infant coat jacket snowsuit	0.087	0.066	1.324	0.186	-0.042	0.216
expenditures home 2017 infant nightwear loungewear	0.007	0.009	0.802	0.422	-0.010	0.025
expenditures miscellaneous 2017 day care centers nursery and preschools	-0.001	0.001	-0.880	0.379	-0.002	0.001
expenditures miscellaneous 2017 shoe repair and other shoe service	0.010	0.087	0.110	0.913	-0.161	0.180
expenditures miscellaneous 2017 adult diapers	-0.102	0.074	-1.376	0.169	-0.247	0.043
expenditures miscellaneous 2017 rental of medical equipment	-0.118	0.117	-1.012	0.311	-0.347	0.111
expenditures miscellaneous 2017 rental of supportive convalescent medical equipment	-0.136	0.141	-0.961	0.337	-0.413	0.141
expenditures miscellaneous 2017 photographic equipment	0.019	0.013	1.468	0.142	-0.006	0.045
expenditures miscellaneous 2017 gift to non cu members of stocks bonds and mutual funds	0.001	0.002	0.386	0.699	-0.004	0.005

HH=Household
Fam=Family
Pop=Population
Non Fam=Non family
OT=Other
ER=Emergency room
RV=recreational vehicle
Equip=equipment
Misc.=miscellaneous
BCBS=Blue Cross Blue Shield
OOT=Out of town
RIHC=resource intensive healthcare
