# Supplementary material for: Can diverse population characteristics be leveraged in a machine learning pipeline to predict resource intensive healthcare utilization among hospital service areas?
Source: BMC Health Serv Res. 2022 Jun 30;22:847. doi: 10.1186/s12913-022-08154-4 (PMC9248096; doi:10.1186/s12913-022-08154-4)
Supplement: Supplementary file 16 — Additional file 16. [file 12913_2022_8154_MOESM16_ESM.pdf]

Additional File 16. Multiple linear regression of log Inpatient Days per capita in Hospital Service Areas (N=3,174, Adjusted R^2 0.329)

- Additional File 16
  - File format: PDF
  - File title: Multiple linear regression of log Inpatient Days per capita in Hospital Service Areas (N=3,174, Adjusted R^2 0.329)
  - File description: Long table, model output for regression model for inpatient days per capita

Variables (expressed as per capita or per capita percent)	Coefficient	Standard Error	Z Statistic	P Value	95% Lower	95% Upper
health children 2017 food allergies	-0.320	0.063	-5.083	0.000	-0.444	-0.197
census demographics 2017 population speaks other language pop 5 persons	-0.218	0.089	-2.460	0.014	-0.392	-0.044
census demographics 2017 population institutional group quarters persons	0.000	0.000	-1.378	0.168	0.000	0.000
census demographics 2017 householder aged 45 to 54 years households	0.008	0.009	0.956	0.339	-0.009	0.025
census demographics 2017 householder aged 85 years and over households	-0.013	0.007	-1.768	0.077	-0.026	0.001
census demographics 2017 households with no vehicles households	0.002	0.002	0.863	0.388	-0.002	0.006
census demographics 2017 households with 4 vehicles households	0.003	0.002	1.498	0.134	-0.001	0.007
census demographics 2017 education enrolled public or private school pop 3 persons	0.000	0.001	-0.603	0.547	-0.002	0.001
census demographics 2017 education enrolled public preprimary pop 3 persons	-0.002	0.001	-1.765	0.078	-0.005	0.000
census demographics 2017 education enrolled public kindergarten pop 3 persons	0.002	0.001	1.466	0.143	-0.001	0.005
census demographics 2017 education enrolled private kindergarten pop 3 persons	0.002	0.005	0.443	0.658	-0.007	0.011
health children 2017 last health care professional visit more than 2 years but less than 5 years ago	-0.215	0.071	-3.015	0.003	-0.356	-0.075
census demographics 2017 education enrolled private grades 1 4 pop 3 persons	-0.004	0.002	-1.793	0.073	-0.008	0.000
census demographics 2017 education enrolled public grades 5 8 pop 3 persons	0.001	0.001	0.795	0.427	-0.001	0.002
census demographics 2017 education enrolled public grades 9 12 pop 3 persons	0.000	0.001	0.226	0.821	-0.001	0.002
census demographics 2017 education enrolled private grades 9 12 pop 3 persons	0.002	0.002	0.843	0.399	-0.002	0.005
census demographics 2017 education enrolled public undergraduate college pop 3 persons	0.000	0.001	0.290	0.772	-0.001	0.001
census demographics 2017 education enrolled public graduate or professional school pop 3 persons	0.002	0.002	1.583	0.113	-0.001	0.006
census demographics 2017 education not enrolled in school pop 3 persons	0.000	0.000	-0.068	0.946	0.000	0.000

census demographics 2017 education attainment high school pop 25 persons 2	-0.001	0.001	-1.862	0.063	-0.002	0.000
census demographics 2017 education attainment some college pop 25 persons	0.000	0.001	-0.691	0.490	-0.002	0.001
census demographics 2017 population in poverty total persons	-0.001	0.001	-1.067	0.286	-0.002	0.001
census demographics 2017 education attainment associate s degree pop 25 persons	0.000	0.001	-0.026	0.979	-0.002	0.002
health children 2017 last health care professional visit 6 months or less	-0.204	0.054	-3.792	0.000	-0.310	-0.099
census demographics 2017 education attainment doctorate degree pop 25 persons	0.793	1.019	0.778	0.436	-1.205	2.791
census demographics 2017 households with income 15000 to 24999 households	0.005	0.009	0.577	0.564	-0.012	0.022
census demographics 2017 households with income 25000 to 34999 households	-0.014	0.010	-1.419	0.156	-0.034	0.005
census demographics 2017 households with income 50000 to 74999 households	0.017	0.012	1.452	0.147	-0.006	0.040
census demographics 2017 households with income 75000 to 99999 households	-0.010	0.008	-1.209	0.227	-0.026	0.006
census demographics 2017 households with income 100000 to 124999 households	0.010	0.014	0.707	0.480	-0.017	0.037
census demographics 2017 households with income 125000 to 149999 households	0.008	0.014	0.580	0.562	-0.019	0.035
health children 2017 fair of poor health status respondent assessed	-0.171	0.066	-2.593	0.010	-0.300	-0.042
census demographics 2017 family head of household male households	-0.001	0.001	-1.538	0.124	-0.003	0.000
census demographics 2017 population non institutional group quarters persons census demographics 2017 household average size persons	-0.046	0.018	-2.600	0.009	-0.080	-0.011
census demographics 2017 other families male householder no wife present with children under 18 other families	-0.001	0.001	-1.953	0.051	-0.003	0.000
census demographics 2017 other families male householder no wife present with no children under 18 other families	0.001	0.001	1.012	0.312	-0.001	0.003
census demographics 2017 other families female householder no husband present with no children under 18 other families	0.001	0.001	0.742	0.458	-0.001	0.002
expenditures home 2017 sewing machines	-0.040	0.012	-3.221	0.001	-0.065	-0.016
expenditures home 2017 girls underwear and sleepwear	-0.035	0.015	-2.374	0.018	-0.065	-0.006
census demographics 2017 non family households female householder with people under 18 households	0.008	0.013	0.570	0.569	-0.018	0.034
census demographics 2017 population urban persons	0.000	0.000	0.045	0.964	0.000	0.000
census demographics 2017 population male persons	0.002	0.003	0.816	0.415	-0.003	0.007
census demographics 2017 black population alone persons	-0.004	0.002	-1.679	0.093	-0.008	0.001
census housing units 2017 housing structure boat rv van other	-0.026	0.009	-2.930	0.003	-0.043	-0.009
census demographics 2017 other race population alone persons	-0.308	0.358	-0.859	0.390	-1.011	0.395

census demographics 2017 two or more races head of households households	0.007	0.006	1.140	0.254	-0.005	0.018
census demographics 2017 families 6 person families	-0.021	0.012	-1.748	0.081	-0.044	0.003
census demographics 2017 families 7 or more person families	0.002	0.003	0.694	0.488	-0.004	0.007
census demographics 2017 non families 1 person households	0.001	0.002	0.419	0.675	-0.003	0.004
census demographics 2017 non families 2 person households	0.001	0.002	0.401	0.689	-0.003	0.005
expenditures food 2017 frozen fruits and fruit juices	-0.025	0.011	-2.303	0.021	-0.047	-0.004
census demographics 2017 non families 5 person households	-0.014	0.010	-1.361	0.174	-0.034	0.006
census demographics 2017 non families 7 or more person households	0.008	0.018	0.480	0.631	-0.026	0.043
census demographics 2017 families aged under 25 years families	-0.003	0.004	-0.726	0.468	-0.010	0.005
census demographics 2017 families aged 25 to 34 years families	0.001	0.003	0.264	0.792	-0.005	0.006
census demographics 2017 families aged 35 to 44 years families	0.004	0.003	1.354	0.176	-0.002	0.010
census demographics 2017 families aged 55 to 64 years families	-0.004	0.004	-1.199	0.231	-0.011	0.003
census demographics 2017 families aged 65 to 74 years families	-0.002	0.003	-0.714	0.475	-0.007	0.003
census demographics 2017 non families aged 25 to 34 years households	-0.001	0.002	-0.818	0.413	-0.004	0.002
expenditures food 2017 school books supplies equipment for elementary high school	-0.015	0.007	-2.155	0.031	-0.029	-0.001
census demographics 2017 non families aged 45 to 54 years households	-0.001	0.001	-0.657	0.511	-0.004	0.002
census demographics 2017 non family households male householder with people under 18 households	-0.015	0.006	-2.583	0.010	-0.026	-0.004
census demographics 2017 population males never married pop 15 persons	0.000	0.000	0.850	0.395	0.000	0.000
census demographics 2017 householder median age years	-0.002	0.002	-1.161	0.246	-0.006	0.002
census demographics 2017 families median age years	0.001	0.002	0.524	0.600	-0.003	0.005
census demographics 2017 family median size number persons	-0.017	0.011	-1.529	0.126	-0.039	0.005
census demographics 2017 households median size number persons	-0.004	0.010	-0.460	0.645	-0.023	0.014
census housing units 2017 housing vacant units sold not occupied	-0.014	0.006	-2.194	0.028	-0.027	-0.002
census housing units 2017 housing vacant units for sale	-0.013	0.004	-3.015	0.003	-0.021	-0.004
census demographics 2017 population density persons per sq mile census demographics 2017 household average size persons	0.000	0.000	1.051	0.293	0.000	0.000
census demographics 2017 education attainment doctorate degree pop 25 persons census demographics 2017 household average size persons	0.049	0.054	0.895	0.371	-0.058	0.155
census demographics 2017 family head of household female households census demographics 2017 household average size persons	0.000	0.000	-1.603	0.109	0.000	0.000

census demographics 2017 other families male householder no wife present with children under 18 other families census demographics 2017 household average size persons	0.000	0.000	0.894	0.372	0.000	0.000
census housing units 2017 housing owner households valued 25000 29999	-0.011	0.003	-3.435	0.001	-0.017	-0.005
census demographics 2017 families aged under 25 years families census demographics 2017 household average size persons	0.000	0.000	-0.541	0.588	0.000	0.000
health adults 2017 type hospital outpatient department	0.193	0.269	0.719	0.472	-0.333	0.719
health adults 2017 hiv ever tested	0.022	0.035	0.621	0.535	-0.047	0.090
health adults 2017 liver disease	-0.020	0.263	-0.077	0.939	-0.535	0.495
census demographics 2017 asian population alone persons	-0.010	0.003	-3.228	0.001	-0.016	-0.004
health adults 2017 difficult of cannot be done climb up 10 steps without resting	0.092	0.220	0.419	0.675	-0.338	0.522
health adults 2017 difficult of cannot be done lift or carry 10 pounds	-0.439	0.275	-1.598	0.110	-0.978	0.099
health adults 2017 body mass index underweight	-0.305	0.259	-1.176	0.240	-0.813	0.203
health adults 2017 body mass index obese	-0.018	0.034	-0.510	0.610	-0.085	0.050
health adults 2017 type some other place	0.074	0.292	0.253	0.800	-0.499	0.646
health children 2017 number school days missed in past 12 months due to illness or injury aged 5 17 6 10 days	-0.023	0.049	-0.470	0.638	-0.120	0.074
health children 2017 number school days missed in past 12 months due to illness or injury aged 5 17 did not go to school	-0.002	0.006	-0.400	0.689	-0.013	0.009
health children 2017 hay fever	0.106	0.065	1.624	0.104	-0.022	0.233
expenditures food 2017 steak	-0.009	0.003	-3.185	0.001	-0.014	-0.003
health children 2017 children 3 to 17 learning disability	0.010	0.054	0.193	0.847	-0.095	0.115
health children 2017 children 3 to 17 attention deficit hyperactivity disorder	-0.066	0.047	-1.391	0.164	-0.159	0.027
census housing units 2017 housing owner households valued 20000 24999	-0.007	0.003	-2.367	0.018	-0.013	-0.001
health children 2017 with a usual place of health care emergency room	0.043	0.068	0.633	0.527	-0.090	0.176
health children 2017 with a usual place of health care hospital outpatient	0.001	0.068	0.010	0.992	-0.132	0.133
health children 2017 with a usual place of health care some other place	-0.072	0.067	-1.075	0.282	-0.203	0.059
census housing units 2017 housing owner households valued 750000 999999	-0.006	0.002	-2.895	0.004	-0.011	-0.002
health children 2017 last health care professional visit more than 1 year but not more than 2 years ago	0.103	0.067	1.547	0.122	-0.028	0.233
census employment 2017 employment other services	-0.006	0.003	-2.304	0.021	-0.012	-0.001
health children 2017 last health care professional visit more than 5 years	-0.022	0.069	-0.316	0.752	-0.157	0.114
health children 2017 delayed care due to cost	-0.073	0.064	-1.140	0.254	-0.198	0.052

health children 2017 children 2 17 years more than 2 years but not more than 5 years ago since last dental visit	0.000	0.062	-0.002	0.998	-0.122	0.122
census housing units 2017 housing no cash rent	-0.006	0.002	-3.621	0.000	-0.010	-0.003
health adults 2017 emphysema health children 2017 uninsured for health care	0.000	0.000	-0.242	0.809	0.000	0.000
census employment 2017 employment car truck van to work carpool empl	0.000	0.001	-0.127	0.899	-0.003	0.002
census employment 2017 employment public transportation to work empl	0.000	0.002	0.000	1.000	-0.003	0.003
census employment 2017 employment streetcar or trolley car to work empl	0.028	0.035	0.794	0.427	-0.041	0.097
census employment 2017 employment railroad to work empl	0.004	0.004	1.010	0.312	-0.004	0.011
census employment 2017 employment taxi to work empl	0.014	0.013	1.033	0.302	-0.012	0.039
census employment 2017 employment bicycle to work empl	-0.005	0.005	-1.043	0.297	-0.014	0.004
census employment 2017 employment other transportation to work empl	0.000	0.004	0.081	0.936	-0.007	0.008
expenditures miscellaneous 2017 gas tank repair replacement expenditures miscellaneous 2017 coin operated apparel laundry and dry cleaning	-0.006	0.003	-2.016	0.044	-0.012	0.000
census demographics 2017 non families aged 55 to 64 years households	-0.005	0.002	-2.838	0.005	-0.008	-0.002
census employment 2017 employment potential	-0.004	0.011	-0.328	0.743	-0.025	0.017
census employment 2017 employment armed forces male	0.002	0.002	1.106	0.269	-0.002	0.007
census employment 2017 employment unemployed female	0.008	0.006	1.247	0.212	-0.005	0.021
census employment 2017 employment construction	-0.005	0.002	-2.345	0.019	-0.008	-0.001
census employment 2017 employment mining quarrying and oil and gas extraction	0.000	0.002	-0.176	0.861	-0.003	0.003
census demographics 2017 families married families	-0.004	0.001	-3.587	0.000	-0.006	-0.002
census housing units 2017 housing owner households valued 250000 299999	-0.003	0.001	-2.549	0.011	-0.005	-0.001
census employment 2017 employment wholesale trade	0.004	0.003	1.223	0.221	-0.002	0.011
census employment 2017 employment information	-0.001	0.004	-0.225	0.822	-0.009	0.007
census employment 2017 employment finance and insurance	-0.004	0.002	-1.560	0.119	-0.009	0.001
census employment 2017 employment management of companies and enterprises	0.048	0.029	1.650	0.099	-0.009	0.105
census employment 2017 employment administrative and support and waste mgt services	-0.003	0.003	-1.002	0.317	-0.009	0.003
census employment 2017 employment manufacturing	-0.002	0.001	-2.037	0.042	-0.004	0.000
census employment 2017 employment arts entertainment and recreation	0.001	0.003	0.309	0.758	-0.004	0.006
census demographics 2017 hispanic households persons	-0.002	0.001	-2.224	0.026	-0.004	0.000
census employment 2017 employment public administration	-0.002	0.002	-1.255	0.210	-0.006	0.001
census employment 2017 employment travel time 30 59 min empl	-0.002	0.001	-2.498	0.013	-0.003	0.000
census employment 2017 occupation service	0.000	0.001	-0.280	0.780	-0.003	0.002

census employment 2017 occupation farming fishing and forestry	-0.002	0.003	-0.967	0.334	-0.008	0.003
census employment 2017 employment private for profit wage and salary workers employee	0.001	0.001	0.670	0.503	-0.001	0.003
expenditures food 2017 other school expenses including rentals expenditures miscellaneous 2017 coin operated apparel laundry and dry cleaning	-0.001	0.000	-2.798	0.005	-0.002	0.000
expenditures home 2017 residential phone service voip and phone cards	-0.001	0.000	-2.019	0.044	-0.002	0.000
census employment 2017 employment local government workers	0.000	0.002	-0.034	0.973	-0.003	0.003
census employment 2017 employment state government workers	0.000	0.002	-0.144	0.886	-0.003	0.003
census employment 2017 employment unpaid family workers	0.013	0.010	1.302	0.193	-0.007	0.034
census housing units 2017 home heating fuel bottled tank or lp gas	-0.001	0.000	-1.893	0.059	-0.002	0.000
census housing units 2017 home heating fuel electricity	0.000	0.000	1.578	0.115	0.000	0.001
census housing units 2017 housing built 1939 or earlier	-0.001	0.000	-2.183	0.029	-0.002	0.000
census housing units 2017 home heating fuel wood	0.001	0.001	1.682	0.093	0.000	0.002
census housing units 2017 home heating fuel solar energy	0.001	0.018	0.072	0.943	-0.033	0.036
census housing units 2017 home heating fuel other fuel	0.003	0.003	1.091	0.275	-0.002	0.008
census housing units 2017 home heating fuel no fuel used	0.000	0.001	-0.056	0.955	-0.002	0.002
census housing units 2017 housing vacant units for rent	0.002	0.002	1.217	0.224	-0.001	0.006
census housing units 2017 housing vacant units rented not occupied	-0.005	0.018	-0.302	0.763	-0.041	0.030
census housing units 2017 home heating fuel fuel oil kerosene etc	-0.001	0.000	-2.737	0.006	-0.001	0.000
expenditures home 2017 personal services	0.000	0.000	-2.283	0.023	-0.001	0.000
census housing units 2017 housing vacant units for migrant workers	-0.018	0.015	-1.218	0.223	-0.047	0.011
census housing units 2017 housing vacant units vacant other	0.000	0.001	-0.176	0.860	-0.003	0.002
census housing units 2017 housing structure with 2 units	0.000	0.001	0.393	0.694	-0.002	0.002
census housing units 2017 housing structure with 3 4 units	0.000	0.001	-0.176	0.861	-0.002	0.002
census housing units 2017 housing structure with 20 49 units	-0.002	0.001	-1.408	0.159	-0.005	0.001
census housing units 2017 housing structure with 50 units	0.001	0.001	0.517	0.605	-0.002	0.003
census employment 2017 occupation management business and financial operations census housing units 2017 housing median year built count year	0.000	0.000	-3.162	0.002	0.000	0.000
census demographics 2017 population urban persons census demographics 2017 household average size persons	0.000	0.000	-2.702	0.007	0.000	0.000
census employment 2017 employment travel time 15 29 min empl census housing units 2017 housing median year built count year	0.000	0.000	-2.483	0.013	0.000	0.000
census housing units 2017 housing owner households valued less than 10000	0.003	0.002	1.446	0.148	-0.001	0.008

census housing units 2017 housing owner households valued 10000 14999	0.000	0.003	0.152	0.879	-0.005	0.006
census demographics 2017 household income median census demographics 2017 household average size persons	0.000	0.000	3.487	0.000	0.000	0.000
census housing units 2017 home heating fuel fuel oil kerosene etc census housing units 2017 housing median year built count year	0.000	0.000	2.322	0.020	0.000	0.000
census housing units 2017 housing owner households valued 35000 39999	0.003	0.003	1.035	0.301	-0.003	0.010
census housing units 2017 housing owner households valued 50000 59999	0.002	0.002	1.217	0.224	-0.001	0.006
census employment 2017 employment health care and social assistance census housing units 2017 housing median year built count year	0.000	0.000	2.310	0.021	0.000	0.000
census employment 2017 employment self employed workers in own not incorporated business census housing units 2017 housing median year built count year	0.000	0.000	3.311	0.001	0.000	0.000
census housing units 2017 housing built 1980 to 1989	0.001	0.001	0.964	0.335	-0.001	0.002
census housing units 2017 housing built 1950 to 1959	0.000	0.001	-0.736	0.462	-0.002	0.001
expenditures food 2017 fresh fish and shellfish expenditures miscellaneous 2017 coin operated apparel laundry and dry cleaning	0.001	0.000	2.595	0.010	0.000	0.001
expenditures food 2017 food on out of town trips	0.001	0.000	2.501	0.012	0.000	0.002
census housing units 2017 housing year moved in 2000 to 2009	0.000	0.001	0.174	0.862	-0.001	0.002
census housing units 2017 housing year moved in 1970 to 1979	0.003	0.002	1.801	0.072	0.000	0.006
census housing units 2017 housing year moved in 1969 or earlier	0.002	0.002	0.858	0.391	-0.003	0.007
census employment 2017 employment walked to work empl census housing units 2017 housing median year built count year	0.000	0.000	0.768	0.442	0.000	0.000
census demographics 2017 non family households male householder with no people under 18 households	0.002	0.001	2.970	0.003	0.001	0.003
census employment 2017 employment agriculture forestry fishing and hunting census housing units 2017 housing median year built count year	0.000	0.000	1.909	0.056	0.000	0.000
census demographics 2017 education enrolled public grades 1 4 pop 3 persons	0.002	0.001	2.043	0.041	0.000	0.003
expenditures food 2017 dinner at fast food take out delivery concession stands buffet and cafeteria	0.002	0.001	2.214	0.027	0.000	0.003
expenditures home 2017 major appliances	0.002	0.001	3.002	0.003	0.001	0.003
census employment 2017 employment travel time 15 29 min empl	0.002	0.001	3.137	0.002	0.001	0.004
expenditures food 2017 nonalcoholic beer	0.268	0.300	0.892	0.372	-0.321	0.857
census housing units 2017 housing rent 750 999	0.003	0.001	2.563	0.010	0.001	0.005
expenditures food 2017 dinner at vending machines and mobile vendors	-0.084	0.060	-1.412	0.158	-0.201	0.033
expenditures food 2017 snacks and nonalcoholic beverages at full service restaurants	0.003	0.004	0.749	0.454	-0.005	0.010
expenditures food 2017 food or board at school	0.000	0.004	-0.002	0.999	-0.009	0.009

expenditures food 2017 catered affairs	-0.003	0.002	-1.370	0.171	-0.007	0.001
expenditures food 2017 elementary and high school tuition	0.000	0.001	0.467	0.641	-0.001	0.002
expenditures food 2017 vocational and technical school tuition	-0.037	0.028	-1.329	0.184	-0.092	0.018
expenditures food 2017 test preparation tutoring services	0.000	0.003	0.003	0.998	-0.005	0.005
census housing units 2017 housing built 1940 to 1949	0.003	0.001	2.773	0.006	0.001	0.004
census employment 2017 employment not in the labor force female	0.003	0.001	2.471	0.014	0.001	0.006
expenditures food 2017 school books supplies equipment for vocational and technical schools	0.125	0.119	1.054	0.292	-0.108	0.358
expenditures food 2017 eggs	-0.004	0.005	-0.835	0.404	-0.014	0.006
census demographics 2017 non families aged 35 to 44 years households	0.003	0.002	2.128	0.033	0.000	0.007
expenditures food 2017 oranges	-0.008	0.007	-1.130	0.259	-0.022	0.006
census employment 2017 employment private not for profit wage and salary workers	0.004	0.002	1.991	0.047	0.000	0.007
expenditures food 2017 school lunches	0.000	0.001	0.598	0.550	-0.001	0.002
expenditures food 2017 whiskey	-0.008	0.006	-1.352	0.176	-0.020	0.004
expenditures food 2017 other alcoholic beverages away	-0.002	0.001	-1.385	0.166	-0.004	0.001
expenditures food 2017 other beef	-0.013	0.007	-1.861	0.063	-0.026	0.001
expenditures food 2017 frankfurters	-0.011	0.014	-0.815	0.415	-0.038	0.016
expenditures food 2017 canned fish and seafood	-0.021	0.022	-0.941	0.347	-0.064	0.023
census employment 2017 occupation sales and office	0.004	0.001	2.781	0.005	0.001	0.007
expenditures food 2017 canned fruits	0.003	0.008	0.340	0.734	-0.012	0.017
expenditures food 2017 canned and bottled fruit juice	0.001	0.002	0.744	0.457	-0.002	0.005
expenditures food 2017 rice	0.001	0.001	0.758	0.449	-0.002	0.003
expenditures food 2017 cookies and crackers	-0.001	0.001	-0.500	0.617	-0.003	0.002
expenditures food 2017 ice cream and related products	0.004	0.002	2.231	0.026	0.001	0.008
census employment 2017 employment private for profit wage and salary workers self	0.008	0.003	3.091	0.002	0.003	0.014
expenditures food 2017 sugar	0.006	0.006	0.903	0.366	-0.007	0.019
expenditures food 2017 margarine	-0.002	0.029	-0.053	0.958	-0.059	0.055
expenditures food 2017 prepared salads	0.007	0.005	1.309	0.191	-0.003	0.017
expenditures food 2017 baby food	-0.003	0.002	-1.417	0.156	-0.006	0.001
expenditures home 2017 window coverings	0.012	0.014	0.906	0.365	-0.014	0.039
expenditures home 2017 infants equipment	-0.002	0.004	-0.629	0.529	-0.009	0.005
expenditures home 2017 clocks and decorative items	-0.002	0.003	-0.732	0.464	-0.008	0.004
expenditures home 2017 lawn and garden equipment	0.000	0.002	0.166	0.868	-0.004	0.004
expenditures home 2017 power tools	0.012	0.007	1.680	0.093	-0.002	0.027
expenditures home 2017 computer accessories	-0.004	0.006	-0.672	0.501	-0.016	0.008
expenditures home 2017 business equipment for home use	-0.009	0.068	-0.132	0.895	-0.142	0.124

expenditures home 2017 mens suits	-0.001	0.003	-0.257	0.797	-0.007	0.005
expenditures home 2017 mens hosiery	0.013	0.016	0.860	0.390	-0.017	0.044
expenditures home 2017 mens nightwear	-0.106	0.137	-0.776	0.438	-0.375	0.162
expenditures home 2017 mens pants and shorts	0.002	0.003	0.917	0.359	-0.003	0.007
expenditures home 2017 mens uniforms	-0.022	0.078	-0.278	0.781	-0.174	0.131
expenditures home 2017 boys nightwear	-0.178	0.153	-1.167	0.243	-0.477	0.121
expenditures home 2017 ground rent	0.000	0.001	0.061	0.951	-0.002	0.002
expenditures home 2017 rent as pay	-0.002	0.001	-1.733	0.083	-0.004	0.000
expenditures home 2017 tenants insurance	0.012	0.006	1.937	0.053	0.000	0.024
census employment 2017 employment health care and social assistance	0.009	0.002	5.639	0.000	0.006	0.013
expenditures home 2017 computer installation	0.035	0.220	0.159	0.874	-0.397	0.467
expenditures home 2017 sofas	-0.002	0.001	-1.817	0.069	-0.003	0.000
expenditures home 2017 outdoor furniture	0.001	0.004	0.356	0.722	-0.007	0.009
census demographics 2017 education attainment professional degree pop 25 persons	0.010	0.003	2.958	0.003	0.003	0.016
expenditures food 2017 other school expenses including rentals	0.027	0.009	3.215	0.001	0.011	0.044
expenditures home 2017 sound equipment accessories	0.019	0.042	0.446	0.656	-0.064	0.101
expenditures home 2017 online gaming services	0.061	0.078	0.779	0.436	-0.092	0.214
expenditures home 2017 vcr s and video disc players	-0.123	0.091	-1.353	0.176	-0.302	0.055
expenditures home 2017 streaming downloading video	0.001	0.028	0.049	0.961	-0.053	0.056
expenditures home 2017 repair of tv radio and sound equipment	-0.187	0.153	-1.222	0.222	-0.488	0.113
expenditures home 2017 womens hosiery	0.028	0.011	2.511	0.012	0.006	0.050
expenditures home 2017 personal digital audio players	-0.122	0.105	-1.158	0.247	-0.329	0.085
expenditures home 2017 musical instruments and accessories	0.009	0.012	0.753	0.452	-0.015	0.034
expenditures home 2017 rental of party supplies for catered affairs	-0.008	0.006	-1.242	0.215	-0.019	0.004
expenditures home 2017 boys shirts and sweaters	0.011	0.007	1.548	0.122	-0.003	0.026
expenditures home 2017 kitchen dining room other linens	-0.038	0.026	-1.482	0.138	-0.089	0.012
expenditures home 2017 stereos radios speakers and sound components including those in vehicles	-0.004	0.003	-1.127	0.260	-0.010	0.003
expenditures miscellaneous 2017 purchase of boat with motor	0.051	0.018	2.908	0.004	0.017	0.085
expenditures home 2017 clothes washer or dryer owner	0.007	0.005	1.523	0.128	-0.002	0.016
expenditures home 2017 womens sweaters shirts tops vests	0.001	0.002	0.830	0.407	-0.002	0.005
expenditures home 2017 interest paid home equity loan	-0.005	0.004	-1.156	0.248	-0.013	0.003
expenditures food 2017 pies tarts turnovers	0.055	0.015	3.561	0.000	0.025	0.085
expenditures home 2017 womens accessories	-0.001	0.001	-0.908	0.364	-0.002	0.001
census demographics 2017 households 6 person households	0.097	0.045	2.143	0.032	0.008	0.185
expenditures home 2017 children under 2	0.003	0.002	1.530	0.126	-0.001	0.007

expenditures miscellaneous 2017 finance late interest charges for credit cards	-0.005	0.007	-0.819	0.413	-0.018	0.007
expenditures miscellaneous 2017 value of savings checking money market and c ds	0.000	0.000	-1.485	0.138	-0.001	0.000
expenditures miscellaneous 2017 care for elderly invalids handicapped etc	-0.034	0.028	-1.212	0.226	-0.089	0.021
expenditures miscellaneous 2017 shoe repair and other shoe service	0.026	0.223	0.116	0.908	-0.412	0.463
expenditures miscellaneous 2017 new cars	0.000	0.003	-0.007	0.995	-0.006	0.006
expenditures miscellaneous 2017 new motorcycles	0.003	0.021	0.120	0.905	-0.039	0.045
expenditures miscellaneous 2017 used motorcycles	-0.017	0.019	-0.917	0.359	-0.054	0.020
expenditures miscellaneous 2017 vehicle products and cleaning services	-0.153	0.083	-1.843	0.065	-0.315	0.010
expenditures miscellaneous 2017 body work and painting	-0.004	0.111	-0.034	0.973	-0.222	0.214
expenditures miscellaneous 2017 towing charges	0.065	0.156	0.415	0.678	-0.241	0.371
expenditures miscellaneous 2017 care in convalescent or nursing home	0.029	0.025	1.162	0.245	-0.020	0.078
expenditures miscellaneous 2017 rental of supportive convalescent medical equipment	-0.247	0.272	-0.909	0.363	-0.780	0.286
health children 2017 children 2 17 years more than 5 years since last dental visit	0.103	0.027	3.819	0.000	0.050	0.156
expenditures miscellaneous 2017 hunting and fishing equipment	-0.006	0.018	-0.305	0.761	-0.041	0.030
expenditures miscellaneous 2017 other photographic supplies	0.180	0.263	0.687	0.492	-0.334	0.695
expenditures miscellaneous 2017 pinball electronic video games	-0.135	0.163	-0.825	0.410	-0.455	0.186
expenditures miscellaneous 2017 lotteries and pari mutuel losses	-0.026	0.024	-1.098	0.272	-0.073	0.021
expenditures miscellaneous 2017 dating services	-0.500	0.265	-1.891	0.059	-1.019	0.018
expenditures home 2017 rental of televisions	0.187	0.067	2.779	0.005	0.055	0.319
expenditures miscellaneous 2017 gift to non cu members of stocks bonds and mutual funds	0.009	0.006	1.526	0.127	-0.003	0.021
expenditures food 2017 dinner at employer and school cafeterias expenditures miscellaneous 2017 coin operated apparel laundry and dry cleaning	0.000	0.001	0.222	0.825	-0.001	0.002
expenditures food 2017 breakfast and brunch at full service restaurants expenditures miscellaneous 2017 coin operated apparel laundry and dry cleaning	0.000	0.000	0.887	0.375	0.000	0.000
expenditures miscellaneous 2017 shopping club membership fees	0.272	0.104	2.613	0.009	0.068	0.476
expenditures food 2017 meals as pay expenditures miscellaneous 2017 coin operated apparel laundry and dry cleaning	0.000	0.000	-0.766	0.444	0.000	0.000
health adults 2017 stroke	0.635	0.265	2.401	0.016	0.117	1.154
expenditures food 2017 fats and oils expenditures miscellaneous 2017 coin operated apparel laundry and dry cleaning	0.000	0.000	-1.060	0.289	-0.001	0.000
expenditures home 2017 office furniture for home use expenditures miscellaneous 2017 coin operated apparel laundry and dry cleaning	0.000	0.001	0.586	0.558	-0.001	0.002

expenditures home 2017 rent expenditures miscellaneous 2017 coin operated apparel laundry and dry cleaning	0.000	0.000	-0.075	0.941	0.000	0.000
expenditures home 2017 housekeeping services expenditures miscellaneous 2017 coin operated apparel laundry and dry cleaning	0.000	0.000	-0.489	0.625	0.000	0.000
expenditures home 2017 rental of televisions expenditures miscellaneous 2017 coin operated apparel laundry and dry cleaning	-0.005	0.003	-1.658	0.097	-0.012	0.001
expenditures home 2017 boys uniforms and active sportswear expenditures miscellaneous 2017 coin operated apparel laundry and dry cleaning	-0.001	0.001	-1.676	0.094	-0.002	0.000
expenditures miscellaneous 2017 newspapers expenditures miscellaneous 2017 coin operated apparel laundry and dry cleaning	3.529	1.111	3.177	0.002	1.352	5.706
(Intercept)	18.394	4.172	4.410	0.000	10.218	26.571
expenditures miscellaneous 2017 global positioning system devices expenditures miscellaneous 2017 coin operated apparel laundry and dry cleaning	-0.002	0.002	-1.086	0.277	-0.007	0.002
expenditures miscellaneous 2017 vacation clubs expenditures miscellaneous 2017 coin operated apparel laundry and dry cleaning	0.000	0.000	-0.423	0.672	0.000	0.000

HH=Household
Fam=Family
Pop=Population
Non Fam=Non family
OT=Other
ER=Emergency room
RV=recreational vehicle
Equip=equipment
Misc.=miscellaneous
BCBS=Blue Cross Blue Shield
OOT=Out of town
RIHC=resource intensive healthcare
