# Supplementary material for: Can diverse population characteristics be leveraged in a machine learning pipeline to predict resource intensive healthcare utilization among hospital service areas?
Source: BMC Health Serv Res. 2022 Jun 30;22:847. doi: 10.1186/s12913-022-08154-4 (PMC9248096; doi:10.1186/s12913-022-08154-4)
Supplement: Supplementary file 17 — Additional file 17. [file 12913_2022_8154_MOESM17_ESM.pdf]

Additional File 17. Multiple Linear Regression of Log Hospital Expenditures Per Capita in Hospital Service Areas 2017 (N=3,174, Adjusted R^2 0.829)

- Additional File 17
  - File format: PDF
  - File title: Multiple Linear Regression of Log Hospital Expenditures Per Capita in Hospital Service Areas 2017 (N=3,174, Adjusted R^2 0.829)
  - File description: Long table, model output for regression model for hospital expenditures per capita

Variable (expressed per capita or per capita percent)	Coefficient	Standard Error	Z Statistic	P Value	95% Lower	95% Upper
(Intercept)	4.891	6.595	0.742	0.458	-8.035	17.818
census demographics 2017 household income average	0.000	0.000	-1.474	0.141	0.000	0.000
census demographics 2017 other race population alone persons	-0.939	0.233	-4.038	0.000	-1.395	-0.483
census demographics 2017 population speaks other language pop 5 persons	0.021	0.061	0.347	0.729	-0.099	0.141
census demographics 2017 population citizenship foreign born not a citizen persons	-0.001	0.001	-1.566	0.117	-0.002	0.000
census demographics 2017 veterans total persons	0.000	0.001	-0.012	0.991	-0.001	0.001
census demographics 2017 population institutional group quarters persons	0.000	0.000	-1.040	0.299	0.000	0.000
census demographics 2017 householder aged 25 to 34 years households	-0.013	0.007	-1.878	0.060	-0.027	0.001
expenditures miscellaneous 2017 termination fee for car truck lease	-0.706	0.191	-3.691	0.000	-1.081	-0.331
expenditures home 2017 mens nightwear	-0.611	0.101	-6.021	0.000	-0.810	-0.412
census demographics 2017 householder aged 85 years and over households	0.002	0.005	0.515	0.606	-0.007	0.012
expenditures miscellaneous 2017 rental of supportive convalescent medical equipment	-0.518	0.188	-2.751	0.006	-0.888	-0.149
expenditures miscellaneous 2017 rental of medical equipment	-0.408	0.207	-1.976	0.048	-0.813	-0.003
expenditures miscellaneous 2017 rental and repair of miscellaneous sports equipment	-0.407	0.161	-2.520	0.012	-0.723	-0.090
expenditures miscellaneous 2017 shoe repair and other shoe service	-0.386	0.159	-2.421	0.016	-0.699	-0.073
census demographics 2017 education enrolled public or private school pop 3 persons	0.000	0.000	-0.401	0.688	-0.001	0.001
expenditures home 2017 infant coat jacket snowsuit	-0.329	0.157	-2.096	0.036	-0.636	-0.021
census demographics 2017 education enrolled public kindergarten pop 3 persons	0.001	0.001	0.593	0.553	-0.001	0.002
census demographics 2017 education enrolled private kindergarten pop 3 persons	-0.001	0.003	-0.245	0.806	-0.007	0.005

census demographics 2017 education enrolled public grades 1 4 pop 3 persons	0.000	0.001	0.385	0.700	-0.001	0.001
census demographics 2017 education enrolled private grades 1 4 pop 3 persons	0.000	0.001	-0.046	0.964	-0.003	0.003
expenditures miscellaneous 2017 coolant brake fluid transmission fluid and other additives	-0.305	0.090	-3.371	0.001	-0.482	-0.127
census demographics 2017 education enrolled private grades 5 8 pop 3 persons	0.000	0.001	-0.026	0.980	-0.003	0.003
census demographics 2017 education enrolled public grades 9 12 pop 3 persons	0.000	0.001	-0.455	0.649	-0.001	0.001
census demographics 2017 education enrolled public undergraduate college pop 3 persons	0.000	0.000	-0.878	0.380	-0.001	0.000
health children 2017 last health care professional visit more than 2 years but less than 5 years ago	-0.236	0.048	-4.896	0.000	-0.330	-0.141
census demographics 2017 education enrolled private graduate or professional school pop 3 persons	0.002	0.001	1.617	0.106	0.000	0.005
expenditures food 2017 school books supplies equipment for day care nursery	-0.157	0.061	-2.593	0.010	-0.276	-0.038
census demographics 2017 education attainment some college pop 25 persons	0.000	0.000	1.101	0.271	0.000	0.001
census demographics 2017 population in poverty total persons	0.000	0.000	-1.051	0.293	-0.001	0.000
census demographics 2017 education attainment associate s degree pop 25 persons	0.000	0.001	-0.515	0.606	-0.001	0.001
census demographics 2017 education attainment bachelor s degree pop 25 persons	0.001	0.001	1.193	0.233	0.000	0.002
health children 2017 children 3 to 17 attention deficit hyperactivity disorder	-0.146	0.037	-3.949	0.000	-0.219	-0.074
census demographics 2017 education attainment doctorate degree pop 25 persons	0.224	0.577	0.389	0.698	-0.907	1.356
census demographics 2017 households with income 15000 to 24999 households	0.014	0.008	1.765	0.078	-0.002	0.029
census demographics 2017 households with income 25000 to 34999 households	-0.005	0.008	-0.621	0.534	-0.020	0.011
census demographics 2017 households with income 50000 to 74999 households	0.001	0.007	0.207	0.836	-0.012	0.014
census demographics 2017 households with income 75000 to 99999 households	0.006	0.008	0.774	0.439	-0.009	0.021
census demographics 2017 households with income 100000 to 124999 households	-0.004	0.005	-0.674	0.500	-0.014	0.007
census demographics 2017 households 1 person households	0.000	0.004	0.087	0.930	-0.007	0.007
census demographics 2017 households 3 person households	-0.003	0.002	-1.502	0.133	-0.008	0.001
health children 2017 last health care professional visit 6 months or less	-0.122	0.043	-2.834	0.005	-0.207	-0.038
census demographics 2017 households 5 person households	0.000	0.004	0.009	0.993	-0.009	0.009
census demographics 2017 head of household male households	0.001	0.002	0.565	0.572	-0.002	0.004
census demographics 2017 families married families	0.000	0.001	0.389	0.697	-0.002	0.003
expenditures home 2017 rental and repair of musical instruments	-0.121	0.060	-2.028	0.043	-0.238	-0.004

census demographics 2017 other families male householder no wife present with children under 18 other families	0.000	0.000	0.698	0.486	-0.001	0.001
census demographics 2017 other families female householder no husband present with no children under 18 other families	0.000	0.000	0.325	0.745	-0.001	0.001
census demographics 2017 non family head of household male households	0.001	0.001	1.015	0.310	0.000	0.002
census demographics 2017 non family households male householder with people under 18 households	-0.002	0.004	-0.581	0.561	-0.009	0.005
census demographics 2017 non family households male householder with no people under 18 households	0.000	0.000	0.983	0.326	0.000	0.001
census demographics 2017 non family households female householder with people under 18 households	0.016	0.008	1.849	0.064	-0.001	0.032
health children 2017 very good health status respondent assessed	-0.117	0.044	-2.657	0.008	-0.204	-0.031
census demographics 2017 population male persons	0.002	0.005	0.434	0.664	-0.007	0.012
health children 2017 skin allergies	-0.110	0.039	-2.795	0.005	-0.187	-0.033
health children 2017 without a usual place of health care	-0.105	0.047	-2.229	0.026	-0.196	-0.013
census demographics 2017 american indian and alaska native head of households households	0.008	0.004	1.884	0.060	0.000	0.015
census demographics 2017 two or more races head of households households	-0.007	0.004	-1.882	0.060	-0.015	0.000
expenditures home 2017 womens uniforms	-0.066	0.031	-2.129	0.033	-0.126	-0.005
census demographics 2017 families 6 person families	-0.002	0.002	-0.895	0.371	-0.006	0.002
census demographics 2017 families 7 or more person families	0.002	0.002	1.179	0.238	-0.001	0.006
census demographics 2017 non families 2 person households	0.001	0.001	0.632	0.528	-0.001	0.002
census demographics 2017 non families 4 person households	-0.005	0.003	-1.587	0.113	-0.011	0.001
census demographics 2017 non families 6 person households	0.001	0.002	0.415	0.679	-0.004	0.006
census demographics 2017 non families 7 or more person households	-0.004	0.011	-0.358	0.721	-0.026	0.018
census demographics 2017 families aged under 25 years families	0.001	0.002	0.348	0.728	-0.003	0.004
census demographics 2017 families aged 55 to 64 years families	0.002	0.002	1.271	0.204	-0.001	0.005
census demographics 2017 families aged 65 to 74 years families	-0.003	0.002	-1.313	0.189	-0.007	0.001
expenditures home 2017 flatware	-0.049	0.015	-3.227	0.001	-0.079	-0.019
census demographics 2017 non families aged 25 to 34 years households	0.000	0.001	-0.533	0.594	-0.002	0.001
census demographics 2017 non families aged 45 to 54 years households	-0.001	0.001	-1.587	0.113	-0.003	0.000
census demographics 2017 non families aged 65 to 74 years households	-0.002	0.001	-1.720	0.086	-0.004	0.000
expenditures home 2017 kitchen dining room other linens	-0.033	0.012	-2.798	0.005	-0.056	-0.010
census demographics 2017 population median age years	0.001	0.001	1.686	0.092	0.000	0.002

census demographics 2017 families median age years	-0.001	0.001	-1.429	0.153	-0.003	0.000
census demographics 2017 households median size number persons	-0.009	0.006	-1.458	0.145	-0.021	0.003
health adults 2017 full guidelines strengthening and aerobic combined met both muscle strengthening and aerobic guidelines	0.000	0.080	0.005	0.996	-0.156	0.157
health adults 2017 serious psychological distress	-0.175	0.206	-0.852	0.394	-0.579	0.228
health adults 2017 type hospital outpatient department	0.331	0.227	1.460	0.144	-0.113	0.776
expenditures home 2017 sewing machines	-0.028	0.008	-3.725	0.000	-0.043	-0.013
health adults 2017 last dental visit more than 1 year but not more than 2 years ago	-0.056	0.184	-0.308	0.758	-0.416	0.303
health adults 2017 last dental visit never	-0.020	0.216	-0.093	0.926	-0.444	0.404
health adults 2017 breast cancer	0.254	0.223	1.142	0.254	-0.182	0.690
health adults 2017 hopelessness all or most of the time	-0.014	0.217	-0.066	0.948	-0.440	0.412
health adults 2017 stroke	-0.014	0.213	-0.067	0.946	-0.433	0.404
health adults 2017 difficult of cannot be done climb up 10 steps without resting	-0.168	0.171	-0.986	0.324	-0.502	0.166
health adults 2017 difficult of cannot be done grasp or handle small objects	-0.058	0.260	-0.223	0.823	-0.568	0.452
health adults 2017 difficult of cannot be done push or pull large objects	0.087	0.180	0.486	0.627	-0.265	0.440
health adults 2017 emphysema	0.247	0.226	1.093	0.275	-0.196	0.690
health adults 2017 ever had asthma	0.116	0.151	0.766	0.444	-0.181	0.412
health adults 2017 every day smokers	-0.280	0.175	-1.598	0.110	-0.624	0.064
health adults 2017 alcohol lifetime abstainer	-0.061	0.049	-1.238	0.216	-0.157	0.035
health adults 2017 alcohol current infrequent	-0.215	0.127	-1.692	0.091	-0.465	0.034
health adults 2017 body mass index healthy weight	0.032	0.022	1.475	0.140	-0.010	0.074
health adults 2017 without a usual place of health care	-0.098	0.089	-1.103	0.270	-0.273	0.076
health adults 2017 last doctor visit more than six months but less than 1 year	0.287	0.162	1.777	0.076	-0.030	0.604
health adults 2017 last doctor visit more than 2 years but less than 5 years ago	-0.138	0.217	-0.636	0.525	-0.564	0.288
health adults 2017 last doctor visit more than 5 years excluding never	-0.383	0.223	-1.717	0.086	-0.820	0.054
health children 2017 number school days missed in past 12 months due to illness or injury aged 5 17 6 10 days	0.000	0.034	-0.010	0.992	-0.067	0.067
health children 2017 number school days missed in past 12 months due to illness or injury aged 5 17 11 or more days	0.016	0.035	0.450	0.653	-0.053	0.085
census employment 2017 employment potential	-0.024	0.008	-3.214	0.001	-0.039	-0.009
health children 2017 number school days missed in past 12 months due to illness or injury aged 5 17 did not go to school	-0.017	0.004	-4.790	0.000	-0.024	-0.010
health children 2017 children receiving special education or early intervention services	0.073	0.047	1.561	0.119	-0.019	0.165

health children 2017 ever told had asthma	0.045	0.046	0.984	0.325	-0.045	0.134
health children 2017 still have asthma	0.026	0.042	0.623	0.533	-0.056	0.109
health children 2017 hay fever	-0.062	0.047	-1.332	0.183	-0.154	0.029
health children 2017 respiratory allergies	0.053	0.045	1.185	0.236	-0.035	0.142
health children 2017 food allergies	-0.071	0.047	-1.527	0.127	-0.163	0.020
expenditures home 2017 rental of furniture	-0.015	0.007	-2.228	0.026	-0.029	-0.002
health children 2017 children 3 to 17 learning disability	-0.055	0.039	-1.421	0.156	-0.132	0.021
census demographics 2017 families 4 person families	-0.013	0.005	-2.930	0.003	-0.022	-0.004
census demographics 2017 householder aged 35 to 44 years households	-0.011	0.005	-2.046	0.041	-0.022	0.000
census demographics 2017 householder aged 55 to 64 years households	-0.011	0.005	-2.329	0.020	-0.019	-0.002
health children 2017 fair of poor health status respondent assessed	-0.041	0.049	-0.830	0.407	-0.138	0.056
expenditures home 2017 stereos radios speakers and sound components including those in vehicles	-0.010	0.004	-2.745	0.006	-0.017	-0.003
health children 2017 with a usual place of health care	0.050	0.044	1.126	0.260	-0.037	0.137
health children 2017 with a usual place of health care doctor s office	0.007	0.011	0.710	0.478	-0.013	0.028
health children 2017 with a usual place of health care emergency room	0.002	0.047	0.043	0.966	-0.090	0.094
expenditures food 2017 oranges	-0.008	0.004	-1.984	0.047	-0.015	0.000
health children 2017 with a usual place of health care some other place	0.087	0.048	1.808	0.071	-0.007	0.181
census housing units 2017 housing owner households valued 25000 29999	-0.006	0.002	-2.763	0.006	-0.010	-0.002
census employment 2017 employment public administration	-0.004	0.001	-3.129	0.002	-0.007	-0.002
census housing units 2017 housing owner households valued 750000 999999	-0.004	0.002	-2.396	0.017	-0.007	-0.001
census demographics 2017 families aged 75 years and over families	-0.003	0.002	-2.079	0.038	-0.007	0.000
health children 2017 last health care professional visit more than 5 years	-0.064	0.047	-1.344	0.179	-0.157	0.029
health children 2017 delayed care due to cost	-0.031	0.045	-0.688	0.492	-0.118	0.057
health children 2017 children 2 17 years yes unmet dental need	-0.062	0.041	-1.517	0.129	-0.142	0.018
health children 2017 children 2 17 years no unmet dental need	0.030	0.031	0.959	0.338	-0.031	0.092
health children 2017 children 2 17 years more than 1 year but not more than 2 years since last dental visit	0.071	0.040	1.756	0.079	-0.008	0.150
health children 2017 children 2 17 years more than 2 years but not more than 5 years ago since last dental visit	0.057	0.042	1.363	0.173	-0.025	0.138
census demographics 2017 families married with no children under 18 families	-0.002	0.001	-2.955	0.003	-0.004	-0.001
census employment 2017 employment bus or trolley bus to work empl	-0.001	0.002	-0.654	0.513	-0.004	0.002
census employment 2017 employment streetcar or trolley car to work empl	0.040	0.022	1.794	0.073	-0.004	0.083

census employment 2017 employment subway or elevated to work empl	-0.002	0.001	-1.741	0.082	-0.005	0.000
census employment 2017 employment ferry to work empl	-0.007	0.013	-0.533	0.594	-0.033	0.019
census employment 2017 employment motorcycle to work empl	-0.004	0.008	-0.502	0.616	-0.020	0.012
census employment 2017 employment bicycle to work empl	-0.002	0.003	-0.774	0.439	-0.008	0.003
census employment 2017 employment walked to work empl	0.001	0.001	0.486	0.627	-0.002	0.003
census employment 2017 employment other transportation to work empl	0.002	0.002	0.671	0.502	-0.003	0.006
census employment 2017 occupation professional and related	-0.002	0.001	-2.121	0.034	-0.004	0.000
census employment 2017 employment travel time 60 89 min empl	0.001	0.001	1.041	0.298	-0.001	0.003
census employment 2017 employment travel time 90 min empl	0.000	0.002	-0.213	0.832	-0.003	0.003
census employment 2017 employment private for profit wage and salary workers employee	-0.002	0.001	-2.882	0.004	-0.004	-0.001
census employment 2017 employment civilian males	-0.001	0.001	-0.827	0.408	-0.003	0.001
census employment 2017 employment armed forces female	0.000	0.009	-0.033	0.974	-0.017	0.017
census housing units 2017 housing median year built count year	-0.002	0.000	-3.827	0.000	-0.003	-0.001
expenditures food 2017 other alcoholic beverages away	-0.002	0.001	-2.093	0.036	-0.003	0.000
census employment 2017 employment mining quarrying and oil and gas extraction	0.002	0.001	1.899	0.058	0.000	0.004
census employment 2017 employment wholesale trade	0.000	0.002	0.124	0.901	-0.004	0.004
census housing units 2017 housing built 2000 to 2009	-0.001	0.000	-2.610	0.009	-0.002	0.000
census employment 2017 employment transportation and warehousing	0.001	0.001	0.603	0.546	-0.002	0.003
census employment 2017 employment utilities	0.002	0.002	0.741	0.459	-0.003	0.006
census employment 2017 employment finance and insurance	0.002	0.001	1.401	0.161	-0.001	0.005
census employment 2017 employment professional scientific and technical services	-0.002	0.002	-1.424	0.154	-0.005	0.001
census employment 2017 employment educational services	0.000	0.001	0.035	0.972	-0.002	0.003
census housing units 2017 housing structure with 1 unit attached	-0.001	0.000	-2.157	0.031	-0.002	0.000
census employment 2017 employment arts entertainment and recreation	0.002	0.002	1.209	0.227	-0.001	0.006
census demographics 2017 population urban persons	0.000	0.000	-5.063	0.000	-0.001	0.000
census employment 2017 employment other services	-0.003	0.002	-1.762	0.078	-0.007	0.000
census demographics 2017 population males never married pop 15 persons	0.000	0.000	-5.204	0.000	0.000	0.000
census demographics 2017 household income median	0.000	0.000	2.775	0.006	0.000	0.000
census employment 2017 occupation service	0.001	0.001	0.978	0.328	-0.001	0.003

census demographics 2017 education not enrolled in school pop 3 persons	0.000	0.000	2.032	0.042	0.000	0.000
census housing units 2017 home heating fuel bottled tank or lp gas	0.001	0.000	3.504	0.000	0.000	0.001
census housing units 2017 home heating fuel wood	0.001	0.000	2.971	0.003	0.000	0.002
expenditures home 2017 babysitting and child care	0.001	0.001	2.101	0.036	0.000	0.002
census employment 2017 employment self employed workers in own not incorporated business	-0.002	0.001	-1.486	0.137	-0.004	0.001
census demographics 2017 education enrolled public grades 5 8 pop 3 persons	0.001	0.001	2.221	0.026	0.000	0.002
census housing units 2017 housing median year moved in count year	0.002	0.000	3.962	0.000	0.001	0.003
census employment 2017 employment not in the labor force female	0.002	0.001	2.095	0.036	0.000	0.003
census housing units 2017 home heating fuel fuel oil kerosene etc	0.000	0.000	-1.431	0.153	-0.001	0.000
census housing units 2017 home heating fuel coal or coke	0.000	0.002	0.065	0.948	-0.003	0.004
expenditures home 2017 men 16 and over	0.002	0.001	2.727	0.006	0.001	0.003
census housing units 2017 home heating fuel other fuel	0.000	0.002	-0.235	0.814	-0.004	0.003
census housing units 2017 home heating fuel no fuel used	0.001	0.001	1.721	0.085	0.000	0.002
census housing units 2017 housing occupied units	0.000	0.001	0.176	0.860	-0.002	0.002
census housing units 2017 housing vacant units rented not occupied	0.001	0.012	0.092	0.926	-0.022	0.024
census housing units 2017 housing vacant units for sale	-0.001	0.003	-0.436	0.663	-0.006	0.004
census demographics 2017 education enrolled public preprimary pop 3 persons	0.002	0.001	2.246	0.025	0.000	0.003
census housing units 2017 housing vacant units vacant other	0.000	0.001	0.072	0.943	-0.001	0.002
census demographics 2017 education enrolled public graduate or professional school pop 3 persons	0.002	0.001	2.098	0.036	0.000	0.004
census housing units 2017 housing structure with 2 units	-0.001	0.001	-1.441	0.150	-0.002	0.000
census housing units 2017 housing structure with 3 4 units	-0.001	0.001	-0.961	0.337	-0.002	0.001
census housing units 2017 housing structure mobile home	-0.001	0.000	-1.554	0.120	-0.001	0.000
census housing units 2017 housing built 1940 to 1949	0.002	0.001	3.525	0.000	0.001	0.003
census housing units 2017 housing rent 500 749	0.000	0.001	0.640	0.522	-0.001	0.001
census employment 2017 employment retail trade	0.002	0.001	2.235	0.025	0.000	0.004
census housing units 2017 housing rent 1250 1499	-0.002	0.002	-0.971	0.332	-0.005	0.002
census housing units 2017 housing rent 1500 1999	0.002	0.002	0.971	0.332	-0.002	0.005
census housing units 2017 housing no cash rent	-0.001	0.001	-1.176	0.240	-0.004	0.001
census housing units 2017 housing owner households valued 10000 14999	0.001	0.002	0.389	0.697	-0.003	0.005
census housing units 2017 housing owner households valued 20000 24999	-0.003	0.002	-1.708	0.088	-0.007	0.000
census housing units 2017 housing rent 750 999	0.002	0.001	3.108	0.002	0.001	0.004

census housing units 2017 housing owner households valued 50000 59999	-0.001	0.001	-1.230	0.219	-0.004	0.001
census housing units 2017 housing owner households valued 80000 89999	0.000	0.001	0.074	0.941	-0.002	0.002
census housing units 2017 housing owner households valued 90000 99999	-0.001	0.001	-1.138	0.255	-0.004	0.001
census housing units 2017 housing owner households valued 100000 124999	-0.001	0.001	-1.046	0.296	-0.002	0.001
census housing units 2017 housing owner households valued 125000 149999	0.000	0.001	-0.052	0.958	-0.002	0.002
census housing units 2017 housing owner households valued 150000 174999	0.000	0.001	-0.393	0.694	-0.002	0.001
census housing units 2017 housing owner households valued 175000 199999	0.001	0.001	0.526	0.599	-0.002	0.003
census housing units 2017 housing owner households valued 400000 499999	0.000	0.001	0.229	0.819	-0.002	0.003
census employment 2017 employment local government workers	0.002	0.001	2.165	0.030	0.000	0.005
census housing units 2017 housing owner households valued more than 1000000	0.002	0.001	2.029	0.043	0.000	0.005
census employment 2017 employment accommodation and food services	0.003	0.001	2.104	0.035	0.000	0.005
census housing units 2017 housing built 1970 to 1979	0.000	0.000	0.179	0.858	-0.001	0.001
census housing units 2017 housing built 1960 to 1969	0.000	0.000	-0.623	0.534	-0.001	0.001
census housing units 2017 housing built 1950 to 1959	0.000	0.000	-0.683	0.495	-0.001	0.000
census employment 2017 employment travel time less than 15 min empl	0.003	0.000	8.630	0.000	0.002	0.003
census housing units 2017 housing year moved in 2010 or later	-0.001	0.001	-1.723	0.085	-0.003	0.000
census housing units 2017 housing year moved in 1990 to 1999	-0.002	0.001	-1.779	0.075	-0.004	0.000
census housing units 2017 housing year moved in 1970 to 1979	0.002	0.001	1.526	0.127	-0.001	0.004
census housing units 2017 housing year moved in 1969 or earlier	-0.001	0.002	-0.893	0.372	-0.004	0.002
census housing units 2017 housing owner households with mortgage any	0.000	0.001	-0.086	0.932	-0.002	0.002
census housing units 2017 housing owner households with no mortgage	0.000	0.001	0.354	0.724	-0.002	0.003
expenditures food 2017 dinner at fast food take out delivery concession stands buffet and cafeteria	0.000	0.000	-0.554	0.580	-0.001	0.001
expenditures food 2017 dinner at vending machines and mobile vendors	-0.020	0.029	-0.681	0.496	-0.077	0.037
expenditures food 2017 breakfast and brunch at vending machines and mobile vendors	0.007	0.007	0.959	0.337	-0.007	0.021
expenditures food 2017 food or board at school	-0.001	0.001	-0.381	0.703	-0.003	0.002
expenditures food 2017 education	0.000	0.000	-1.641	0.101	0.000	0.000
expenditures food 2017 school books supplies equipment for college	0.001	0.001	0.658	0.511	-0.001	0.002
expenditures food 2017 school books supplies equipment for vocational and technical schools	0.039	0.060	0.662	0.508	-0.077	0.156
census employment 2017 employment private not for profit wage and salary workers	0.003	0.001	2.400	0.016	0.001	0.005

expenditures food 2017 ice cream and related products	0.002	0.001	1.489	0.137	-0.001	0.004
census housing units 2017 housing rent less than 250	0.003	0.001	3.220	0.001	0.001	0.005
expenditures food 2017 food on out of town trips	0.000	0.000	1.583	0.113	0.000	0.001
expenditures food 2017 meals as pay	0.000	0.001	-0.414	0.679	-0.002	0.001
expenditures food 2017 whiskey	0.002	0.003	0.680	0.497	-0.004	0.009
census demographics 2017 households with 2 vehicles households	0.003	0.002	2.165	0.031	0.000	0.006
expenditures food 2017 lamb organ meats and others	0.004	0.003	1.653	0.099	-0.001	0.010
expenditures food 2017 other fresh fruits	0.001	0.001	1.014	0.311	-0.001	0.004
expenditures food 2017 pies tarts turnovers	0.007	0.005	1.404	0.160	-0.003	0.016
expenditures food 2017 prepared flour mixes	0.001	0.002	0.322	0.747	-0.004	0.005
expenditures food 2017 artificial sweeteners	-0.039	0.026	-1.511	0.131	-0.089	0.012
census demographics 2017 households with 1 vehicle households	0.003	0.001	2.335	0.020	0.001	0.006
expenditures home 2017 lawn and garden equipment	0.004	0.001	2.636	0.008	0.001	0.006
expenditures home 2017 infants equipment	0.001	0.002	0.326	0.745	-0.004	0.005
census demographics 2017 households with no vehicles households	0.004	0.002	2.086	0.037	0.000	0.008
expenditures home 2017 closet and storage items	0.007	0.008	0.885	0.376	-0.009	0.023
census employment 2017 employment agriculture forestry fishing and hunting	0.004	0.001	5.091	0.000	0.003	0.006
expenditures home 2017 apparel and services	0.000	0.000	1.243	0.214	0.000	0.000
census employment 2017 employment federal government workers	0.005	0.001	3.056	0.002	0.002	0.007
census demographics 2017 households with 3 vehicles households	0.005	0.002	2.607	0.009	0.001	0.009
census employment 2017 employment health care and social assistance	0.007	0.001	6.537	0.000	0.005	0.009
expenditures home 2017 mens active sportswear	0.007	0.009	0.831	0.406	-0.010	0.025
expenditures home 2017 mens costumes	-0.180	0.096	-1.880	0.060	-0.367	0.008
expenditures home 2017 boys suits sportcoats vests	0.016	0.042	0.380	0.704	-0.066	0.097
expenditures home 2017 womens sportcoats tailored jackets	0.005	0.003	1.768	0.077	-0.001	0.011
expenditures home 2017 ground rent	0.000	0.002	0.207	0.836	-0.003	0.003
expenditures home 2017 rent as pay	0.000	0.000	-1.002	0.317	-0.001	0.000
expenditures home 2017 bottled gas	-0.001	0.003	-0.180	0.857	-0.006	0.005
expenditures home 2017 personal services	0.000	0.000	-0.538	0.591	0.000	0.000
expenditures home 2017 water softening service	0.007	0.015	0.465	0.642	-0.023	0.037
expenditures home 2017 household laundry and dry cleaning sent out nonclothing not coin operated	0.000	0.003	0.018	0.986	-0.006	0.006
expenditures home 2017 computer installation	0.004	0.090	0.044	0.965	-0.172	0.180
expenditures home 2017 miscellaneous household products	-0.001	0.002	-0.713	0.476	-0.005	0.002
expenditures home 2017 stationery stationery supplies giftwrap	0.006	0.004	1.576	0.115	-0.001	0.014

expenditures home 2017 living room tables	-0.010	0.007	-1.338	0.181	-0.024	0.004
expenditures home 2017 girls footwear	0.008	0.004	2.133	0.033	0.001	0.015
expenditures home 2017 audio and visual equipment and services	0.003	0.002	1.597	0.110	-0.001	0.006
expenditures home 2017 televisions	0.001	0.005	0.159	0.874	-0.009	0.011
expenditures home 2017 cable and satellite television services	-0.002	0.002	-1.415	0.157	-0.006	0.001
expenditures home 2017 sound equipment accessories	0.021	0.021	1.007	0.314	-0.020	0.063
expenditures home 2017 applications games ringtones for handheld devices	-0.153	0.089	-1.720	0.086	-0.327	0.021
expenditures home 2017 repair of tv radio and sound equipment	-0.236	0.133	-1.778	0.075	-0.496	0.024
expenditures home 2017 musical instruments and accessories	0.005	0.007	0.749	0.454	-0.008	0.019
census demographics 2017 education attainment professional degree pop 25 persons	0.008	0.002	3.818	0.000	0.004	0.012
expenditures home 2017 lamps lighting fixtures ceiling fans	0.009	0.004	2.297	0.022	0.001	0.017
expenditures home 2017 boys uniforms and active sportswear	-0.012	0.006	-1.927	0.054	-0.025	0.000
expenditures food 2017 jams preserves other sweets	0.010	0.004	2.272	0.023	0.001	0.018
expenditures home 2017 flooring installation repair replacement owned	-0.001	0.002	-0.705	0.481	-0.005	0.002
expenditures home 2017 girls shirts blouses sweaters and vests	0.005	0.006	0.840	0.401	-0.007	0.017
expenditures home 2017 infant underwear	0.010	0.005	2.016	0.044	0.000	0.019
census housing units 2017 housing vacant units sold not occupied	0.018	0.004	4.089	0.000	0.009	0.026
expenditures home 2017 interest paid home equity loan	0.001	0.003	0.232	0.816	-0.005	0.006
expenditures home 2017 womens hosiery	-0.004	0.009	-0.451	0.652	-0.021	0.013
census demographics 2017 asian households households	0.018	0.009	1.971	0.049	0.000	0.037
expenditures home 2017 womens costumes	-0.021	0.044	-0.470	0.638	-0.106	0.065
expenditures home 2017 girls costumes	-0.052	0.028	-1.897	0.058	-0.106	0.002
expenditures home 2017 mens hosiery	0.025	0.009	2.674	0.008	0.007	0.043
census demographics 2017 households 4 person households	0.044	0.017	2.627	0.009	0.011	0.077
expenditures miscellaneous 2017 finance late interest charges for other loans	0.062	0.028	2.230	0.026	0.007	0.116
expenditures miscellaneous 2017 auto truck rental	-0.029	0.034	-0.874	0.382	-0.095	0.036
health children 2017 prescription medication taken regularly for at least 3 months	0.097	0.042	2.297	0.022	0.014	0.180
health children 2017 children 2 17 years more than 5 years since last dental visit	0.104	0.026	3.990	0.000	0.053	0.155
expenditures miscellaneous 2017 finance late interest charges for student loans	0.012	0.007	1.670	0.095	-0.002	0.026
expenditures miscellaneous 2017 care for elderly invalids handicapped etc	0.000	0.019	-0.013	0.990	-0.038	0.038
health children 2017 emergency room visits in past 12 months for children under 18 one	0.108	0.039	2.754	0.006	0.031	0.185

health children 2017 last health care professional visit more than six months but less than 1 year	0.109	0.045	2.395	0.017	0.020	0.197
expenditures miscellaneous 2017 towing charges	-0.208	0.116	-1.791	0.073	-0.436	0.020
expenditures miscellaneous 2017 taxi fares and limousine services	0.039	0.028	1.411	0.158	-0.015	0.093
expenditures miscellaneous 2017 adult diapers	-0.233	0.139	-1.672	0.095	-0.506	0.040
health children 2017 with a usual place of health care hospital outpatient	0.122	0.048	2.575	0.010	0.029	0.216
health children 2017 last health care professional visit more than 1 year but not more than 2 years ago	0.181	0.048	3.742	0.000	0.086	0.276
expenditures miscellaneous 2017 trailer and other attachable campers	-0.017	0.009	-1.947	0.052	-0.034	0.000
expenditures miscellaneous 2017 purchase of motorized camper	0.006	0.006	1.093	0.275	-0.005	0.018
expenditures miscellaneous 2017 winter sports equipment	-0.059	0.071	-0.831	0.406	-0.199	0.081
expenditures miscellaneous 2017 global positioning system devices	-0.006	0.055	-0.116	0.907	-0.114	0.101
health adults 2017 has unmet dental need due to cost	0.433	0.171	2.536	0.011	0.098	0.767
expenditures miscellaneous 2017 film	-0.199	0.163	-1.214	0.225	-0.519	0.122
expenditures miscellaneous 2017 fireworks	0.020	0.026	0.783	0.434	-0.030	0.070
expenditures miscellaneous 2017 visual goods	0.320	0.177	1.805	0.071	-0.027	0.668
expenditures miscellaneous 2017 funeral expenses	0.022	0.014	1.610	0.108	-0.005	0.050
expenditures miscellaneous 2017 dating services	-0.282	0.177	-1.591	0.112	-0.630	0.065
expenditures miscellaneous 2017 child support expenditures	0.005	0.004	1.081	0.280	-0.004	0.014
expenditures miscellaneous 2017 gift to non cu members of stocks bonds and mutual funds	-0.005	0.005	-0.958	0.338	-0.016	0.005

HH=Household
Fam=Family
Pop=Population
Non Fam=Non family
OT=Other
ER=Emergency room
RV=recreational vehicle
Equip=equipment
Misc.=miscellaneous
BCBS=Blue Cross Blue Shield
OOT=Out of town
RIHC=resource intensive healthcare
